# Triple glutathione depletion-augmented disulfide and lipid peroxide accumulation to potentiate disulfidptosis and ferroptosis

**DOI:** 10.7150/thno.137256

**Published:** 2026-09-09

**Authors:** Shulin Du, Moyi Zhang, Hao Zheng, Ran Gao, Jinrong Chen, Rouyu Lin, Jing Li, Mengsi Zhang, Hao Zhang

**Affiliations:** 1 Joint Laboratory of Opto-Functional Theranostics in Medicine and Chemistry, Institute of Translational Medicine, The First Hospital of Jilin University, Changchun 130021, P. R. China.; 2 State Key Laboratory of Supramolecular Structure and Materials, College of Chemistry, Jilin University, Changchun 130012, P. R. China.; 3 Scientific Research Center, China-Japan Union Hospital of Jilin University, Changchun 130033, P. R. China.

**Keywords:** disulfidptosis, ferroptosis, glutathione depletion, redox homeostasis, tumor therapy

## Abstract

The adaptive regulation of abnormal redox homeostasis endows tumor cells with robust survival capabilities, with glutathione (GSH) serving as one of the antioxidant cornerstones, which significantly impairs the efficacy of many regulated cell death-related therapies, including disulfidptosis and ferroptosis.

**Methods:**

Herein, Au nanodot-loaded iron-dithiodiglycolic acid (SS) nanoparticles (FeSS@Au NPs) were engineered to potentiate disulfidptosis and ferroptosis *via* triple GSH depletion for oncotherapy. FeSS NPs were pre-prepared by dominant coordination-driven self-assembly between FeCl_3_ and SS. FeSS@Au NPs were prepared through the *in situ* growth of Au nanodots on FeSS NPs. FeSS@Au NPs were systematically evaluated, including the structure, characteristics, stability and antitumor activity.

**Results:**

Fe^3+^ in FeSS@Au NPs can oxidize GSH into glutathione (GSSG) through redox reaction. SS can deplete GSH through thiol-disulfide exchange. Au nanodots can disrupt cystine-to-cysteine transformation by attenuating nicotinamide adenine dinucleotide phosphate supplement with its glucose oxidase-like activity, thus downregulating GSH synthesis by providing less cysteine. Resulting from GSH depletion, the accumulated disulfides, including GSSG and cystine, can trigger disulfidptosis. Meanwhile, the inhibited glutathione peroxidase 4 activity and elevated ·OH *via* Fenton reaction can promote lipid peroxide accumulation, thereby boosting ferroptosis.

**Conclusions:**

Through a triple GSH depletion strategy, FeSS@Au NPs achieve robust tumor growth inhibition by inducing disulfidptosis and ferroptosis, providing a promising therapeutic approach for efficient tumor treatments.

## Introduction

Tumor cells exhibit remarkable adaptability to resist regulated cell death (RCD)-related therapeutics through precisely regulating multiple intracellular homeostasis mechanisms, involving redox balance, metabolic reprogramming, endoplasmic reticulum stress responses, and other pathways 1-4. The redox homeostasis plays a key role in alliance with other homeostasis to intertwine as an integrated defense system that enables tumor cells to resist RCDs 5,6. Reduced glutathione (GSH), as one of the antioxidant cornerstones, plays an irreplaceable role in maintaining intracellular redox balance by scavenging reactive oxygen species (ROS), neutralizing oxidative stress, and regulating the activity of redox-sensitive signaling molecules 7-9. Moreover, GSH also interacts with other intracellular homeostasis systems, including participating in metabolic reprogramming by regulating the activity of metabolic enzymes 10,11, modulating endoplasmic reticulum stress responses by scavenging ROS generated during protein misfolding 12, and cooperating with anti-apoptotic signaling pathways to further strengthen the anti-RCD ability of tumor cells 13,14. Accumulating evidence has shown that tumor cells often exhibit abnormal upregulation of GSH synthesis and metabolism, which is closely associated with their enhanced resistance to RCDs, especially the widely concerned disulfidptosis and ferroptosis 15,16.

Disulfidptosis, a newly identified RCD, is characterized by aberrant accumulation of disulfide bonds in cytoskeleton proteins 17,18. In the process of disulfidptosis, the overexpressed cystine transporter solute carrier family 7 member 11 (SLC7A11) of tumor cells mediates massive cellular cystine uptake, which promotes the accumulation of cystine disulfide bonds in the case of insufficient antioxidant capacity 19,20. Together with the decreased nicotinamide adenine dinucleotide phosphate (NADPH), the abnormal disulfide cross-linking leads to the contraction and disintegration of the actin cytoskeleton, ultimately resulting in cell death 21. To resist disulfidptosis, tumor cells employ the strong redox homeostasis to provide reductive NADPH for cystine-to-cysteine transformation* via* the pentose phosphate pathway in glucose metabolism reprogramming 22. Meanwhile, accompanied by the upregulated GSH/oxidized glutathione (GSSG) ratio, the decreased disulfide bonds of GSSG also suppress disulfidptosis. Therefore, the reductive compensation mechanisms confer tumor cells disulfidptosis resistance.

Ferroptosis, an iron-dependent RCD, is driven by dysfunction of the glutathione peroxidase 4 (GPX4) antioxidant system and excessive lipid peroxide (LPO) accumulation, leading to the breakdown of the cellular membrane system 23-25. In the process of ferroptosis, polyunsaturated fatty acids in the cellular membrane are vulnerable to being oxidized into LPO by over-produced ROS, which rapidly disrupts cell membrane integrity and induces ferroptosis 26. However, tumor cells dynamically evolve with diverse antioxidant defense systems to effectively resist ferroptosis 27. For example, tumor cells can upregulate SLC7A11 to enhance cystine uptake for GSH synthesis, thus promoting the reduction process of LPO by GPX4 28. Besides, to reduce LPO production at its source, tumor cells can also balance ROS levels through GSH 29. Therefore, GSH-mediated strong redox homeostasis shields tumor cells from the attack of ferroptosis.

To address the therapeutic resistance, it is critical to focus on the GSH-dependent redox homeostasis in tumor cells for enhanced disulfidptosis and ferroptosis 30-33. The prevalent strategies primarily focus on promoting GSH-to-GSSG transformation or inhibiting GSH synthesis 34-36. However, a single-target approach often results in incomplete or transient GSH exhaustion, leading to insufficient disulfide stress and LPO accumulation that fails to trigger robust cell death 37-39. Moreover, tumor cells can mount adaptive antioxidant responses, such as upregulating alternative redox systems or enhancing GSH synthesis, further limiting the therapeutic efficacy of single-target interventions 40,41. Thus, developing a multi-targeted GSH depletion strategy is promising to achieve sustained and robust GSH exhaustion, thereby augmenting both disulfide accumulation and LPO generation to potentiate disulfidptosis and ferroptosis.

Herein, we propose a triple GSH depletion strategy to augment disulfide and LPO accumulation, thereby potentiating disulfidptosis and ferroptosis for effective tumor therapy (Scheme 1). Compared with single GSH depletion, which only achieves limited and unstable intracellular GSH elimination due to compensatory mechanisms, this triple GSH depletion strategy realizes synergistic and thorough GSH depletion through multiple regulatory dimensions. The Au nanodot-loaded iron-dithiodiglycolic acid (SS) nanoparticles (FeSS@Au NPs) are engineered through the assembly of Fe^3+^ and SS, and followed by the *in situ* growth of Au nanodots. FeSS@Au NPs can deplete overexpressed GSH in tumor microenvironment (TME) based on the oxidizability of Fe^3+^ and the thiol-disulfide exchange with SS. Meanwhile, GSH synthesis can also be inhibited. Specifically, the Au nanodots exhibit glucose oxidase (GOx)-like activity, which depletes intracellular glucose and restricts NADPH supplement. Insufficient NADPH disrupts cystine-to-cysteine transformation, leading to cystine accumulation and cysteine depletion that inhibits GSH synthesis. Accompanied by triple GSH depletion, disulfidptosis is triggered by the augmented accumulation of disulfide bonds in both GSSG and cystine. Besides, together with the GSH depletion-mediated GPX4 inactivation, the released Fe^2+^ can react with the H_2_O_2_, overexpressed in TME and further elevated by Au nanodot-catalyzed glucose oxidation, to produce ·OH *via* Fenton reaction, which promotes LPO accumulation and boosts ferroptosis. Consequently, FeSS@Au NPs achieve satisfactory antitumor performance *via* depleting GSH in lung tumor models.

## Materials and Methods

**Chemicals and reagents.** Dithiodiglycolic acid (SS) was purchased from Macklin Biochemical Co., Ltd. Iron chloride hexahydrate (FeCl_3_·6H_2_O), gold chloride trihydrate (HAuCl_4_·3H_2_O), sodium borohydride (NaBH_4_), 5,5-dimethyl-1-pyrroline N-oxide (DMPO), 3,3’,5,5’-tetramethylbenzidine (TMB), peroxidase from horseradish (HRP) and 1,10-phenanthroline monohydrate were bought from Aladdin Industrial Corporation. Ham’s F-12K was purchased from BaiDi Biotechnology Co., Ltd. (BDBIO). Cell counting kit (CCK-8) and GoldBand 3-color regular range protein marker (10-180 kDa) were provided by Yeasen (Shanghai, China). Phosphate buffered saline (PBS) and cell freezing medium were bought from Bioland Biotechnology Co., Ltd. Crystal violet aqueous solution (G1059) and L-cysteine (C0012) were purchased from Beijing Solarbio Science & Technology Co., Ltd. Actin-Tracker Green was obtained from Keygen BioTECH. Reduced glutathione assay kit was provided by Nanjing Jiancheng Bioengineering Institute (NJJCBIO). Live and dead cell double staining kit was bought from Abbkine. Non-reducing protein loading buffer was purchased from Beijing Coolaber Science & Technology Co., Ltd. RIPA lysis buffer was bought from New Cell & Molecular Biotech. Hoechst 33342, methylene blue (MB), 2’,7’-dichlorodihydrofluorescein diacetate (DCFH-DA), C11-BODIPY^581/591^, reduced GSH, ferrostatin-1 (Fer-1), tris(2-carboxyethyl)phosphine hydrochloride (TCEP), disodium terephthalate (TA), desferrioxamine (DFO), ethylenediaminetetraacetic acid (EDTA), NaCl, DMSO, glucose, Ac-DEVD-CHO, chloroquine and TBS-T powder were provided by MedChemExpress. Prestained protein marker (Triple color, EDTA free, 10-250 kDa, F4005) was bought from APExBIO (Houston, USA). GSH and GSSG assay kit, BCA protein assay kit and NADP^+^/NADPH assay kit were provided by Beyotime Biotechnology Co., Ltd. Rabbit anti-GPX4 polyclonal antibody was bought from Bioss. GPX4 activity assay kit was bought from Beijing Boxbio Science & Technology Co., Ltd. All reagents were used as received without additional purification.

**Preparation of FeSS NPs and FeSS@Au NPs.** FeSS NPs are prepared through adding 5 mL SS (13.5 mg/mL in H_2_O) and 5 mL FeCl_3_·6H_2_O (12.5 mg/mL in H_2_O) into 90 mL H_2_O with stirring. Then the mixed solution keeps stirring at room temperature. After 10 min, FeSS NPs are collected by centrifugation at 8800 rpm for 10 min. The obtained FeSS NPs are dispersed in H_2_O for subsequent modification. To prepare FeSS@Au NPs, 1.8 mL HAuCl_4_ (8 mM) and 0.6 mL NaBH_4_ (50 mM) are added to FeSS NPs solution and stirred for 5 min. Then FeSS@Au NPs are obtained after being washed three times with H_2_O.

**Calculation of component proportions in FeSS NPs and FeSS@Au NPs.** (1) Initially, the proportions of Fe and SS in FeSS NPs are calculated. The mass fraction of Fe is measured as 22.1% by the inductively coupled plasma atomic emission spectrometry (ICP-AES). The mass fraction of SS is 73.2% according to the C element mass fraction by elemental analyser. The mass fraction of H_2_O is 1.2% according to the H element mass fraction, excluding the H element in SS. The remaining 3.5% mass fraction is attributed to Cl. (2) The proportions in FeSS@Au NPs are calculated based on the proportions in FeSS NPs. The mass fraction of Au is measured as 13.0% by ICP-AES. Therefore, FeSS in FeSS@Au NPs is 87.0%. Fe and SS are calculated as 19.2% and 63.7%, respectively. (3) To further analyze the Fe^2+^ and Fe^3+^ mass fractions, the 1,10-phenanthroline colorimetric assay is conducted, measuring the mass fraction of Fe^2+^ as 2.3%. The mass fraction of Fe^3+^ is 16.9% through the sum Fe mass fraction (19.2%), excluding Fe^2+^ mass fraction. The remaining 4.1% mass fraction is attributed to others, including Cl and H_2_O.

**GOx-like activity and kinetic studies of FeSS@Au NPs.** The GOx-like activity of FeSS@Au NPs was studied by monitoring the levels of O_2_, pH, glucose and H_2_O_2_. GOx (25 ng/mL) combined with glucose served as the positive control. The FeSS@Au NPs (0-200 μg/mL) were incubated with glucose (2 mg/mL) at 37 °C. During the incubation of FeSS@Au NPs (100 μg/mL), the O_2_ content was revealed by a portable dissolved oxygen meter. The pH of the solutions was also monitored by using a pH meter over time to reflect the produced gluconic acid. After 30 min, the supernatant of the solutions was collected by centrifugation for further measurements. The glucose content was measured by using glucose assay kit. The generation of H_2_O_2_ was explored by adding TMB and HRP. Then the absorption spectra of the mixture were measured.

**Exploration of iron ion release.** The FeSS@Au NPs were incubated in pH 5.5 solution containing 2 mM GSH and 100 μM H_2_O_2_ from 0 to 48 h. The supernatant was collected after centrifugation at 13000 rpm for 10 min. Thereafter, the Fe^2+^ level in the supernatant was detected by using 1,10-phenanthroline colorimetric assay.

**Detection of ·OH.** The concentrations involved are as follows: glucose (2 mg/mL), GSH (2 mM), H_2_O_2_ (100 μM), FeSS NPs (100 μg/mL), FeSS@Au NPs (100 μg/mL). (i) In the DMPO method, different groups were incubated at 37 °C. Then, DMPO was added for the electron spin resonance (ESR) test. (ii) In the MB method, MB solutions were incubated with different groups at 37 °C for 20 h. Besides, MB solutions were also incubated with FeSS@Au NPs + GSH + H_2_O_2_ + Glucose at 37 °C for varied durations. After all, the supernatant was obtained for an ultraviolet-visible (UV-vis) absorption spectrum acquisition. (iii) In the TA method, the FeSS@Au NPs were incubated with glucose, GSH and H_2_O_2_ in TA solution at 37 °C for fluorescence spectra measurement.

**Cytotoxicity assay.** The cells were seeded in 96-well plates and incubated for 24 h. Then, the NPs of varied concentrations were added for another incubation of 24 h. The CCK-8 reagent was added for tests by using a microplate reader.

**Cell live/dead staining assay.** The human non-small cell lung cancer (A549) cells were incubated with FeSS@Au NPs (0-150 μg/mL) for 12 h. Then the cells were stained by using the live and dead cell double staining kit according to the protocol. After staining, the fluorescence images were captured by a confocal laser scanning microscope (CLSM).

**WB analysis.** The western blotting (WB) was processed in compliance with the standard protocol. The protein samples were extracted from A549 cells and normal human lung epithelial (BEAS-2B) cells. After that, the proteins were quantified and normalized to a consistent concentration. The protein samples were heated at 100 °C for 5 min for WB. Finally, the WB data were obtained by chemiluminescence imaging.

**Intracellular GSH and GSSG assays.** The A549 cells were incubated with FeSS NPs (0-150 μg/mL) and FeSS@Au NPs (0-150 μg/mL) for 24 h, respectively. Then, the cells were washed and lysed at 4 °C. The supernatant was collected for GSH and GSSG tests according to the standard protocol of the GSH and GSSG assay kit. Quantitative analysis was conducted based on the protein concentrations measured by the BCA protein assay kit.

**Cytoskeletal protein observation.** The A549 cells were seeded in confocal dishes and incubated with FeSS NPs (100 μg/mL), FeSS@Au NPs (0-100 μg/mL) and FeSS@Au NPs (100 μg/mL) + TCEP (2 mM) for 12 h, respectively. Then, the cells were fixed with 4% paraformaldehyde and stained with Actin-Tracker Green in the dark. The cell nuclei were stained with Hoechst 33342. Then, the cells were washing with PBS containing 0.1% Triton X-100 for cytoskeletal protein observation.

**Intracellular ROS assay.** For group exploration, the A549 cells were treated with FeSS NPs (100 μg/mL), FeSS@Au NPs (100 μg/mL), and FeSS@Au NPs (100 μg/mL) + DFO (100 μM) for 6 h. For concentration exploration, the A549 cells were incubated with FeSS@Au NPs (0-100 μg/mL) for 12 h. After incubation, the cells were stained with DCFH-DA for CLSM observation.

**Intracellular LPO measurement.** The A549 cells were treated with FeSS NPs (100 μg/mL), FeSS@Au NPs (0-100 μg/mL), and FeSS@Au NPs (100 μg/mL) + Fer-1 (15 μM) for 6 h. After incubation, the cells were stained with C11-BODIPY^581/591^ for CLSM observation.

**Fluorescence imaging.** Initially, the FeSS@Au NPs were labeled with indocyanine green (ICG) for fluorescence imaging. For the *in vivo* explorations, the mice bearing subcutaneous A549 tumors were administrated with ICG-FeSS@Au NPs *via* the tail vein. At the designated time, the fluorescent images of blood, body, organs, tumors, feces and urine were captured by using a fluorescence imager under an 808 nm laser.

**Hemolysis assay.** The FeSS@Au NPs of varied concentrations (0, 50, 100, 150 and 200 μg/mL) were incubated in PBS solutions containing 2% red blood cells at 37 °C for 1 h. Meanwhile, the H_2_O group and 0 group were conducted as the positive control and negative control. Then, the supernatant was collected and the absorbance at 541 nm (A) was measured for hemolysis rate calculation. Hemolysis rate (%) = (A_sample_-A_negative_) / (A_positive_-A_negative_) × 100%.

**Animals.** All animal experimental procedures were carried out in strict compliance with the ethical guidelines formulated by the Animal Laboratory Ethics Committee of the First Hospital of Jilin University (Approval numbers: JDYY20250845 and JDYY20261259). Four-week-old female BALB/c nude mice were bought from Beijing Vital River Laboratory Animal Technology Co., Ltd. Following one-week adaptive period, the mice were subcutaneously injected with 5000000 A549 cells on the right buttocks. After 8 days, five experimental groups were set, including (I) Control, (II) FeSS NPs, (III) FeSS@Au NPs, (IV) FeSS@Au NPs + Fer-1, and (V) FeSS@Au NPs + TCEP. On the 0^th^, 4^th^, 8^th^ and 12^th^ days, all the NPs (2 mg/mL, 100 μL) were injected *via* the tail vein, and the Fer-1 (160 μM, 25 μL) and TCEP (80 mM, 25 μL) were injected into the tumor tissues. The tumor volume (V) and body weight of the mice were recorded every 2 days (V = 1/2 × L × W^2^, L and W represent the length and width of the tumor). On the 20^th^ day, the mice were photographed and sacrificed to obtain heart, liver, spleen, lungs, kidneys and tumors for the hematoxylin and eosin (H&E) analysis. Meanwhile, the blood lipid, blood glucose, liver and kidney function tests were also measured.

**Statistical analysis.** All quantitative data were shown as mean ± standard deviation (SD). Unpaired two-tailed Student’s *t*-test was applied for data analysis between two groups. One-way analysis of variance (ANOVA) followed by a post-hoc multiple comparison test was applied for multigroup statistical analysis. *P < 0.05, **P < 0.01, ***P < 0.001, and ****P < 0.0001.

## Results and Discussion

**Preparation and characterization of FeSS@Au NPs.** FeSS NPs are pre-prepared by adding SS into FeCl_3_ aqueous solution with gentle stirring, which exhibit spherical morphology under transmission electron microscopy (TEM) (Figure 1A). By altering the amount of SS, the diameters of FeSS NPs can be tailored from 40 to 130 nm (Figure S1 and Table S1). Considering the uniformity and dispersibility, the FeSS NPs with ~60 nm in diameter are employed for further studies (Figure 1A and Figure S2). Regarding the components, the existence of SS in FeSS NPs is revealed by mass spectra (Figure 1B). SS is calculated as ~70% according to the elemental analyser (Table S2 and Figure S3). ICP-AES suggests 22.1% Fe in FeSS NPs (Table S2 and Figure S3), which is all assigned to Fe^3+^ (Figure S4). The Fe-to-SS molar ratio is approximately 1:1 (Table S2).

To further analyze the structure, the Fourier transform infrared (FTIR) spectra are measured. As shown in Figure 1C, the Fe-O peak around 580 cm^-1^ and the peak of coordinated carboxyl around 1596 cm^-1^ demonstrate the existence of Fe-O coordination. The shift of C-S stretching vibration peaks (Figure 1C) and the high-resolution X-ray photoelectron spectroscopy (XPS) S 2p spectrum (Figure S5) jointly confirm the Fe-S coordination. Therefore, in FeSS NPs, Fe^3+^ coordinates with both S-S and C=O groups of SS. The main driving force in forming FeSS NPs is also investigated through introducing specific reagents to break non-covalent bonds, including EDTA for coordination bond, NaCl for electrostatic interaction, Triton X-100 for hydrophobic interaction, urea for hydrogen bond, and DMSO for π-π interaction. As shown in Figure S6, FeSS NPs disassemble in EDTA, NaCl and urea solutions, which reflects the essential coordination bond, electrostatic interaction and hydrogen bond in forming FeSS NPs. Specifically, Fe^3+^ coordinates with SS, and electrostatically interacts with the ionized -COO^-^ of SS, thus assembling into the nanoformulations. Meanwhile, the hydrogen bonds between SS molecules also contribute to the formation of FeSS NPs. Combining with the Fe-to-SS molar ratio in FeSS NPs, the proposed structure is illustrated in Figure S7.

FeSS@Au NPs are prepared through the *in situ* growth of Au nanodots on FeSS NPs using NaBH_4_ to reduce HAuCl_4_ (Figure 1D and Figure S8A). As revealed by TEM, the Au nanodots are homogeneously distributed on the surface of FeSS@Au NPs with the diameter around 2.4 nm (Figure 1E and Figure S8B). The hydrated diameters of FeSS NPs and FeSS@Au NPs are 208.8 nm and 376.7 nm, respectively (Figure 1F), which are larger than those in TEM images, originating from the surface hydrated layer on the NPs and solution environment effects. The zeta potential decreases from 17.5 to 2.4 mV after decorating with Au nanodots (Figure 1G), which may derive from the residual Cl^-^. The X-ray diffraction (XRD) patterns (Figure 1H), XPS spectra (Figure 1I-J) and the elemental line-scan mapping images (Figure S9 and Figure S10) collectively demonstrate the existence of Au. The Fe 2p spectra indicate the coexistence of Fe^2+^ and Fe^3+^ (Figure 1K). The existence of Fe^2+^ is attributed to the partial reduction of Fe^3+^ by NaBH_4_ in the growth of Au nanodots. Based on the component data of FeSS NPs, the mass fraction of all components in FeSS@Au NPs is investigated by supplemental ICP-AES measurement for Au and 1,10-phenanthroline colorimetric assay for Fe^2+^ (Figure S4). As shown in Figure 1L, the composition of FeSS@Au NPs is approximately 63.7% SS, 2.3% Fe^2+^, 16.9% Fe^3+^ and 13.0% Au.

**Responsive disassembly of FeSS@Au NPs.** To reveal the potential in tumor therapy, the colloidal and structural stabilities of FeSS@Au NPs are evaluated first. With incubation in H_2_O and culture medium for 0 and 20 days, the Tyndall effect of FeSS@Au NPs indicates the good colloidal stability (Figure S11A). The hydrated diameters remain unchanged, which demonstrates the good structural stability of FeSS@Au NPs (Figure S11B). Thereafter, FeSS@Au NPs are incubated with glucose solution to explore the GOx-like activity. As shown in Figure 2A, the glucose content decreases with the increment of FeSS@Au NPs. The reduced oxygen content (Figure 2B), the appearance of H_2_O_2_ (Figure 2C), and the decreased pH (Figure 2D) are also observed, which reflects the good GOx-like activity of FeSS@Au NPs. This capability endows FeSS@Au NPs with the potential to reduce NADPH supplement by depleting glucose.

The responsive disassembly is also explored, which largely determines the performance of FeSS@Au NPs. As indicated in Figure S12A, most FeSS@Au NPs disassemble in GSH solution, which is contributed by the reduction-responsive cleaved disulfide bonds in SS, as well as the reduction of Fe^3+^ to Fe^2+^ by GSH. Besides, FeSS@Au NPs also disassemble in H_2_O_2_ solution, possibly due to the Fenton reaction and the peroxidase-like activity of Fe^2+^ and Fe^3+^ in FeSS@Au NPs (Figure S12B). As for the pH 5.5 solution, the main structure of FeSS NPs remains, but some Au nanodots slightly fall from the NPs (Figure S12C), possibly due to the attenuated electrostatic attraction between FeSS NPs and adsorbed Au nanodots 42. Nevertheless, compared with a physiological pH 7.4 aqueous solution (Figure S12D), a significant disassembly of FeSS@Au NPs occurs in the solution containing GSH and H_2_O_2_ at pH 5.5 (Figure 2E), which demonstrates the good capability of TME-responsive disassembly.

The GSH-mediated cleavage of the disulfide bond in SS and reduction of Fe^3+^ to Fe^2+^ lead to the effective disassembly of FeSS@Au NPs. Meanwhile, massive GSH is consumed during this process (Figure 2F). Considering the interference from -SH of cleaved SS, the actual GSH level is lower than the measured one by ultra-performance liquid chromatography-mass spectrometry (UPLC-MS) (Figure S13). The significantly decreased GSH and increased GSSG (Figure S14) are beneficial for GSH level-directed therapy. The release of Fe^2+^ is also studied under the stimulated TME. As shown in Figure 2G, over 60% iron ions are released from FeSS@Au NPs, which are in the form of Fe^2+^. The released Fe^2+^ is supposed to produce ·OH through Fenton reaction 43. So, the ·OH is subsequently measured with DMPO on ESR, MB on UV-vis spectrophotometer, and TA on a fluorescence spectrophotometer, which evidences the production of ·OH (Figure 2H-K). Such TME-responsive FeSS@Au NPs with both GOx-like activity and ·OH-producing capability are good candidates for further antitumor applications (Figure 2L).

**Triple GSH depletion by FeSS@Au NPs.** The cellular uptake and endocytosis explorations are essential for further intracellular activities of FeSS@Au NPs. A549 cells are employed for* in vitro* and *in vivo* investigations. The uptake of FeSS@Au NPs by A549 cells is first investigated by measuring the relative Fe content by ICP-AES (Figure S15), which indicates effective endocytosis. Subsequently, the appearance of intracellular Fe^2+^ from the GSH-responsive disassembled FeSS@Au NPs also indicates the uptake of FeSS@Au NPs by A549 cells (Figure 3A-B). To further explore the intracellular delivery behavior and therapeutic potential of FeSS@Au NPs, the lysosomal escape capability is systematically investigated. As shown in Figure S16A-B, FeSS@Au NPs can enter and escape from lysosomes. Furthermore, the colocalization of the released Fe^2+^ from FeSS@Au NPs and lysosomes is also explored. As shown in Figure S16C-D, orange Fe^2+^ fluorescence gradually increases from 0 to 8 h, accompanied by the maximum Pearson’s correlation coefficient at 4 h, indicating that FeSS@Au NPs can disassemble both in lysosomes and the cytoplasm, which benefits thorough GSH depletion.

As a proof-of-concept, the GSH depletion by FeSS@Au NPs is studied. The decreased glucose level reflects the good GOx-like activity of Au nanodots on FeSS@Au NPs (Figure 3C). Moreover, the intracellular NADPH level is also attenuated (Figure 3D), which is dedicated to suppressing GSH biosynthesis by blocking cystine-to-cysteine transformation on the SLC7A11/GSH/GPX4 antioxidant axis. To evaluate the independent contribution of different components, the GSH depletion is measured after the treatments of Fe³⁺, SS, Au nanodots and FeSS@Au NPs. The total GSH depletion rate of the above three components (Figure S17) is lower than that of FeSS@Au NPs (Figure 3E). After all, contributed from both the massive consumption by reducing Fe^3+^ and SS, and the inhibited synthesis by blocking cystine-to-cysteine transformation, the GSH level is largely downregulated (Figure 3F), which also proves the speculation above that FeSS@Au NPs can access the cytoplasmic GSH pool. These single GSH depletion strategies only achieve limited and unstable intracellular GSH elimination due to compensatory mechanisms, which implies possible synergy in FeSS@Au NPs.

Given that GSH serves as the key antioxidant molecule for clearance of intracellular ROS, and that the antioxidant function of GPX4 is highly dependent on GSH reduction, the FeSS@Au NP-mediated GSH depletion inevitably blocks the SLC7A11/GSH/GPX4 antioxidant axis, disrupting the redox homeostasis of A549 cells. Persistent oxidative stress is a key driver inducing tumor cell death (Figure S18). Therefore, the therapeutic potential of FeSS@Au NPs is further evaluated. First, the cytotoxicity is evaluated through live/dead staining and CCK-8 assays. As shown in Figure 3G, the number of A549 cells with red fluorescence increases, while those with green fluorescence decrease with the elevated concentration of FeSS@Au NPs, which indicates the effective lethality. For quantitative analysis, cell viability is measured according to CCK-8 assays. There are over 70% cells killed by 100 μg/mL FeSS@Au NPs and the half maximal inhibitory concentration (IC50) of FeSS@Au NPs is calculated as 36.9 μg/mL (Figure S19A), showing strong potential in oncotherapy. Besides, the cytotoxicity of FeSS NPs is merely half of FeSS@Au NPs (Figure S19B), which is mainly attributed to the settled glucose level and the slightly diminished GSH level (Figure S20). The above results demonstrate that FeSS@Au NPs disrupt redox homeostasis by targeting GSH, ultimately achieving high efficiency in killing A549 cells.

Moreover, the cell viability of BEAS-2B cells remains nearly 70% after incubation with 100 μg/mL FeSS@Au NPs (Figure S21), exhibiting a selective cytotoxicity, which may derive from the significant difference in GSH level and SLC7A11 expression between normal cells and cancerous cells. Additionally, the relatively low GSH level in normal cells may restrict the TME-responsive disassembly of FeSS@Au NPs (Figure S22), thereby impeding the activation of therapeutic effects. Meanwhile, SLC7A11 expression is less in BEAS-2B cells, which is almost half of A549 cells (Figure S23). Therefore, targeting GSH to disrupt SLC7A11/GSH/GPX4 antioxidant axis hardly induces disulfidptosis in normal cells. Such selectivity is critical for minimizing off-target toxicity and enhancing the therapeutic efficacy of FeSS@Au NPs, highlighting the potential as safe and effective nanoformulations for tumor treatments.

**Disulfidptosis and ferroptosis for *in vitro* antitumor.** To elucidate the specific cell death mechanism by FeSS@Au NPs, the TEM images of A549 cells are captured for initial evaluation. As shown in Figure 4A, typical characteristics of disulfidptosis and ferroptosis appear after incubation with FeSS@Au NPs. The shrunken cell, reduced size and the fuzzy cellular structure jointly imply the occurrence of disulfidptosis. Meanwhile, there are some hallmarks of ferroptosis, including the shriveled mitochondrial membrane, the reduced mitochondrial cristae and broken cellular membrane. For further quantitative analysis, specific inhibitors are employed. As shown in Figure 4B and Figure S24, cell death is partially rescued with disulfidptosis inhibitor, TCEP, ferroptosis inhibitor, Fer-1, and the combination of TCEP and Fer-1. Besides, other cell death pathways, such as autophagy and apoptosis, are ruled out because of the hardly increase in cell viability after incubation with corresponding inhibitors, including chloroquine and Ac-DEVD-CHO (Figure S25). Therefore, disulfidptosis and ferroptosis play the main role in killing cells, which contribute ~40% and ~30%, respectively (Figure 4C).

Furthermore, the characterizations of disulfidptosis and ferroptosis in other cell lines are also compared with A549 cells, including normal cells (BEAS-2B) and tumor cells (human non-small cell lung cancer cells, H1299) with low expression of SLC7A11. As shown in Figure 4B-C and Figure S26, A549 cells are inhibited more significantly than BEAS-2B cells and H1299 cells, which may derive from the substantially higher basal GSH levels in A549 cells compared to BEAS-2B and H1299 44. In contrast, BEAS-2B and H1299 cells exhibit reduced sensitivity to disulfidptosis owing to low SLC7A11 expression, which restricts cystine transport and suppresses disulfide-bond cleavage. On the other hand, the low intracellular GSH levels in BEAS-2B and H1299 cells inhibit GPX4 activity and exacerbate lipid peroxidation, thereby leading to an increased ferroptosis-to-disulfidptosis ratio compared with that in A549 cells. Therefore, both the GSH level and SLC7A11 expression have an essential effect on the characterizations of disulfidptosis and ferroptosis.

To investigate disulfidptosis further, the levels of representative disulfides are measured, including cystine and GSSG. As shown in Figure 4D and Figure S27A, the levels of cystine and GSSG increase after incubation with incremental FeSS@Au NPs. Subsequently, a typical characteristic of disulfidptosis, cross-links between macromolecular proteins formed by the intermolecular disulfide bonds, is evaluated through WB. With FeSS@Au NPs treatment, myosin heavy chain 9 (MYH9), one of the actin cytoskeleton proteins, exhibits significant gel migration retardation with extremely high molecular weight bands appearing near the stacking zone under the non-reducing condition (Figure 4E). After introducing the reducing agent, TCEP, the molecular weight bands return to ~230 kDa, which indicates the disulfide bonds in MYH9.

Furthermore, to explore the macroscopic characteristic of disulfidptosis, the shrinkage of the actin cytoskeleton of A549 cells is observed through CLSM. As shown in Figure 4F, after incubation with FeSS NPs, the cells appear smaller and the actin cytoskeleton shrinks, which is attributed to the increased GSSG due to the oxidization of GSH by Fe^3+^ and SS (Figure S27). In comparison, the change of cellular morphology is more pronounced for FeSS@Au NP-treated group, which derives from the introduced Au nanodots. Specifically, the GOx-like activity of Au nanodots can block cystine-to-cysteine transformation (Figure S28), then elevate the cystine level (Figure 4D), thus augmenting disulfidptosis. The change of cellular morphology is alleviated after incubation with additional TCEP for reducing disulfide bonds. In addition, FeSS@Au NPs trigger disulfidptosis in a concentration-dependent manner (Figure 4G and Figure S29).

FeSS@Au NPs also show the capability to induce ferroptosis. On the one hand, as mentioned above (Figure 3A and Figure S18), massive Fe^2+^ is released from FeSS@Au NPs in A549 cells, which can efficiently produce ·OH through Fenton reaction (Figure 5A-B and Figure S30). As one of the most potent oxidizing species, ·OH can attack lipids in membrane structures and initiate LPO accumulation, thus triggering ferroptosis. On the other hand, the enhanced GSH depletion by FeSS@Au NPs significantly downregulates GPX4 expression (Figure 3E, Figure 5C-D and Figure S31), which suppresses the clearance of LPO. Concurrently, Fe^2+^ continuously triggers LPO accumulation by promoting LPO generation coupled with attenuated LPO clearance (Figure 5E, Figure S32 and Figure S33). Lipid peroxidation also exhibits FeSS@Au concentration-dependent increment (Figure S34). Consequently, the LPO within the cell membrane impairs membrane integrity (Figure 5F), which is the well-recognized hallmark of ferroptosis. Compared with FeSS NPs, FeSS@Au NPs exhibit amplified ferroptosis due to the enhanced GSH-depletion by introducing Au nanodots (Figure 5), which reflects the efficiency of GSH-targeted ferroptosis. The colony formation assay exhibits a dose-dependent inhibitory effect of FeSS@Au NPs on cell proliferation (Figure S35), implying good potential for antitumor.

***In vivo* investigations.** The pharmacokinetic behavior of FeSS@Au NPs is evaluated by tracking the absorption, distribution and excretion through fluorescence imaging. FeSS@Au NPs are labeled with ICG for further analysis (Figure S36 and Figure S37). After systemic administration *via* tail vein injection, ICG-FeSS@Au NPs exhibit a good blood circulation half-life (t_1/2_ = 4.4 h) in BALB/c nude mice subcutaneously bearing A549 tumors (Figure 6A), which is beneficial for enhancing their passive accumulation ability toward tumor cells *via* the enhanced permeability and retention (EPR) effect. Meanwhile, the biodistribution in major organs, including heart, liver, spleen, lungs and kidneys shows the highest amounts at 4 h post-injection with the subsequent decreasing from 8 to 144 h, which may be related to the slow degradation of NPs after reticuloendothelial system capture and good metabolic clearance (Figure 6B-D). Driven by the EPR effect, massive FeSS@Au NPs accumulate in tumor tissues with the highest amount observed at 4 h post-injection and last for more than 2 days (Figure 6B-D). The long-time accumulation facilitates the sustained release of bioactive components for effective therapy. Excretion assessments were performed by collecting the feces and urine at varied periods. As shown in Figure 6E, the fluorescence intensity of feces exhibits a more pronounced increase than that of urine from 0 to 12 h, implying that biliary excretion serves as the primary excretion pathway, supplemented by renal excretion. Meanwhile, the subsequent decrease of fluorescence intensity from 12 to 96 h confirms the effective elimination of FeSS@Au NPs, demonstrating favorable biocompatibility and biosafety. Collectively, the pharmacokinetic profile of FeSS@Au NPs characterizes with long blood circulation, tumor-targeted accumulation, and efficient excretion, which lays a solid foundation for further antitumor therapy.

To further validate the therapeutic efficacy of FeSS@Au NPs, the *in vivo* antitumor experiments are conducted on BALB/c nude mice bearing subcutaneous A549 tumors. The mice are divided into five random groups, including (I) Control, (II) FeSS NPs, (III) FeSS@Au NPs, (IV) FeSS@Au NPs + Fer-1, and (V) FeSS@Au NPs + TCEP (Figure 7A). All groups are administrated with corresponding formulations *via* tail vein injection on the 0^th^, 4^th^, 8^th^ and 12^th^ days (Figure 7B). The tumor volume and body weight of the mice are monitored every 2 days throughout the 20-day experiment period. Notably, group III exhibits the most significant tumor growth inhibition effect, at over 80% (Figure 7C-G and Figure S38A). Meanwhile, the tumor growth inhibition effect of FeSS@Au NPs is significantly attenuated in groups IV and V, with the growth inhibition dropping to 44.5% and 33.5%, respectively. The alleviation of therapeutic efficacy with the co-administration of Fer-1 and TCEP clarifies the dominant cell death pathways (Figure S39A), including ferroptosis and disulfidptosis. Furthermore, the GSH decrease (Figure S40A), disulfide increase (Figure S40B-C), GPX4 downregulation (Figure S40D) and LPO augmentation (Figure S40E) are also assessed, which exhibit effective GSH-depletion-induced disulfidptosis and ferroptosis. The H&E stained tumor slices also demonstrate the good efficacy of FeSS@Au NPs (Figure S41). Ki67 immunohistochemical staining confirms robust suppression of tumor cell proliferation (Figure S38B). This finding directly confirms that FeSS@Au NPs exert antitumor activity by simultaneously triggering ferroptosis and disulfidptosis in tumor cells, highlighting the superiority of FeSS@Au NPs in synergistic tumor therapy.

A series of experiments is carried out to explore the biosafety of FeSS@Au NPs, focusing on blood and organ biocompatibility. To corroborate the blood biocompatibility of FeSS@Au NPs, which is critical for intravenous administration, a hemolysis assay is performed. The result shows the hemolysis rate of 2% in the concentration range of 0 to 200 μg/mL, which is much lower than the 5% threshold universally recognized for biocompatible biomaterials (Figure 7H) 45. Meanwhile, the blood lipid (CHO and TG) and blood glucose (GLU) results also demonstrate the good biocompatibility (Figure S42). To further verify the *in vivo* organ biocompatibility of FeSS@Au NPs, liver and kidney function tests are performed by detecting serum biomarkers, followed by histological observation of major organs. As shown in Figure S43, no obvious differences in all measured indexes are found among groups I to V, including the liver function biomarkers (ALT, AST, TBIL and ALP) and kidney function biomarkers (BUN and UA). Besides, no obvious pathological damages are found in the major organs (hearts, livers, spleens, lungs and kidneys) of mice treated with FeSS@Au NPs (Figure S44). Together with the stable body weight of the treated mice (Figure S45), these results fully confirm the favorable biosafety of FeSS@Au NPs, highlighting the potential for antitumor applications. To further dissect dose-dependent effects, parallel* in vivo* tests with additional groups are implemented. Three escalating-dose FeSS@Au NP groups (1, 3, 4 mg/mL) are supplemented. Tumor inhibition by FeSS@Au NPs increases in a dose-dependent manner (Figure S39A), indicating favorable therapeutic efficacy. The body weight (Figure S39B), liver function biomarkers and kidney function biomarker (Figure S46) confirm the favorable biosafety of FeSS@Au NPs.

## Conclusions

In conclusion, this study indicates a triple GSH depletion strategy to augment disulfide and LPO accumulation, thereby synergistically enhancing disulfidptosis- and ferroptosis-mediated antitumor efficacy. As the key to this strategy, FeSS@Au NPs enable efficient GSH depletion by integrating the oxidizability of both Fe^3+^ and SS, and Au nanodot-mediated blocking of cystine-to-cysteine transformation. As a result, disulfidptosis is triggered by the accumulated cystine and GSSG, and ferroptosis is induced by the elevated ROS level and downregulated GPX4 activity. The *in vitro* experiments validate the remarkable effect of this GSH depletion strategy on redox homeostasis dysregulation, while the *in vivo* evaluations exhibit a great tumor growth inhibition *via* disulfidptosis and ferroptosis. Collectively, the triple GSH depletion strategy overcomes the bottlenecks of incomplete depletion, tumor compensatory resistance and insufficient cell death activation existing in single GSH depletion approaches, verifying the rationality and translational superiority of multi-dimensional metabolic intervention design for robust tumor therapy. This effective GSH depletion strategy significantly enhances disulfidptosis and ferroptosis in tumor treatments, which has the potential for advancing the tumor treatment leveraging redox homeostasis-related RCDs.

## Supplementary Material

Supplementary figures.

## Figures and Tables

**Scheme 1:**
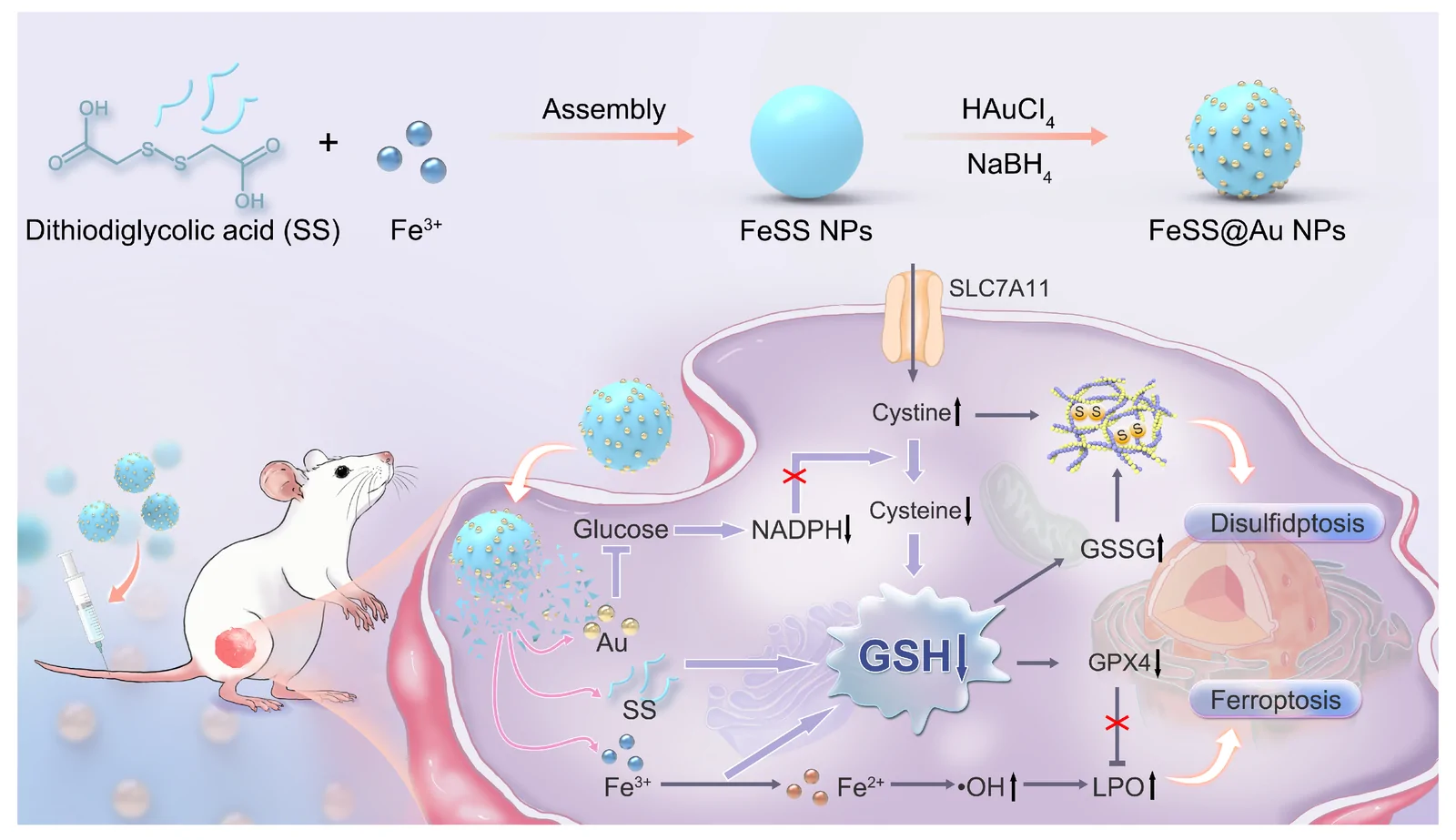
** Scheme of FeSS@Au NPs preparation and therapy mechanism.** Schematic illustration for the preparation of FeSS@Au NPs and the mechanism of triple GSH depletion to augment disulfide and LPO accumulation for boosting disulfidptosis and ferroptosis in tumor therapy.

**Figure 1:**
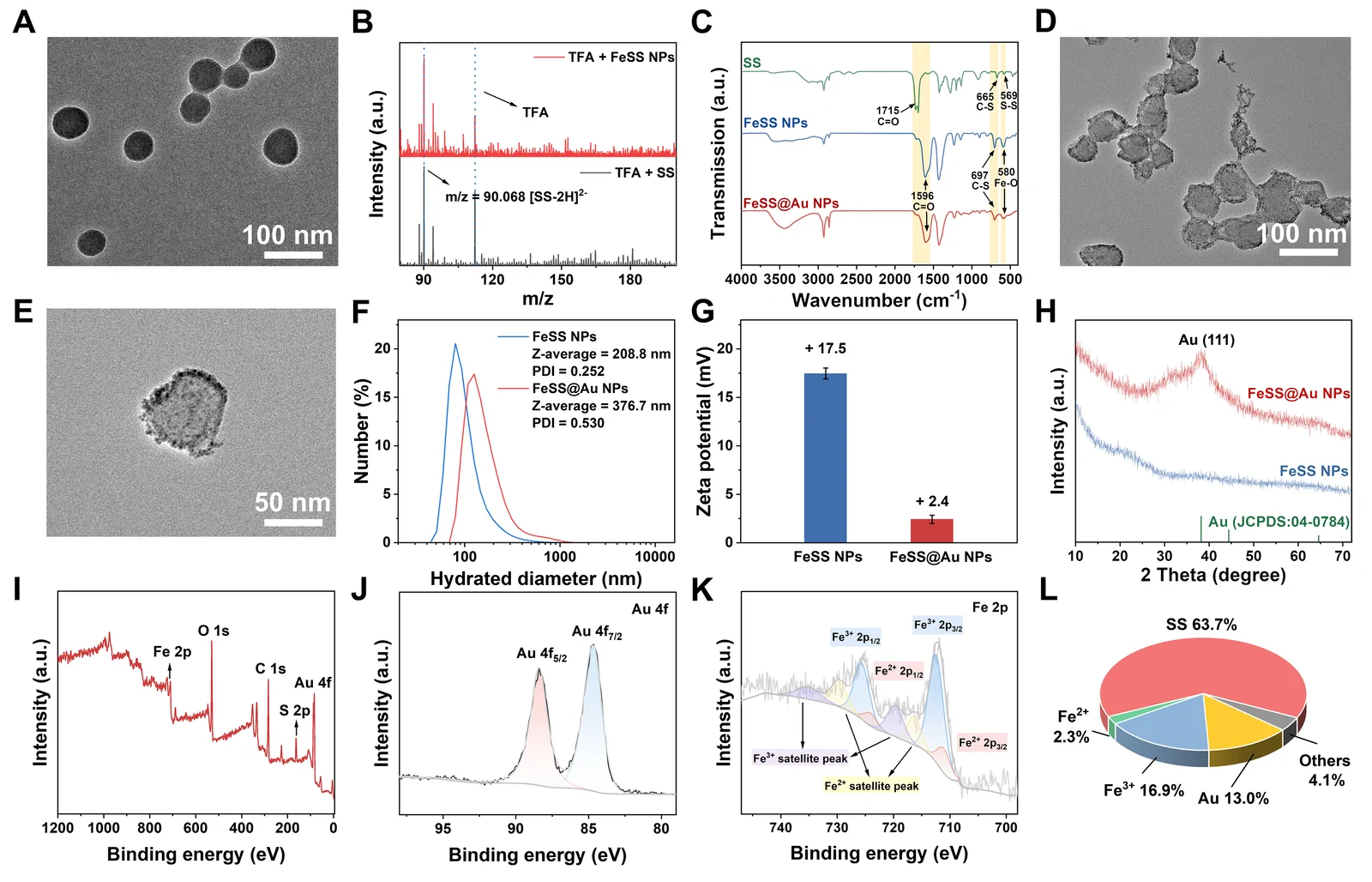
** Characterization of FeSS NPs and FeSS@Au NPs.** (A) The TEM image of FeSS NPs. (B) The mass spectra of SS and FeSS NPs. (C) The FTIR spectra of SS, FeSS NPs and FeSS@Au NPs. (D-E) The TEM images of FeSS@Au NPs. The hydrated diameter distributions (F), and zeta potentials (G) of FeSS NPs and FeSS@Au NPs. (H) The XRD patterns of FeSS NPs and FeSS@Au NPs. The XPS survey spectrum (I), high-resolution Au 4f spectrum (J) and high-resolution Fe 2p spectrum (K) of FeSS@Au NPs. (L) The component analysis of FeSS@Au NPs. The data in Figure 1G are presented as mean ± SD (n = 3).

**Figure 2:**
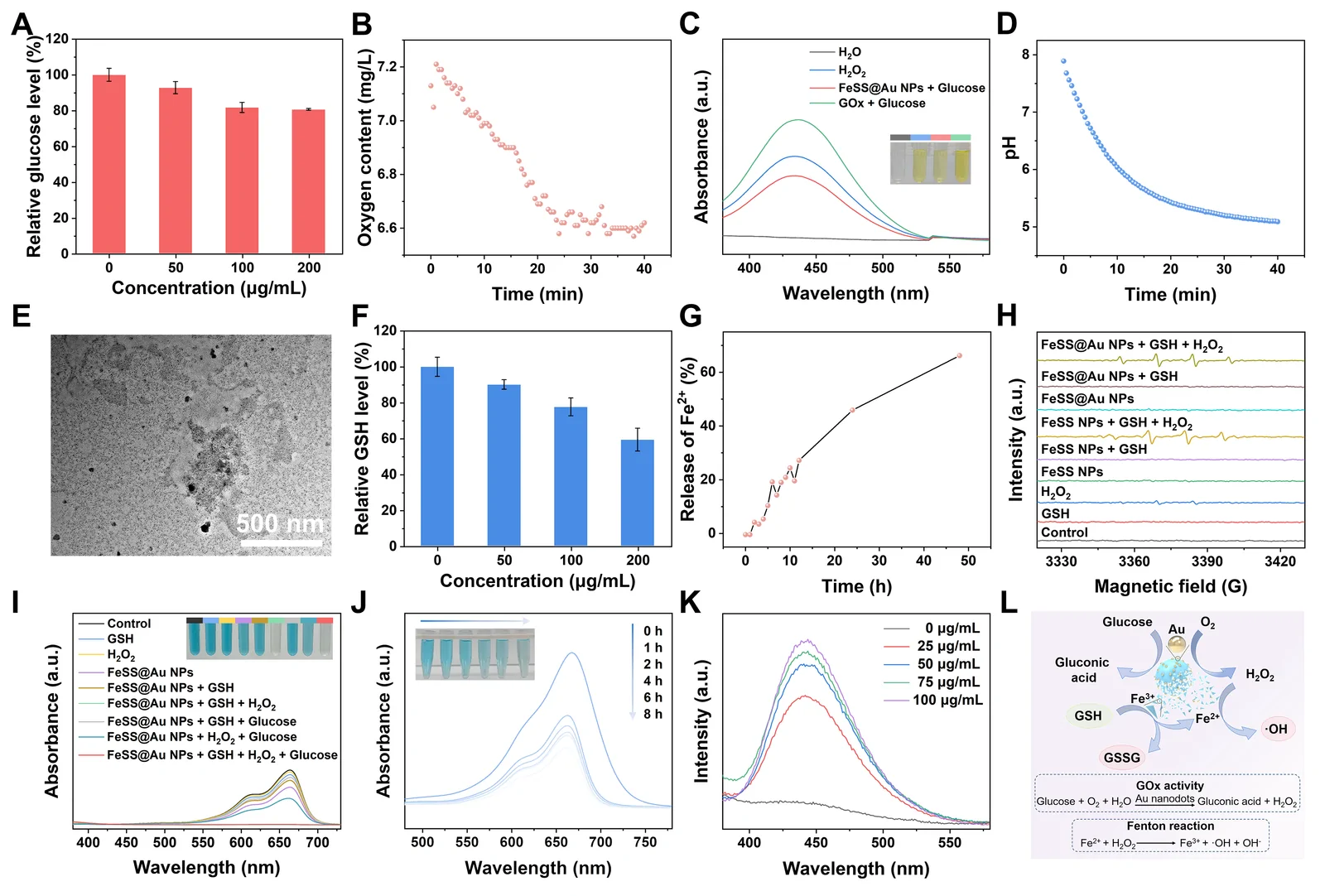
** Properties of FeSS@Au NPs.** (A) The glucose levels *versus* the concentration of FeSS@Au NPs. (B) The oxygen content after treatment with FeSS@Au NPs for different durations. (C) The UV-vis absorption spectra of TMB and HRP after treatment with H_2_O, H_2_O_2_, FeSS@Au NPs + Glucose, and GOx + Glucose. (D) The time-dependent pH after treatment with FeSS@Au NPs. (E) The TEM image of FeSS@Au NPs after incubation in pH 5.5 solution with GSH (2 mM) and H_2_O_2_ (100 μM) for 20 h. (F) The GSH content after incubation with FeSS@Au NPs in different concentrations. (G) The Fe^2+^ release curve of FeSS@Au NPs after treatment in pH 5.5 solution with GSH (2 mM) and H_2_O_2_ (100 μM) from 0 to 48 h. (H) The ESR spectra of DMPO after treatment with different groups. The UV-vis absorption spectra of MB solution after treatment with different groups (I), and FeSS@Au NPs + GSH + H_2_O_2_ + Glucose group with different durations (J). (K) The UV-vis absorption spectra of TA solution after treatment with FeSS@Au NPs in different concentrations. (L) The diagram of GOx-like activity and ·OH-producing ability of FeSS@Au NPs. The data in Figure 2A, F are presented as mean ± SD (n = 3).

**Figure 3:**
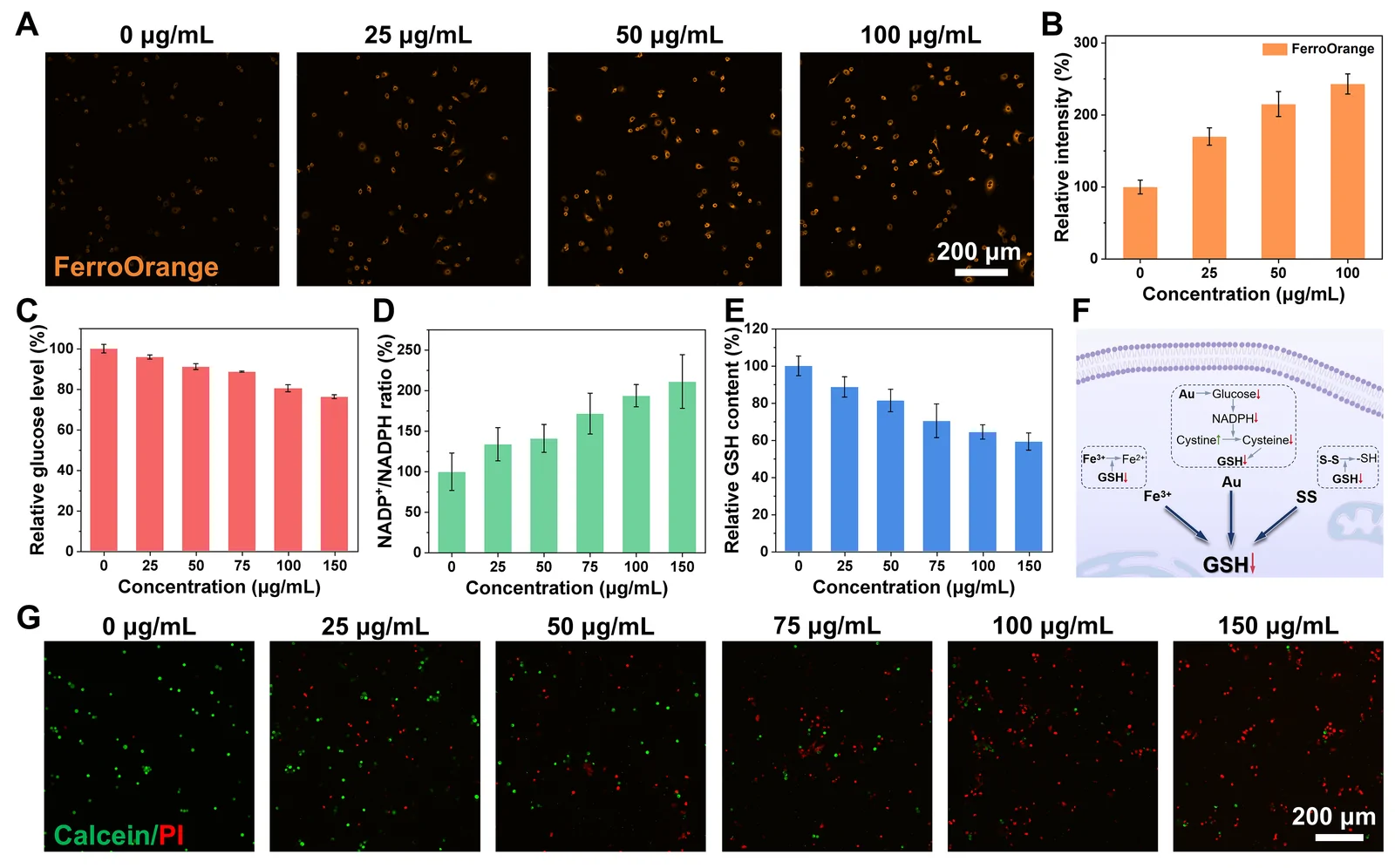
** Cytotoxicity and triple GSH depletion by FeSS@Au NPs.** (A) The CLSM images of A549 cells stained with FerroOrange after incubation with FeSS@Au NPs of different concentrations. (B) The quantitative analysis of the fluorescence intensity in Figure 3A. The glucose level (C), NADP^+^/NADPH ratio (D), and GSH content (E) of A549 cells after incubation with FeSS@Au NPs of different concentrations. (F) The illustration of GSH depletion by FeSS@Au NPs. (G) The CLSM images of A549 cells stained with Calcein/PI cell viability/cytotoxicity assay kit after incubation with FeSS@Au NPs of different concentrations. The data in Figure 3C-E are presented as mean ± SD (n = 3).

**Figure 4:**
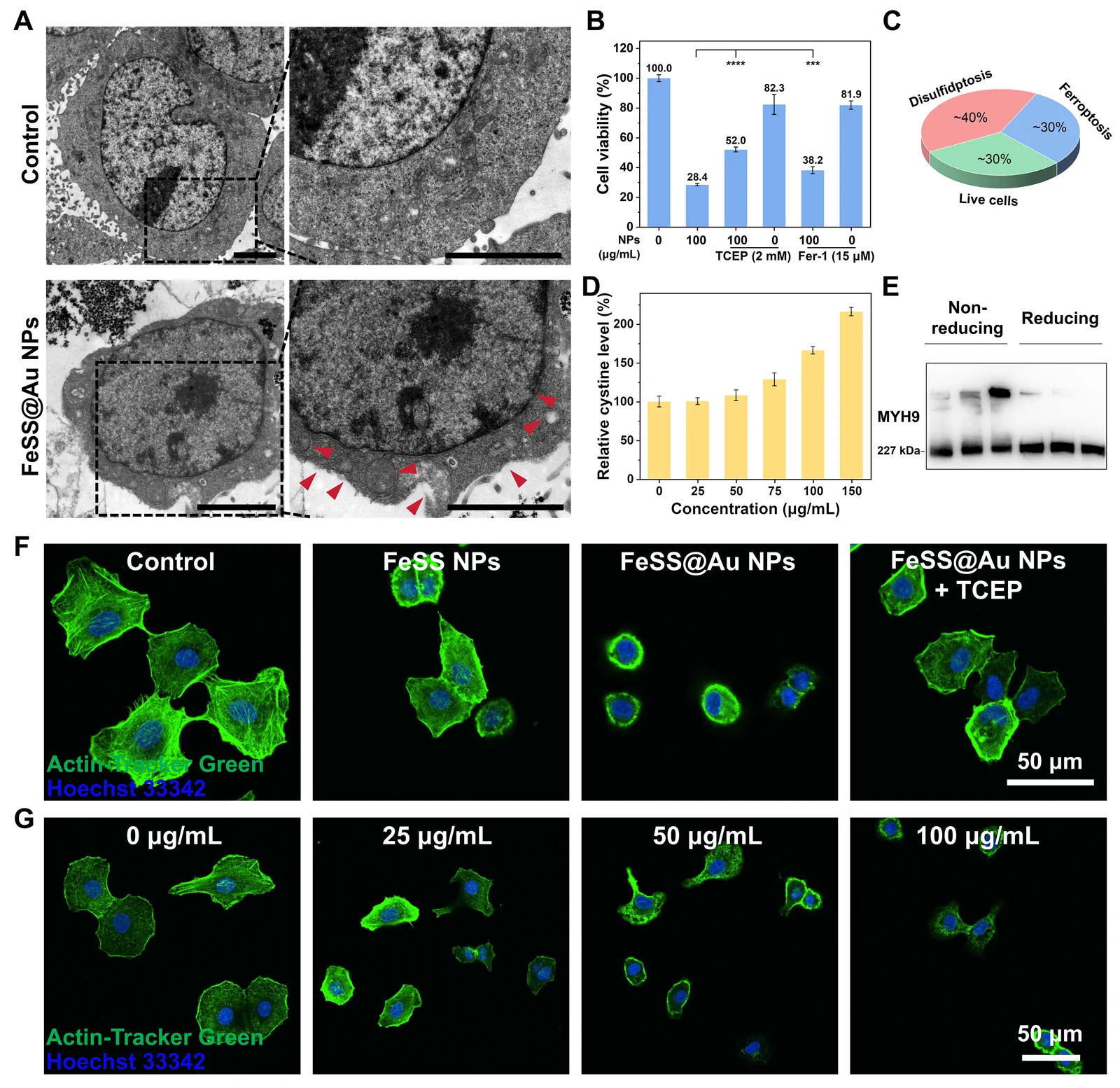
** Disulfidptosis *in vitro*.** (A) The TEM images of A549 cells after incubation without and with FeSS@Au NPs. The red marks refer to the characteristics of ferroptosis. The scale bar is 2 μm. (B) The viability of A549 cells after incubation with FeSS@Au NPs, additional TCEP and Fer-1. (C) The calculated disulfidptosis and ferroptosis ratios. (D) The cystine level of A549 cells after incubation with FeSS@Au NPs of different concentrations. (E) The WB result of MYH9 obtained from A549 cells after incubation with FeSS@Au NPs. The CLSM images of A549 cells stained with Actin-Tracker Green and Hoechst 33342 after incubation with FeSS NPs, FeSS@Au NPs and FeSS@Au NPs + TCEP (F), and FeSS@Au NPs of different concentrations (G). The data in Figure 4B, D are presented as mean ± SD (n = 3). The data in Figure 4B are analyzed by one-way ANOVA test. ***P < 0.001, and ****P < 0.0001.

**Figure 5:**
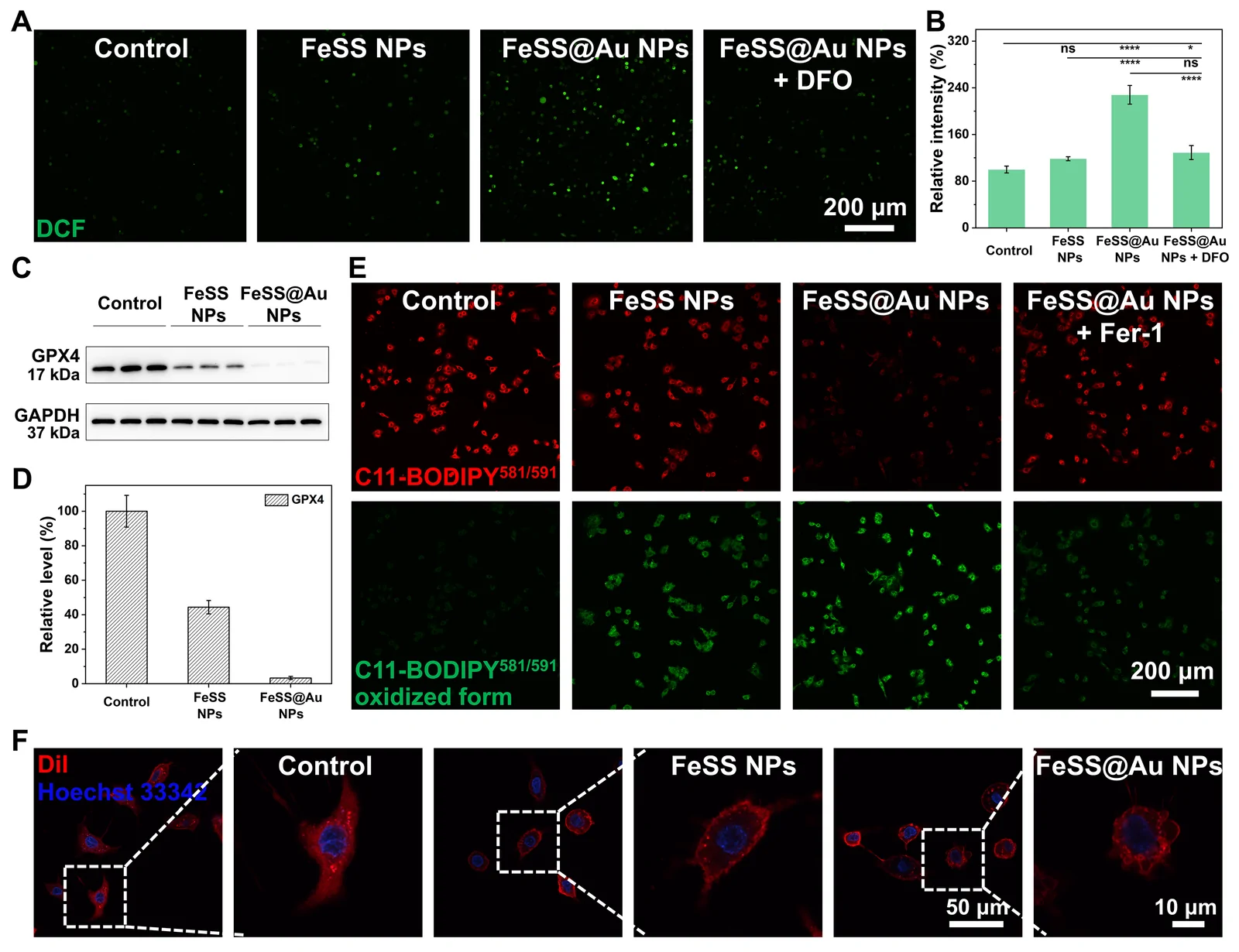
** Ferroptosis *in vitro*.** The CLSM images (A) and quantitative analysis (B) of A549 cells stained with DCFH-DA after incubation with FeSS NPs, FeSS@Au NPs and FeSS@Au NPs + DFO. The WB result of GPX4 (C) and related quantitative analysis (D) of A549 cells after incubation with FeSS NPs and FeSS@Au NPs. (E) The CLSM images of A549 cells stained with C11-BODIPY^581/591^ after incubation with FeSS NPs, FeSS@Au NPs and FeSS@Au NPs + Fer-1. (F) The CLSM images of A549 cells stained with Dil and Hoechst 33342 after incubation with FeSS NPs and FeSS@Au NPs. The data in Figure 5D are presented as mean ± SD (n = 3). The data in Figure 5B are analyzed by one-way ANOVA test. *P < 0.05, and ****P < 0.0001.

**Figure 6:**
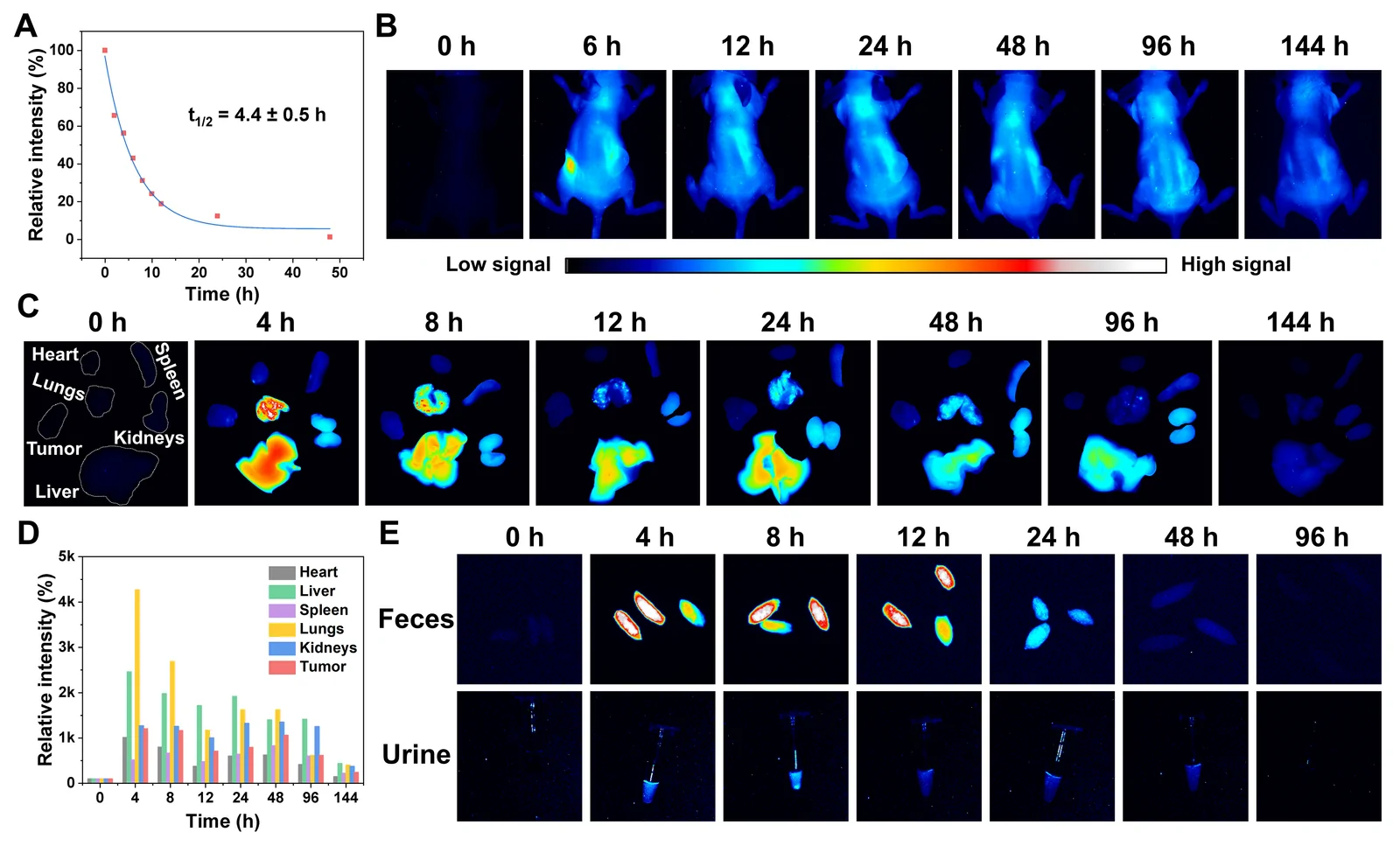
** The pharmacokinetics study *in vivo*.** (A) The relative fluorescence intensity of blood from the mice after administration with ICG-FeSS@Au NPs from 0 to 48 h. The blood circulation half-life is calculated according to the fitted curve. The fluorescence images of body (B) and major organs (C) from 0 to 144 h post-injection. (D) The relative fluorescence intensity of major organs in Figure 6C. (E) The fluorescence images of feces and urine from 0 to 96 h post-injection.

**Figure 7:**
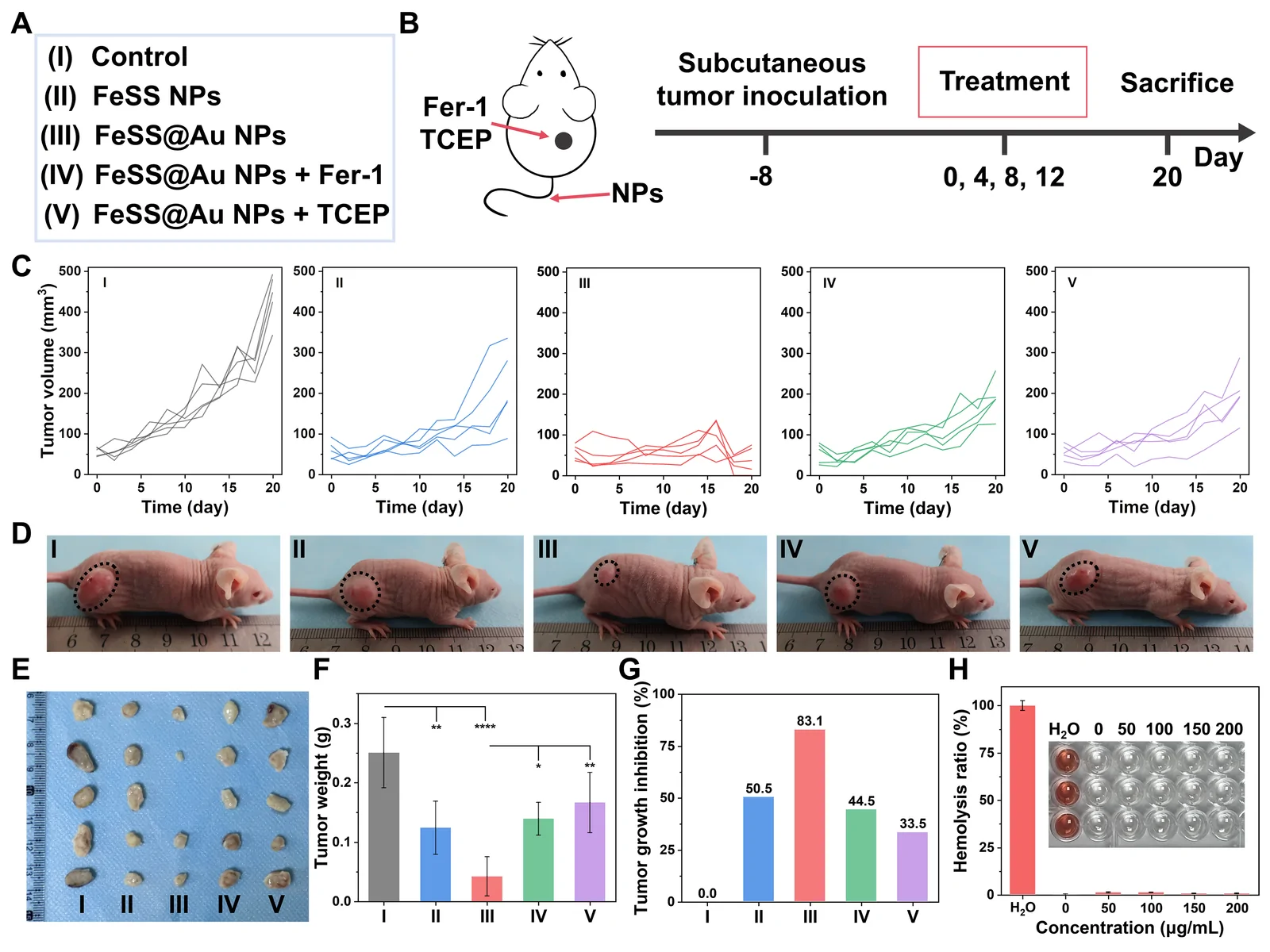
** Therapy *in vivo*.** The groups (A) and the schedule (B) in animal experiments. The tumor volume (C), representative pictures of mice on 20^th^ day (D), pictures of tumors (E), tumor weight (F) and tumor growth inhibition (G) in group I-V. (H) The hemolysis assay of FeSS@Au NPs. The data in Figure 7F, H are presented as mean ± SD (n = 5 in Figure 7F, and n = 3 in Figure 7H). The data in Figure 7F are analyzed by one-way ANOVA test. *P < 0.05, **P < 0.01, and ****P < 0.0001.

## Data Availability

The author can provide all the data used in this work upon reasonable request.

## Abbreviations

A549: human non-small cell lung cancer
ANOVA: analysis of variance
BEAS-2B: normal human lung epithelial
CCK-8: cell counting kit
CLSM: confocal laser scanning microscope
DCFH-DA: 2’,7’-dichlorodihydrofluorescein diacetate
DFO: desferrioxamine
DMPO: 5,5-dimethyl-1-pyrroline N-oxide
EDTA: ethylenediaminetetraacetic acid
EPR: enhanced permeability and retention
ESR: electron spin resonance
Fer-1: ferrostatin-1
FTIR: Fourier transform infrared
GOx: glucose oxidase
GPX4: glutathione peroxidase 4
GSH: reduced glutathione
GSSG: oxidized glutathione
H&E: hematoxylin and eosin
HAuCl_4_·3H_2_O: gold chloride trihydrate
H1299: human non-small cell lung cancer cells
IC50: half maximal inhibitory concentration
ICG: indocyanine green
ICP-AES: inductively coupled plasma atomic emission spectrometry
FeCl_3_·6H_2_O: iron chloride hexahydrate
LPO: lipid peroxide
MB: methylene blue
MYH9: myosin heavy chain 9
NADPH: nicotinamide adenine dinucleotide phosphate
NaBH_4_: sodium borohydride
PBS: phosphate buffered saline
RCD: regulated cell death
ROS: reactive oxygen species
SD: standard deviation
SLC7A11: solute carrier family 7 member 11
SS: dithiodiglycolic acid
TA: disodium terephthalate
TCEP: tris(2-carboxyethyl)phosphine hydrochloride
TEM: transmission electron microscopy
TMB: 3,3’,5,5’-tetramethylbenzidine
TME: tumor microenvironment
UPLC-MS: ultra-performance liquid chromatography-mass spectrometry
UV-vis: ultraviolet-visible
WB: western blotting
XPS: X-ray photoelectron spectroscopy
XRD: X-ray diffraction

## References

[1] Chen D, Guo Z, Yao L, Sun Y, Dian Y, Zhao D (2025). Targeting oxidative stress-mediated regulated cell death as a vulnerability in cancer. Redox Biol.

[2] Zhang R, Li Y, Fu H, Zhao C, Li X, Wang Y (2025). Nanomedicine strategies for cuproptosis: Metabolic reprogramming and tumor immunotherapy. Acta Pharm Sin B.

[3] Liang L, Zhu Z, Jiang X, Tang Y, Li J, Zhang Z (2025). Endoplasmic reticulum-targeted strategies for programmed cell death in cancer therapy: Approaches and prospects. J Control Release.

[4] Meng Y, Chen Q, Zhou Z, Li M (2025). Regulated cell death in cancer: Mechanisms, crosstalk, and opportunities for therapy. Cancer Lett.

[5] Wang Y, Fan H, Chen Q, Song H, Li X, Su B (2026). Mitocytosis-inducing nanoparticles alleviate gemcitabine resistance via dual disruption of pyrimidine synthesis and redox homeostasis in pancreatic ductal adenocarcinoma. Biomaterials.

[6] Qiu L, Li W, Zhang L, Zhang X, Zhao H, Miyagishi M (2025). p52-ZER6/DAZAP1 axis promotes ferroptosis resistance and colorectal cancer progression via regulating SLC7A11 mRNA stabilization. Acta Pharm Sin B.

[7] Yang L, Cai Y, Nurtay N, Pan M, Zhang C, Hu X (2025). Three-in-one: Biomimetic trimetallic ionic-site nanozyme composite as a redox homeostasis disruptor for ferroptosis/cuproptosis/pyroptosis co-activated nanocatalytic therapy. Small.

[8] Liu Y, Cai X, Liu J, Luo Z, Zhang J, Cao Z (2025). The role of redox homeostasis in tumor progression: Implications for cancer therapy. Acta Biomater.

[9] Tao J, Ning W, Shi X, Wu T, Liu S, Wan S (2025). Self-delivery nanomedicine for selective mitochondrial copper depletion and oxidative stress amplification. ACS Nano.

[10] Jiang S, Li W, Zhang Y, Lin J, Huang P (2025). Oxygen-regulated enzymatic nanoplatform for synchronous intervention in glycolysis and oxidative phosphorylation to augment antitumor therapy. J Control Release.

[11] Yu W, Jin D, Zhang Y, Wang S, Yu J, Liu M (2025). Provoking tumor disulfidptosis by single-atom nanozyme via regulating cellular energy supply and reducing power. Nat Commun.

[12] Wang K, Li L, Liang G, Xiao H, Zhang L, Liu T (2025). Sonodynamic activated nanoparticles with Glut1 inhibitor and cystine-containing polymer stimulate disulfidptosis for improved immunotherapy in bladder cancer. Biomaterials.

[13] Gong Y, Chen L, Wang H, Zheng D, Li F, Wu C (2025). ANXA1 promotes intrahepatic cholangiocarcinoma proliferation and growth by regulating glutamine metabolism through GOT1 stabilization. J Exp Clin Cancer Res.

[14] Hou G, Lu Y, Chen Y, Lei H, Cheng L (2025). Recent advances in nanotechnology for metabolic cancer therapy. Coord Chem Rev.

[15] Jin XK, Zhang SK, Zhang SM, Liang JL, Yan X, Lin YT (2025). Disrupting intracellular homeostasis by copper-based nanoinducer with multiple enzyme-mimicking activities to induce disulfidptosis-enhanced pyroptosis for tumor immunotherapy. Adv Mater.

[16] Zhou QM, Lu YF, Yang XY, Zhang JG, Wang YN, Luo WP (2025). Redox-driven hybrid nanoenzyme dynamically activating ferroptosis and disulfidptosis for hepatocellular carcinoma theranostics. J Colloid Interface Sci.

[17] Liu X, Nie L, Zhang Y, Yan Y, Wang C, Colic M (2023). Actin cytoskeleton vulnerability to disulfide stress mediates disulfidptosis. Nat Cell Biol.

[18] Zhang M, Zheng H, Zhu X, Liu S, Jin H, Chen Y (2025). Synchronously evoking disulfidptosis and ferroptosis via systematical glucose deprivation targeting SLC7A11/GSH/GPX4 antioxidant axis. ACS Nano.

[19] Zhou S, Liu J, Wan A, Zhang Y, Qi X (2024). Epigenetic regulation of diverse cell death modalities in cancer: A focus on pyroptosis, ferroptosis, cuproptosis, and disulfidptosis. J Hematol Oncol.

[20] Yan Y, Teng H, Hang Q, Kondiparthi L, Lei G, Horbath A (2023). SLC7A11 expression level dictates differential responses to oxidative stress in cancer cells. Nat Commun.

[21] Lin HC, Shen WY, Wei JH, Chen LL, Jia CP, Mo ZY (2025). Rhodium(III)-nitroxyl radical complex triggers dual-pronged disulfidptosis-apoptosis in hepatocellular carcinoma via metabolic sabotage and redox catalysis. Angew Chem Int Ed.

[22] Chen X, Li J, Kang R, Klionsky DJ, Tang D (2021). Ferroptosis: Machinery and regulation. Autophagy.

[23] Yu W, Chen Z, Wu B, Zhang C, Han Y, Zou D (2026). Modular nanotherapeutics with spatiotemporal precision for phase-specific treatment of intracerebral hemorrhage. Bioact Mater.

[24] Yang X, Luo W, Wang Y, Lu Y, Yu J, Liu C (2026). Spatiotemporally controlled ferritin-targeting protac nanoplatform for visualized manipulation of endogenous “iron pool” in pancreatic cancer therapy. Mater Today Bio.

[25] Zhong YL, Wang BB, Chu KF, Wang AJ, Zhao T, Feng JJ (2026). High-entropy intermetallic alloy/carbon nanoflower cascade nanozyme for multi-modal synergistic cancer therapy. J Colloid Interface Sci.

[26] Li J, Wang Y, Li H, Yuan Z, Wei Q, Yang L (2026). Self-amplifying tumor-targeting nanoreactor for enhanced immuno-ferroptosis therapy in triple-negative breast cancer. Chem Eng J.

[27] Bersuker K, Hendricks JM, Li Z, Magtanong L, Ford B, Tang PH (2019). The CoQ oxidoreductase FSP1 acts parallel to GPX4 to inhibit ferroptosis. Nature.

[28] Wang Z, Wang Y, Shen N, Liu Y, Xu X, Zhu R (2024). AMPKα1-mediated ZDHHC8 phosphorylation promotes the palmitoylation of SLC7A11 to facilitate ferroptosis resistance in glioblastoma. Cancer Lett.

[29] Zhang H, Ma J, Hou C, Luo X, Zhu S, Peng Y (2025). A ROS-mediated oxidation-O-GlcNAcylation cascade governs ferroptosis. Nat Cell Biol.

[30] Wang M, Chen Q, Cheng Z, Dai Z, Wu T, Han B (2026). A responsive hydrogel containing bimetallic MOF enabling amplified ferroptosis for cancer therapy via dual cellular homeostasis disruption. Adv Funct Mater.

[31] Qin Y, Fan J, Zhang Y, Yang W, Cao J, Liu B (2025). Natural polyphenol-metal supramolecular nanocomplex for ferroptosis activation in chemoresistant hepatocellular carcinoma. Aggregate.

[32] Li Y, Han S, Zhao Y, Yan J, Luo K, Li F (2025). A redox-triggered polymeric nanoparticle for disrupting redox homeostasis and enhanced ferroptosis. Small.

[33] Li Q, Wu Z, Chen S, Liang Y, Zhu K, Su N (2025). Enhancing ferroptosis-mediated radiosensitization via synergistic disulfidptosis induction. ACS Nano.

[34] Liu Y, Chen H, Chen K, Shang Q, Lin F, Zhang P (2025). Luteolin alleviates estrogen deficiency-induced muscle atrophy via targeting SLC7A11-mediated ferroptosis. Phytomedicine.

[35] Liu J, Gong Y, Wang D, Kang S, Gong S, Ma H (2025). Water-dispersible mxene governs glycolysis for cancer synergistic therapy. Small.

[36] Wang Z, Li A, Song Z, Liu X, Ge Y, Chen Z (2025). CypA mediates non-small cell lung cancer chemoresistance by attenuating ferroptosis via stabilizing SLC7A11. Adv Sci.

[37] Xu K, Li K, He Y, Mao Y, Li X, Zhang L (2025). Engineered nanoplatform mediated gas therapy enhanced ferroptosis for tumor therapy in vivo. Bioact Mater.

[38] Zhang NY, Zhuan W, Cai MZ, Zhou KT, Li HZ, Liu YX (2025). De novo design of hexamer-linkerdual-action nano-PROTAC for tumor-specific ferroptosis. Angew Chem Int Ed.

[39] Li Y, Lu Y, Lu W, Zhong N, Li N, Xu Z (2025). Transformable albumin-based nanocapsules selectively amplify tumor starvation and disulfidptosis through metabolic deception. J Control Release.

[40] Cao Y, Zhao X, Miao Y, Liu X, Liu X, Yue Z (2025). Bilayer self-assembly encapsulated by engineered vesicle disrupts glutathione to induce disulfidptosis-enhanced cuproptosis for tumor immunotherapy. J Control Release.

[41] Zhang G, Duan J, Yu F, Qi Y, Zhao S, Zhao Y (2025). GSH-responsive Mn2+ burst nanoboxes as mitophagy intervention agents augment ferroptosis and chemoimmunotherapy in triple-negative breast cancer. Adv Funct Mater.

[42] Chen H, Liu R, Fu J, Zhang X, Zhu X, Wei M (2025). Surface engineering of copper peroxide nanodots and Au nanozyme on porphyrin-based metal-organic frameworks induces cascade reactions for colorectal cancer treatment. Chem Eng J.

[43] Qu J, Li C, Xu J, Liu S, Wang Q, Tang Z (2025). Natural tannin and upconversion photons co-potentiate Fe Fenton anticancer therapy. Adv Funct Mater.

[44] Rong D, Gao L, Chen Y, Gao X, Tang M, Tang H (2025). Suppression of the LKB1-AMPK-SLC7A11-GSH signaling pathway sensitizes NSCLC to albumin-bound paclitaxel via oxidative stress. Redox Biol.

[45] Li G, Wang Z, Guo Y, Ni C, Gao Y, Xu K (2025). Copper-doped layered double hydroxides co-deliver proteins/drugs for cascaded chemodynamic/immunotherapy via dual regulation of tumor metabolism. Acta Biomater.

