# Supplementary material for: Triple glutathione depletion-augmented disulfide and lipid peroxide accumulation to potentiate disulfidptosis and ferroptosis
Source: Theranostics. 2026 Sep 9;16(16):9185–99. doi: 10.7150/thno.137256 (PMC13684391; doi:10.7150/thno.137256)
Supplement: Supplementary file 1 [file thnov16p9185s1.pdf]

Supplementary Material

Triple glutathione depletion-augmented disulfide and lipid peroxide accumulation to potentiate disulfidptosis and ferroptosis

Shulin Du[1,2,#], Moyi Zhang[2,#], Hao Zheng[2], Ran Gao[2], Jinrong Chen[2], Rouyu Lin[2], Jing Li[3], Mengsi Zhang[1,2,] and Hao Zhang[1,2,*]*

[1]Joint Laboratory of Opto-Functional Theranostics in Medicine and Chemistry, Institute of Translational Medicine, The First Hospital of Jilin University, Changchun 130021, P. R. China.

[2]State Key Laboratory of Supramolecular Structure and Materials, College of Chemistry, Jilin University, Changchun 130012, P. R. China.

[3]Scientific Research Center, China-Japan Union Hospital of Jilin University, Changchun 130033, P. R. China.

[#]These authors contributed equally to this work.

*Address correspondence to zhang_ms@jlu.edu.cn; hao_zhang@jlu.edu.cn.

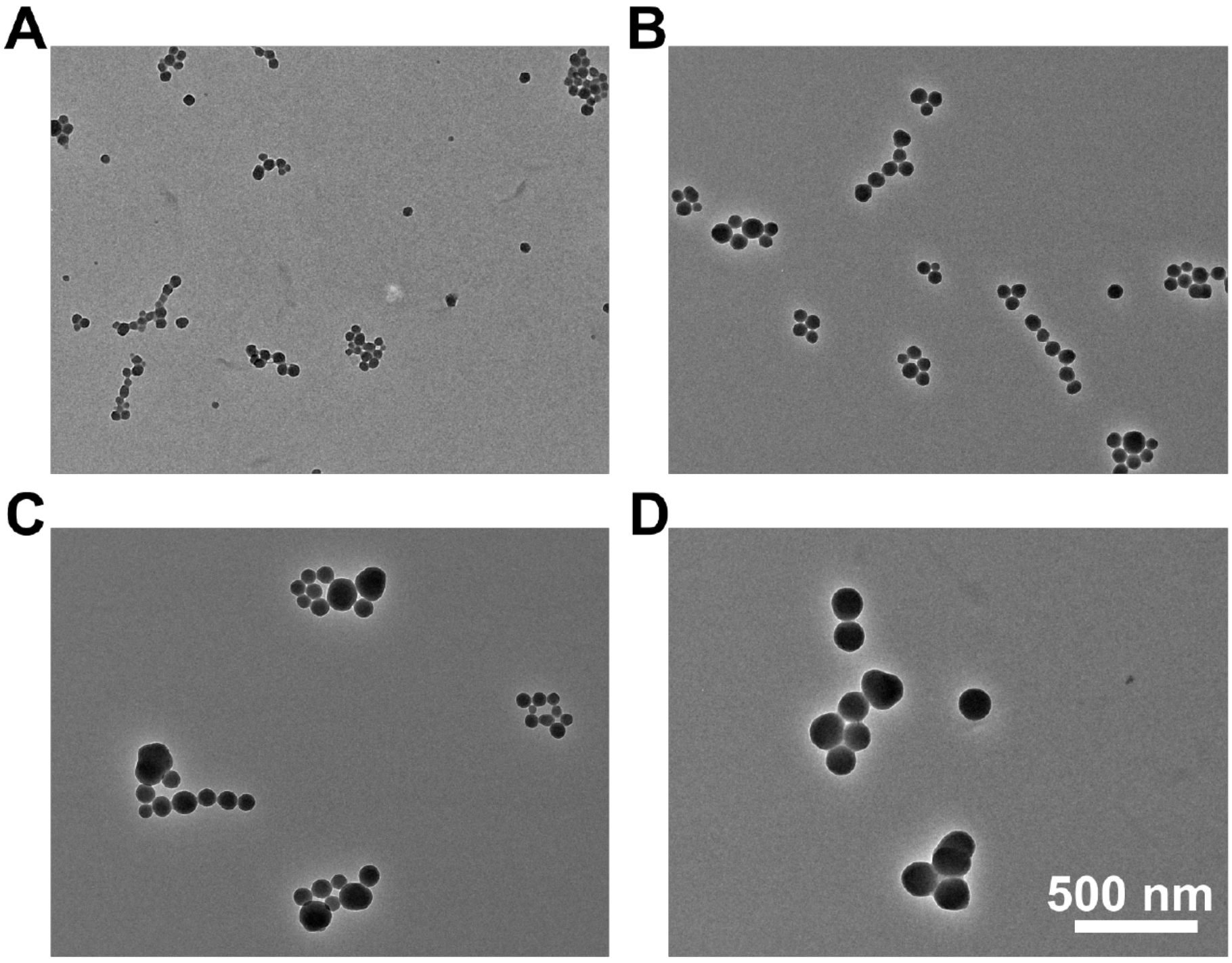

Figure S1. TEM images of FeSS NPs prepared by altering SS feeding amounts from 0.2 (A) to 0.5 (B), 0.7 (C), and 1.0 mL (D), while the $FeCl_3$ amount is fixed at 0.5 mL. The concentrations of SS and $FeCl_3$ are 13.5 and 12.5 mg/mL, respectively.

Table S1. Summary of the diameters of FeSS NPs prepared by altering SS feeding amounts and a fixed $FeCl_3$ amount. The concentrations of SS and $FeCl_3$ are 13.5 and 12.5 mg/mL, respectively.

Group	SS (mL)	H_2O (mL)	$FeCl_3$ (mL)	Diameter (nm)
Figure S1A	0.2	9.3	0.5	~40
Figure S1B	0.5	9.0	0.5	~60
Figure S1C	0.7	8.8	0.5	~90
Figure S1D	1.0	8.5	0.5	~130

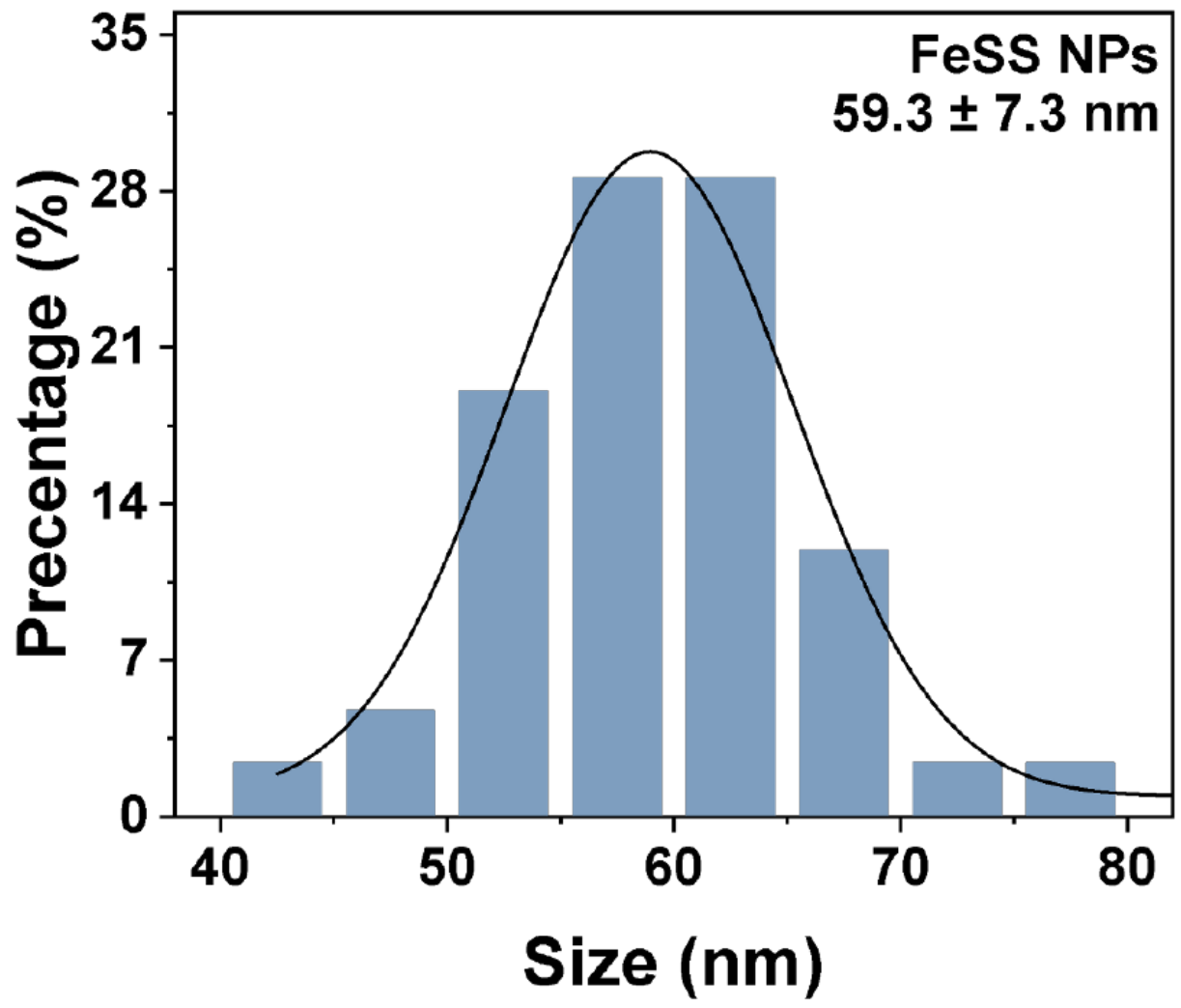

Figure S2. TEM size distribution of FeSS NPs in Figure 1A. The FeSS NPs are prepared with 0.5 mL SS and 0.5 mL $FeCl_3$, referring to Table S1.

Table S2. The component analysis of FeSS NPs by ICP and elemental analyser.

	Element	Elemental mass fraction (%)	Component	Component mass fraction (%)	Mass/Molecular weight	Molar ratio
FeSS NPs	Fe	22.13	Fe	22.1	22.1/55.8 = 0.40	Fe:SS ≈ 1:1
	S	23.83	SS	67.8	67.8/182.2 = 0.37	
	C	19.29		73.2	73.2/182.2 = 0.40	
	H	2.54		77.1	77.1/182.2 = 0.42	

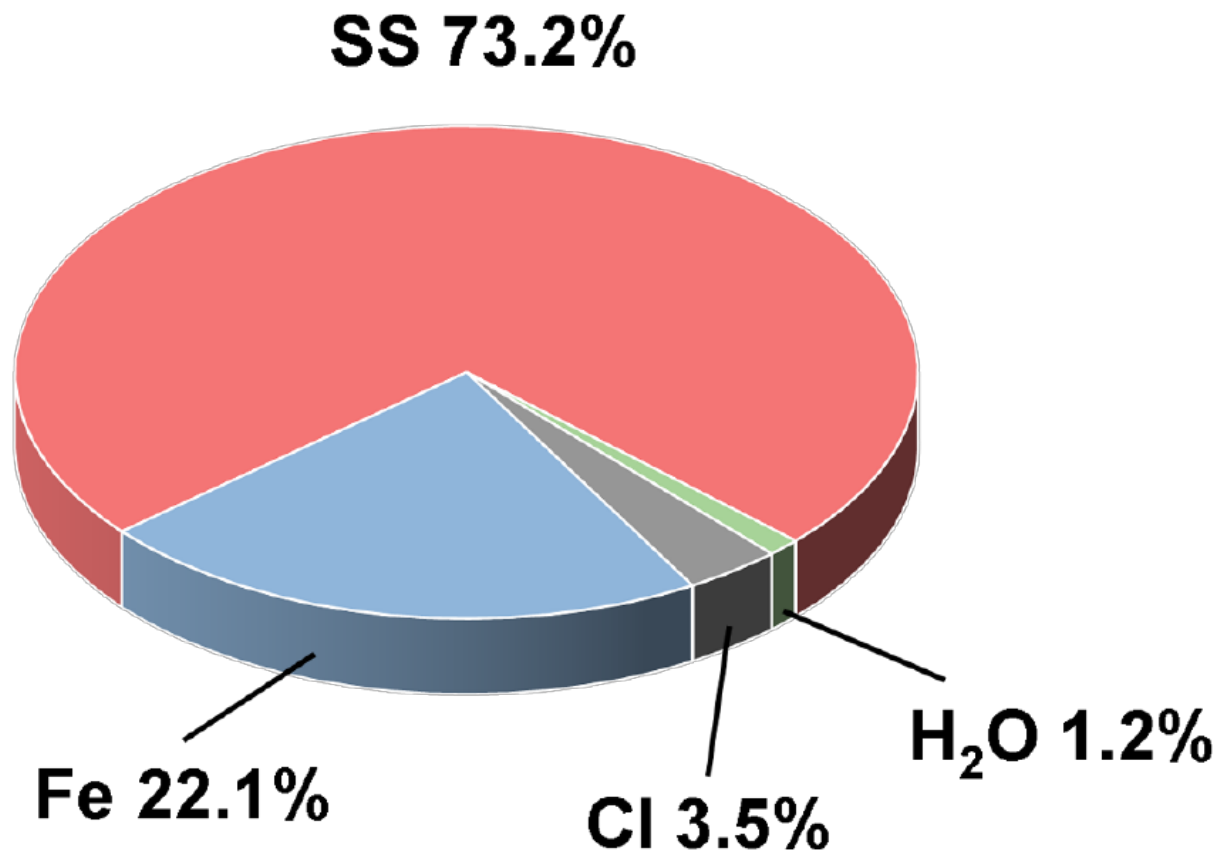

Figure S3. The ratios of different components in FeSS NPs. The SS mass fraction is calculated according to the C element mass fraction, referring to Table S2.

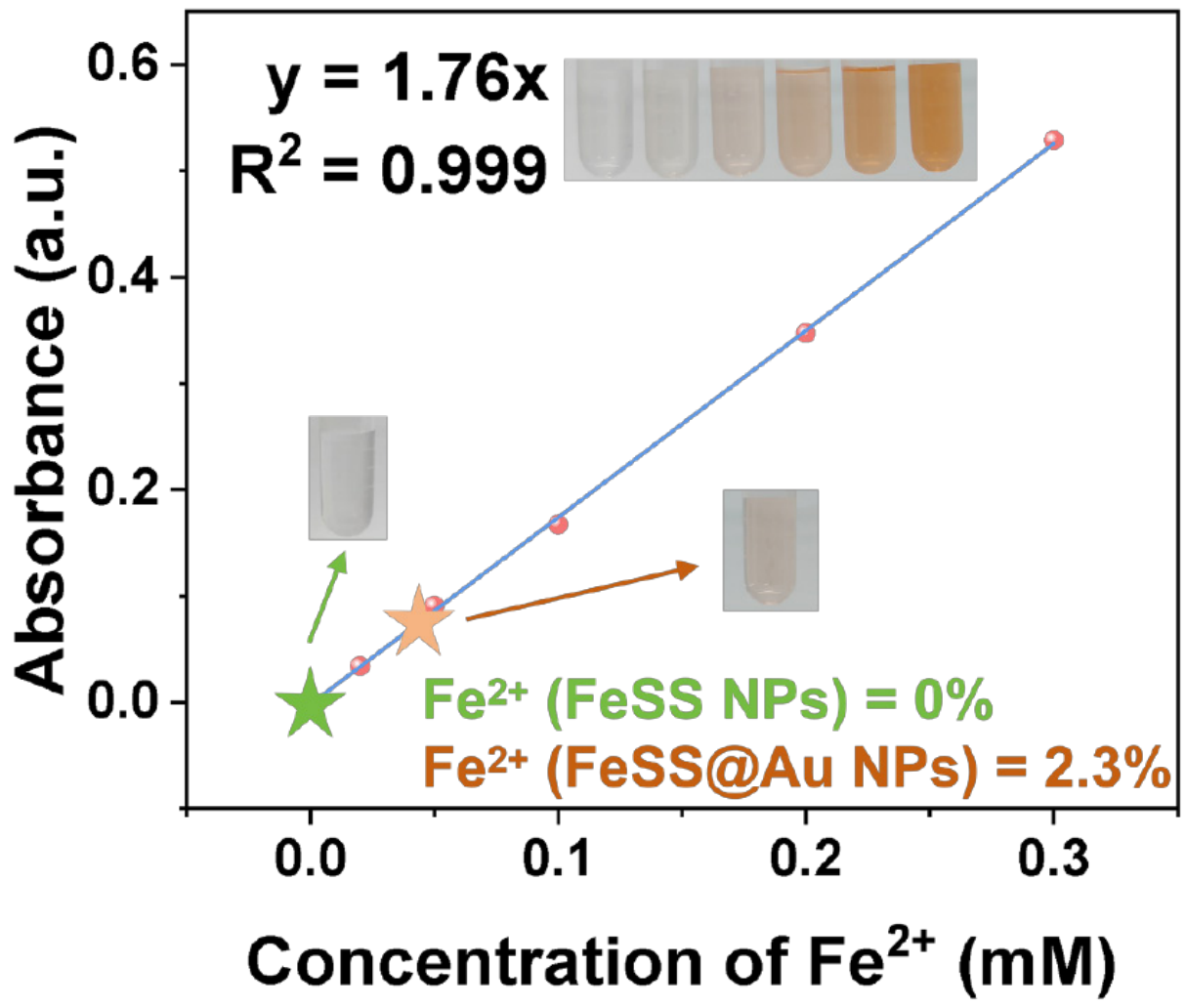

Figure S4. The standard curve and images of 1,10-phenanthroline colorimetric assay for Fe^{2+}. The stars represent the Fe^{2+} concentrations, which are used for calculating the Fe^{2+} contents in FeSS NPs and FeSS@Au NPs.

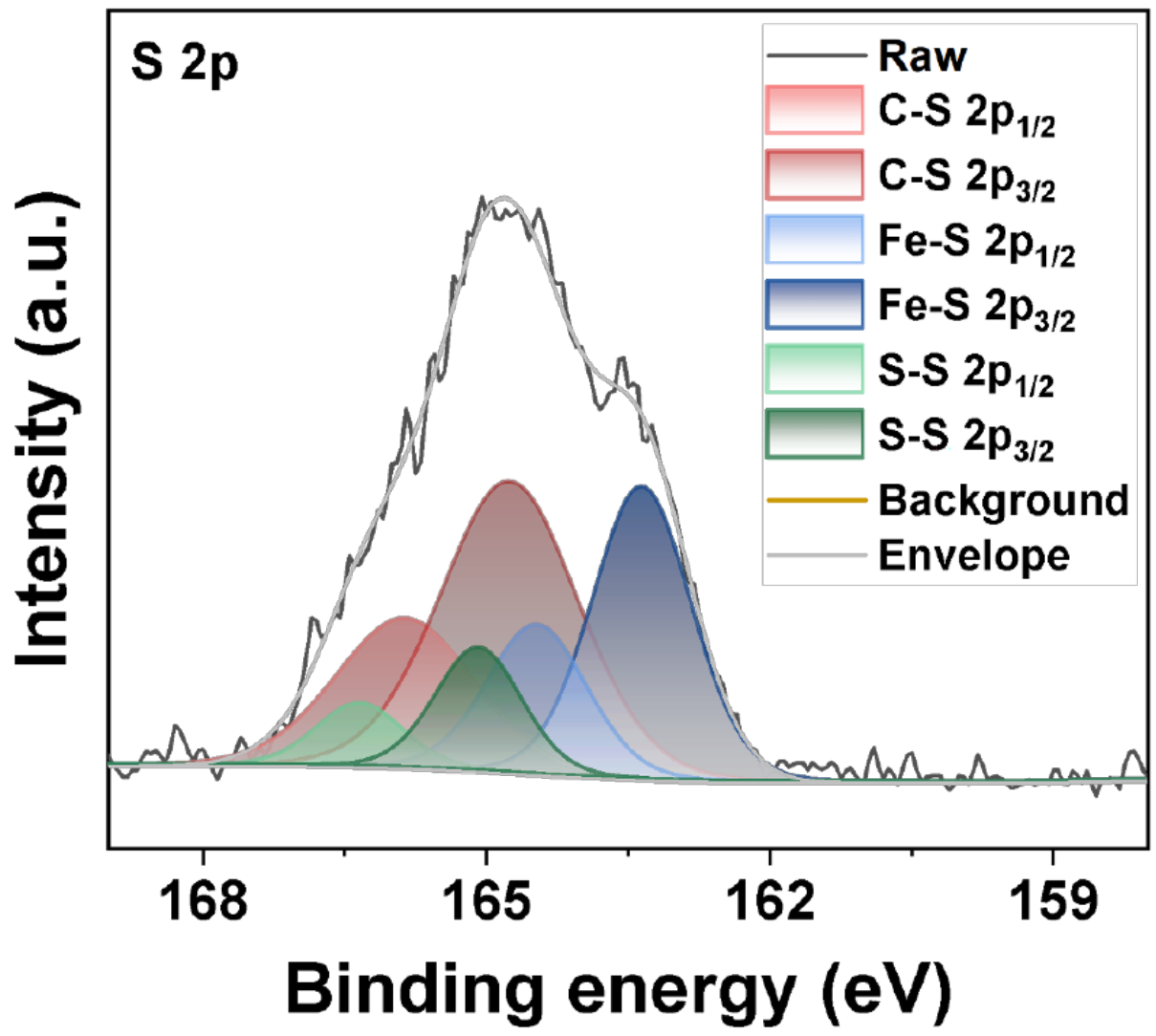

Figure S5. The high-resolution XPS S 2p spectrum of FeSS NPs.

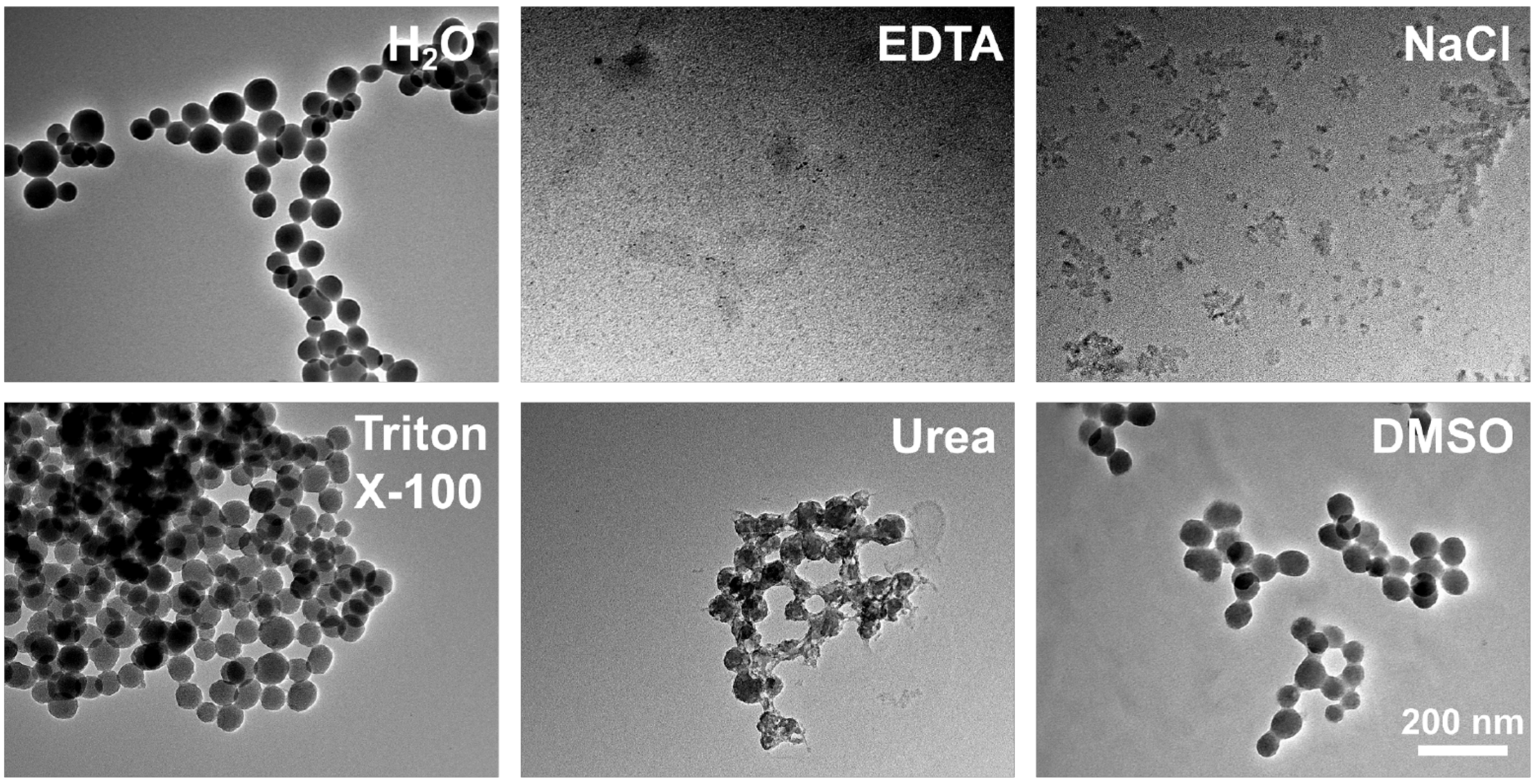

Figure S6. TEM images of FeSS NPs after incubation in different solutions for 15 h.

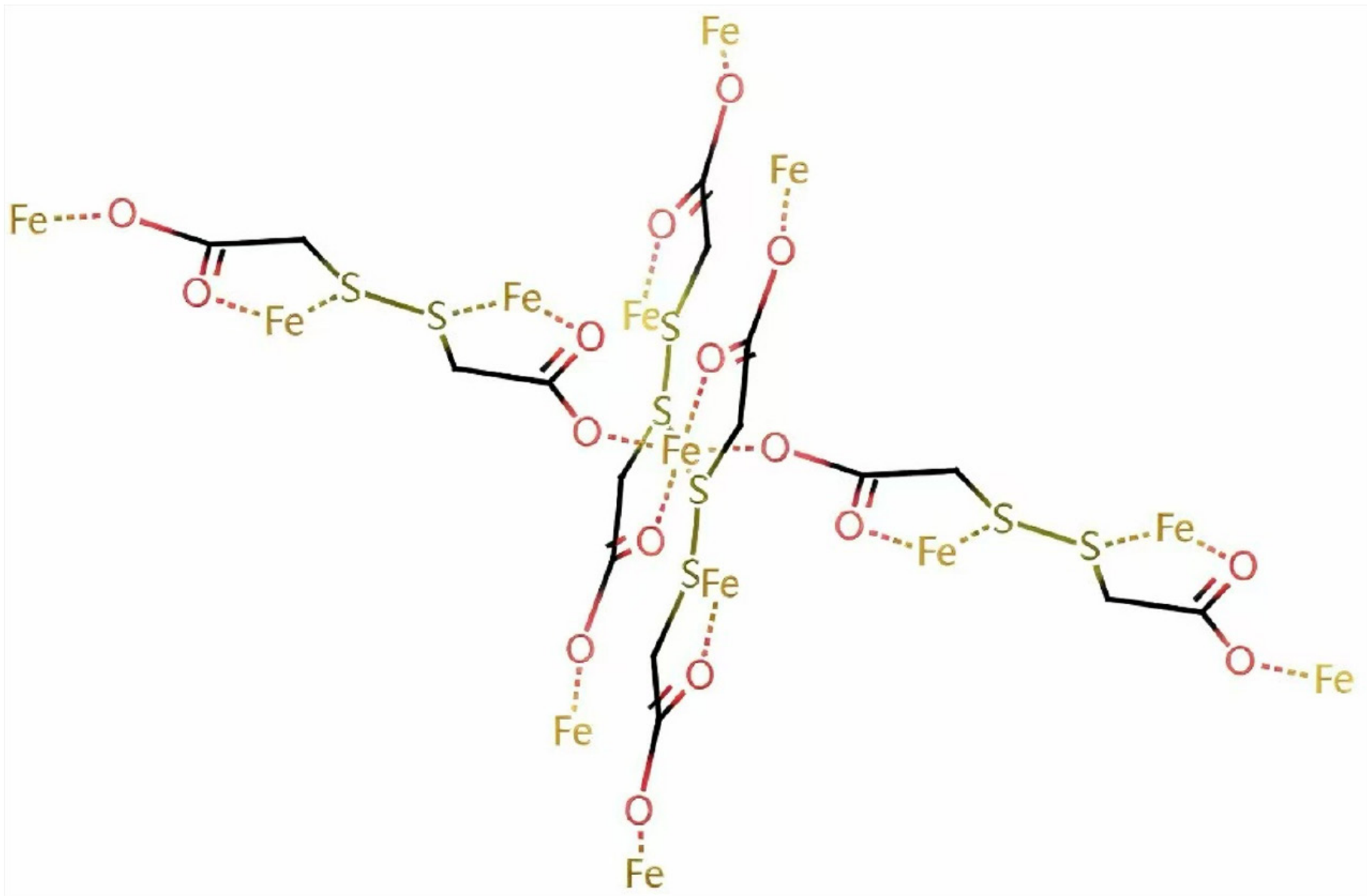

Figure S7. The proposed structure of FeSS NPs. One iron ion coordinates with two O atoms in C=O, two O atoms in -OH and two S atoms in S-S of SS. The ratio of Fe-to-SS is 1:1.

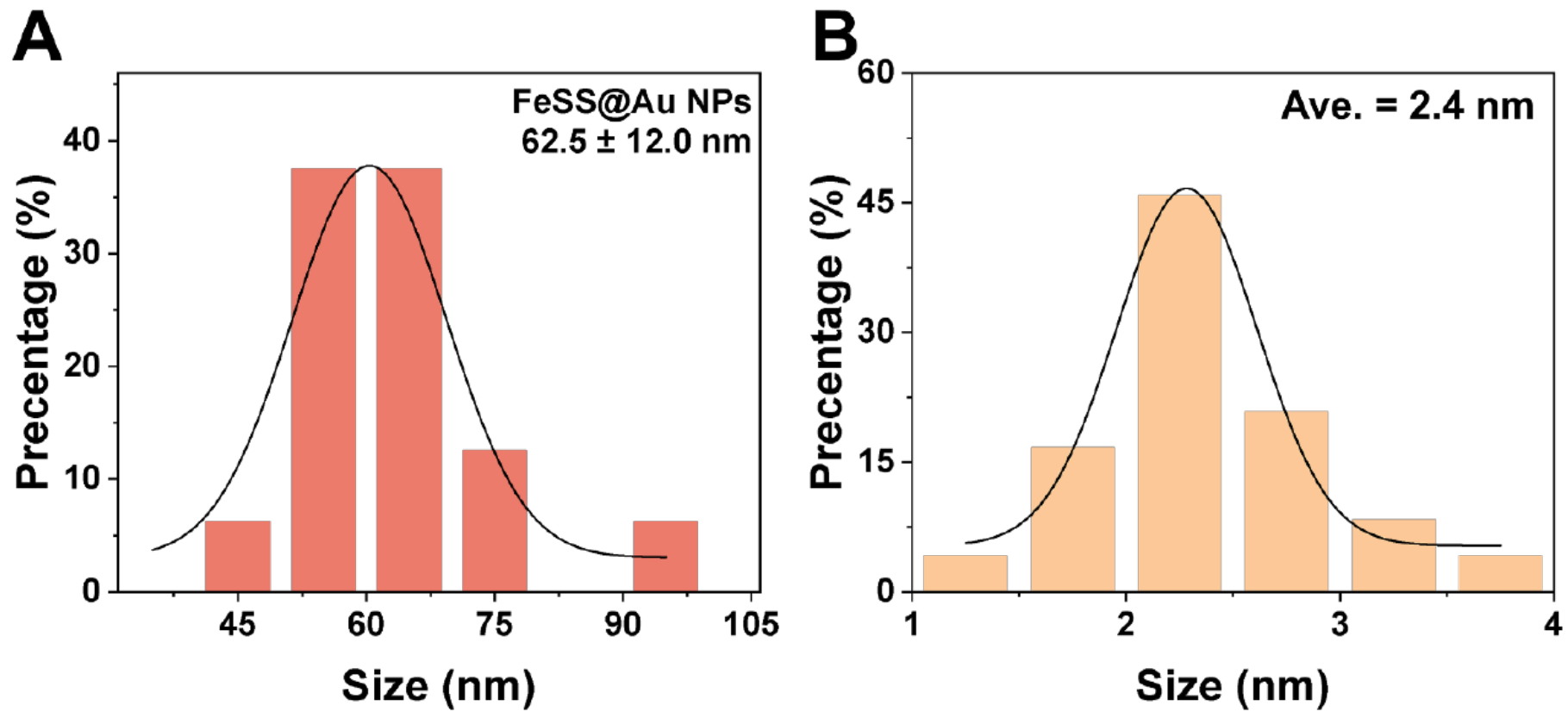

Figure S8. TEM size distributions of FeSS@Au NPs in Figure 1D (A) and the Au nanodots on FeSS@Au NPs in Figure 1E (B).

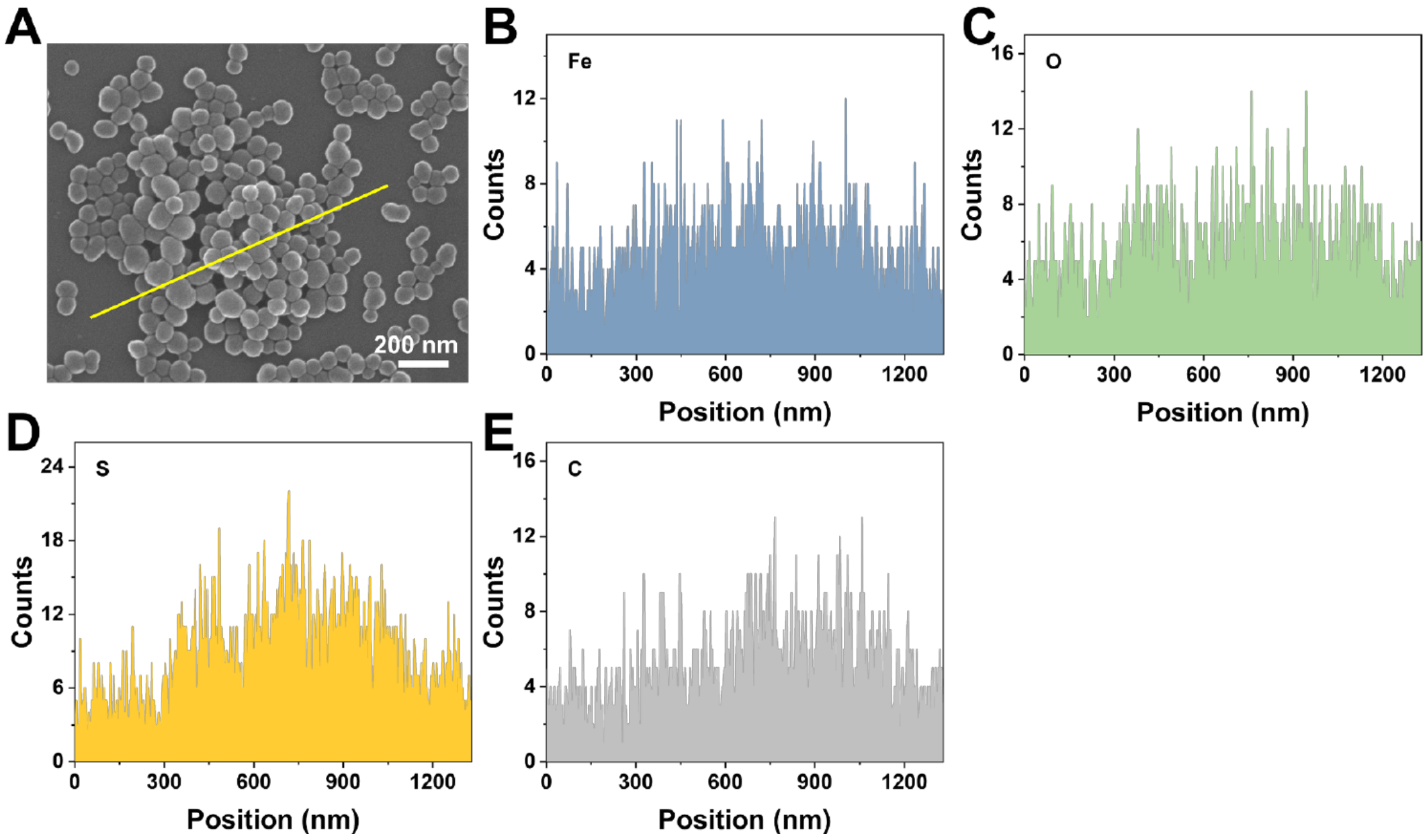

Figure S9. TEM image (A) and elemental line-scan mapping images (B-E) of FeSS NPs.

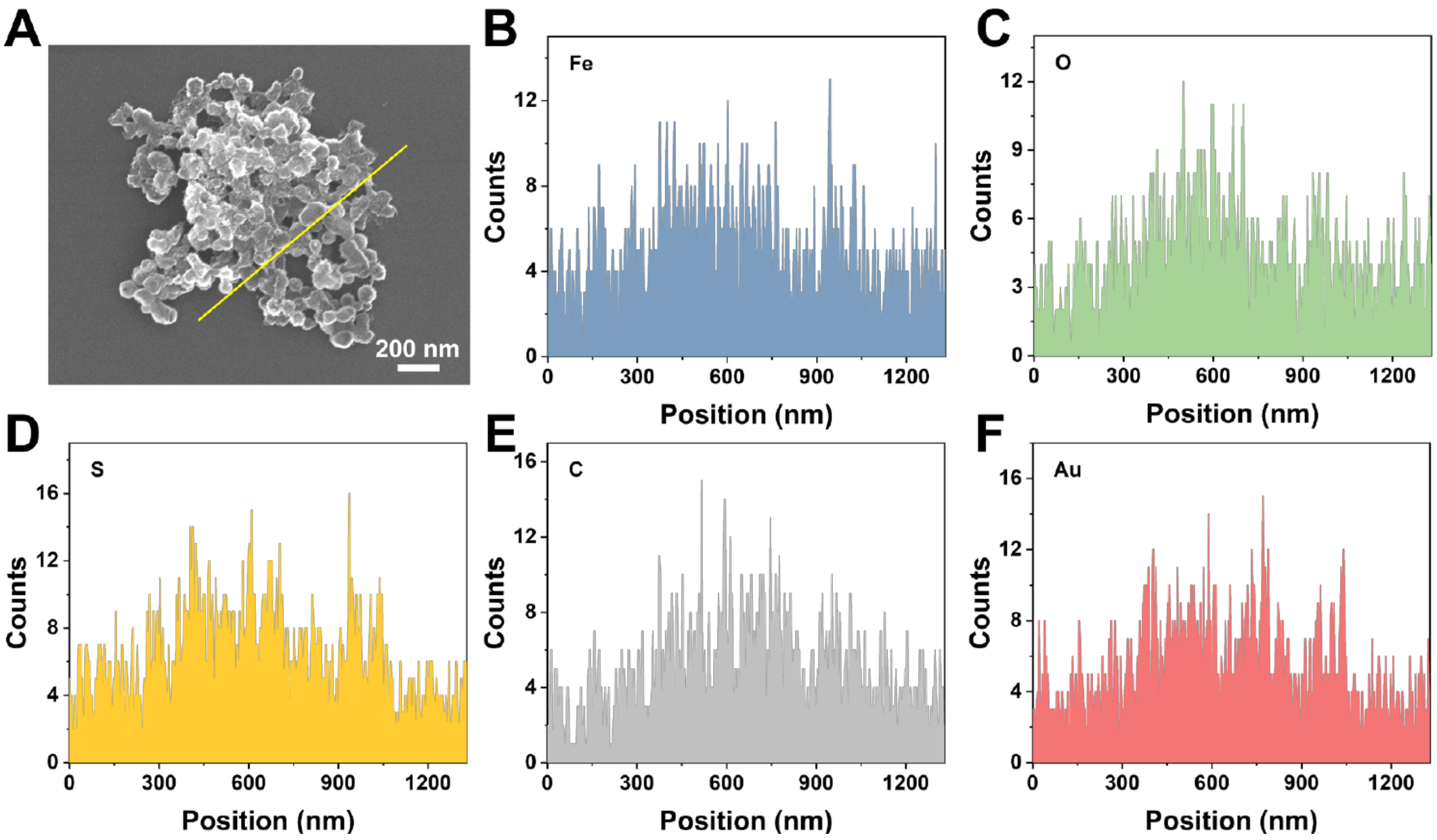

Figure S10. TEM image (A) and elemental line-scan mapping images (B-F) of FeSS@Au NPs.

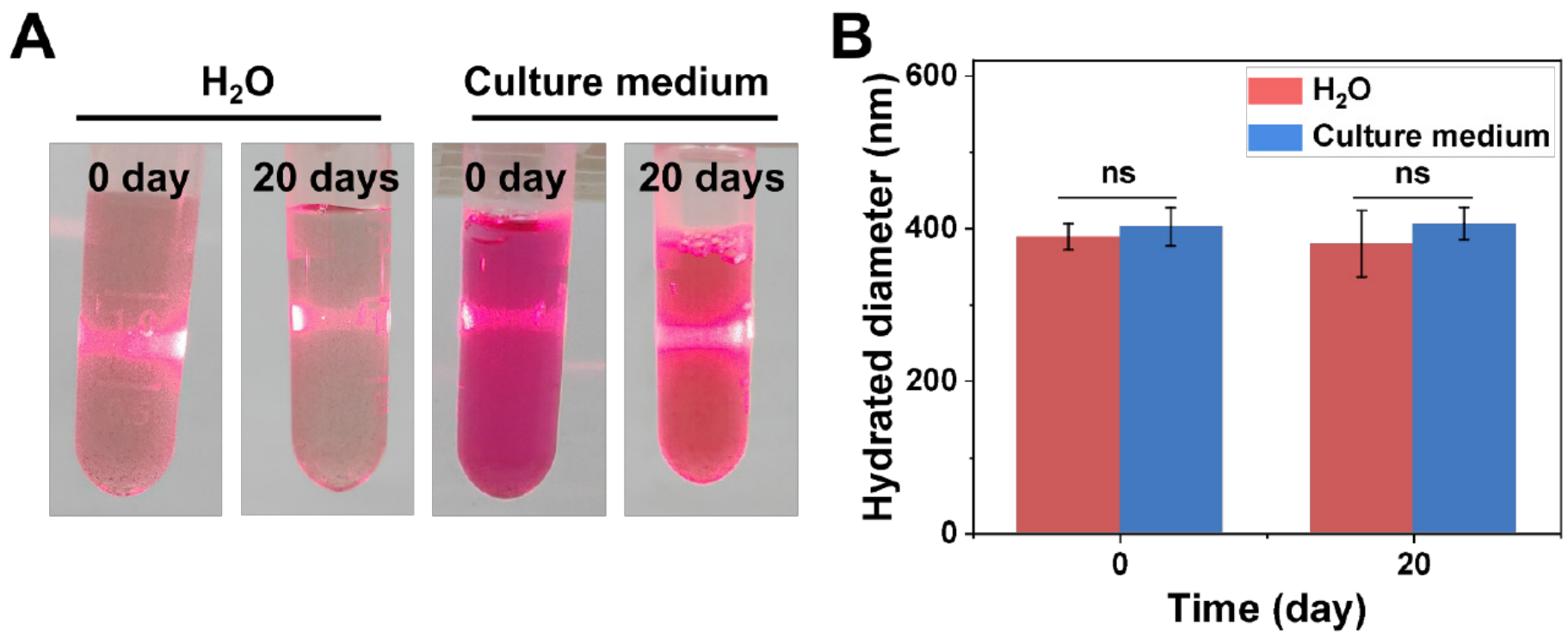

Figure S11. The pictures of Tyndall effect (A) and the hydrated diameters (B) of FeSS@Au NPs incubated in H_2O and culture medium for 0 and 20 days. The data in Figure S11B are presented as mean ± SD (n = 3).

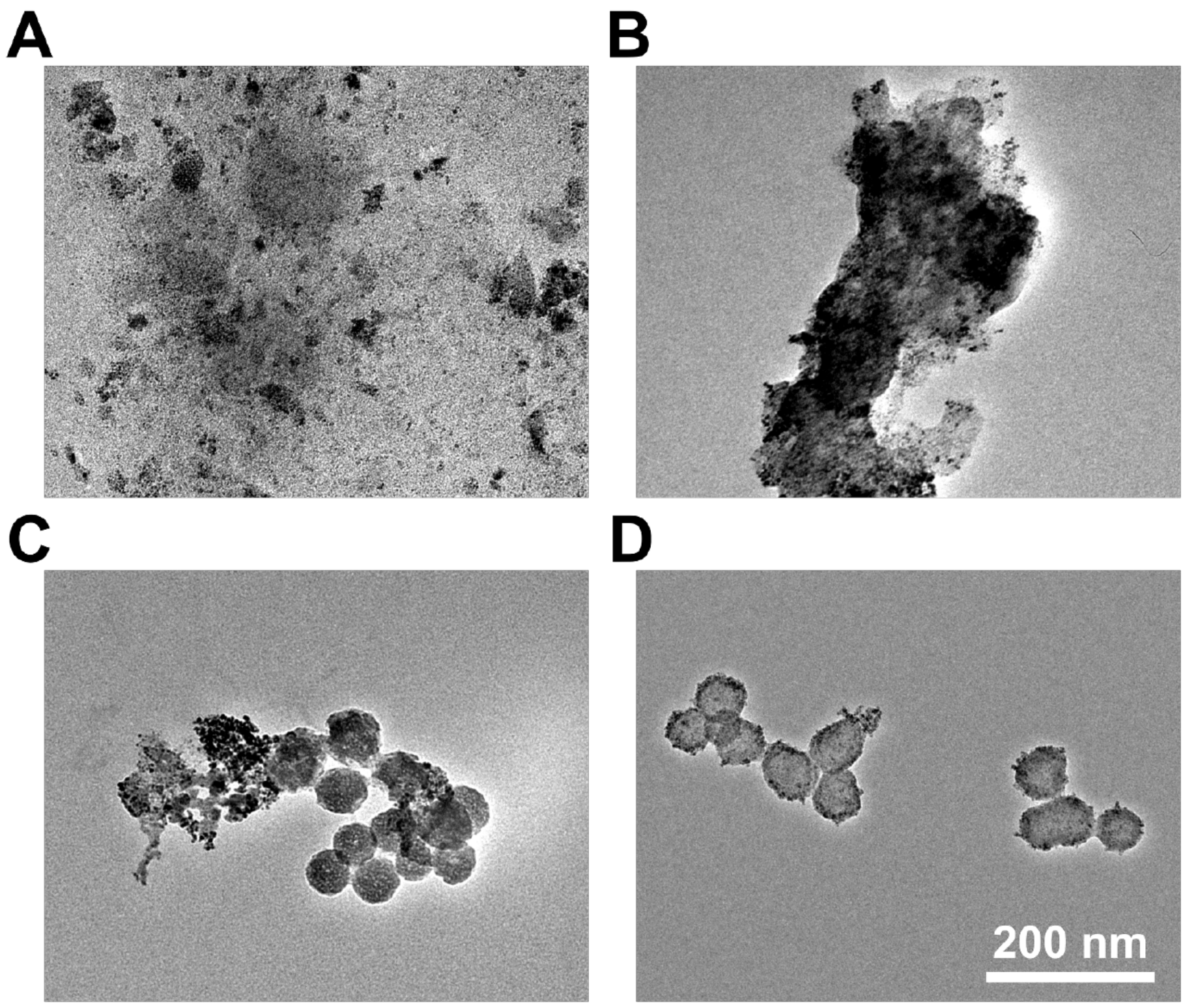

Figure S12. TEM images of FeSS@Au NPs after incubation in GSH (2 mM) solution (A), H_2O_2 (100 μM) solution (B), pH 5.5 aqueous solution (C), and pH 7.4 aqueous solution (D).

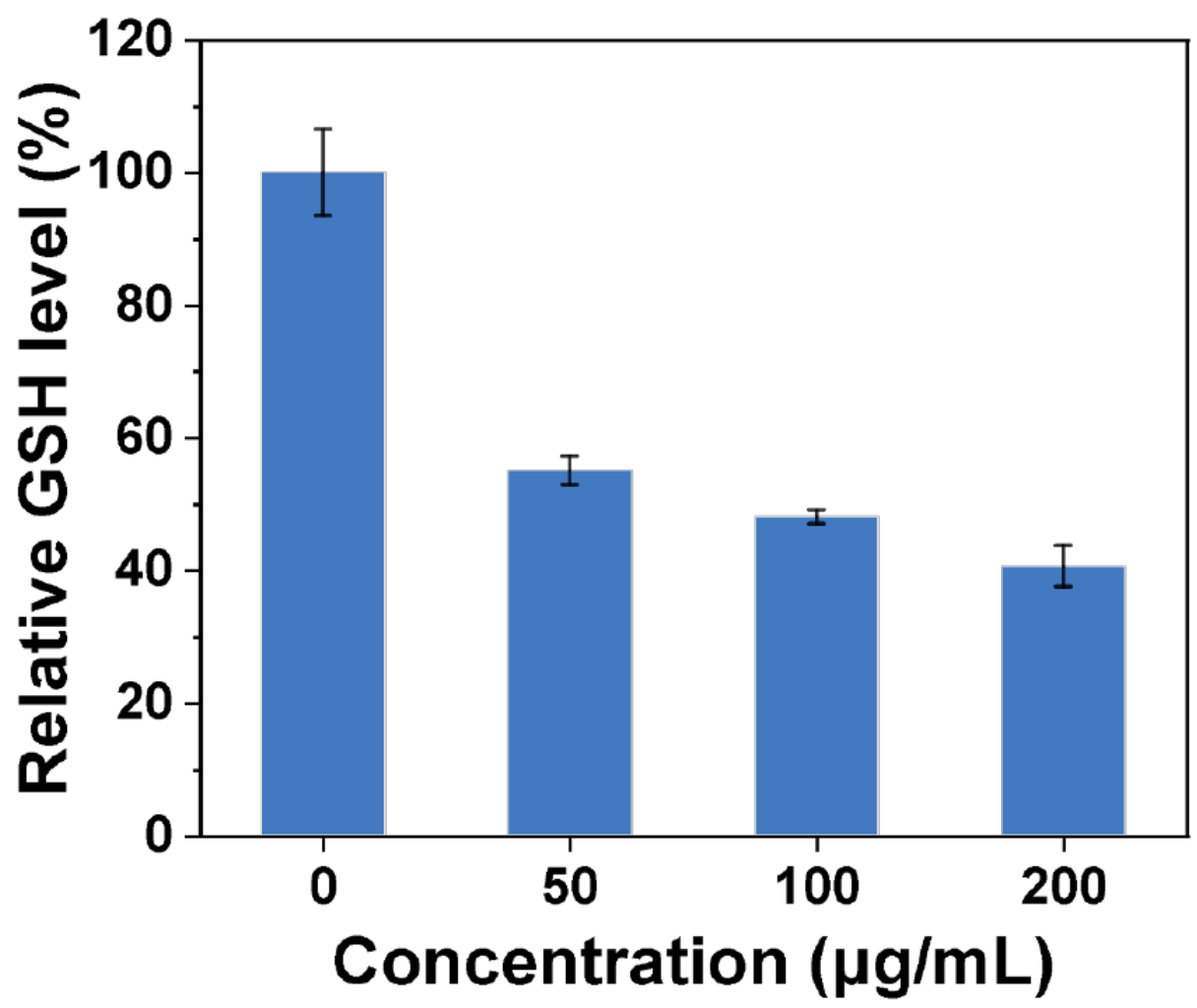

Figure S13. The GSH levels after incubation with FeSS@Au NPs of 0~200 μg/mL by UPLC-MS. The data are presented as mean ± SD (n = 3).

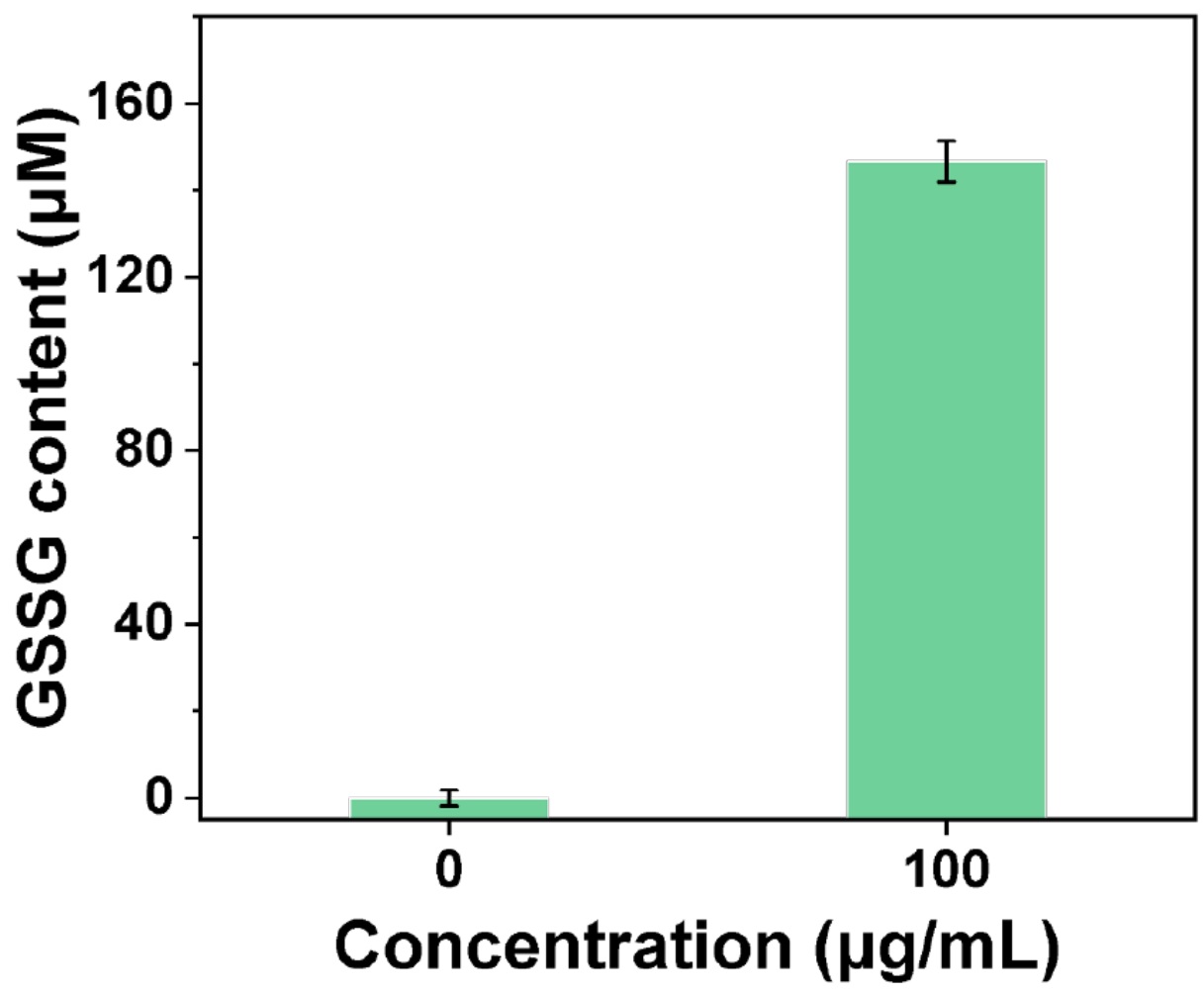

Figure S14. The GSSG contents in GSH solutions after incubation with FeSS@Au NPs of 0 and 100 μg/mL, respectively. The data are presented as mean ± SD (n = 3).

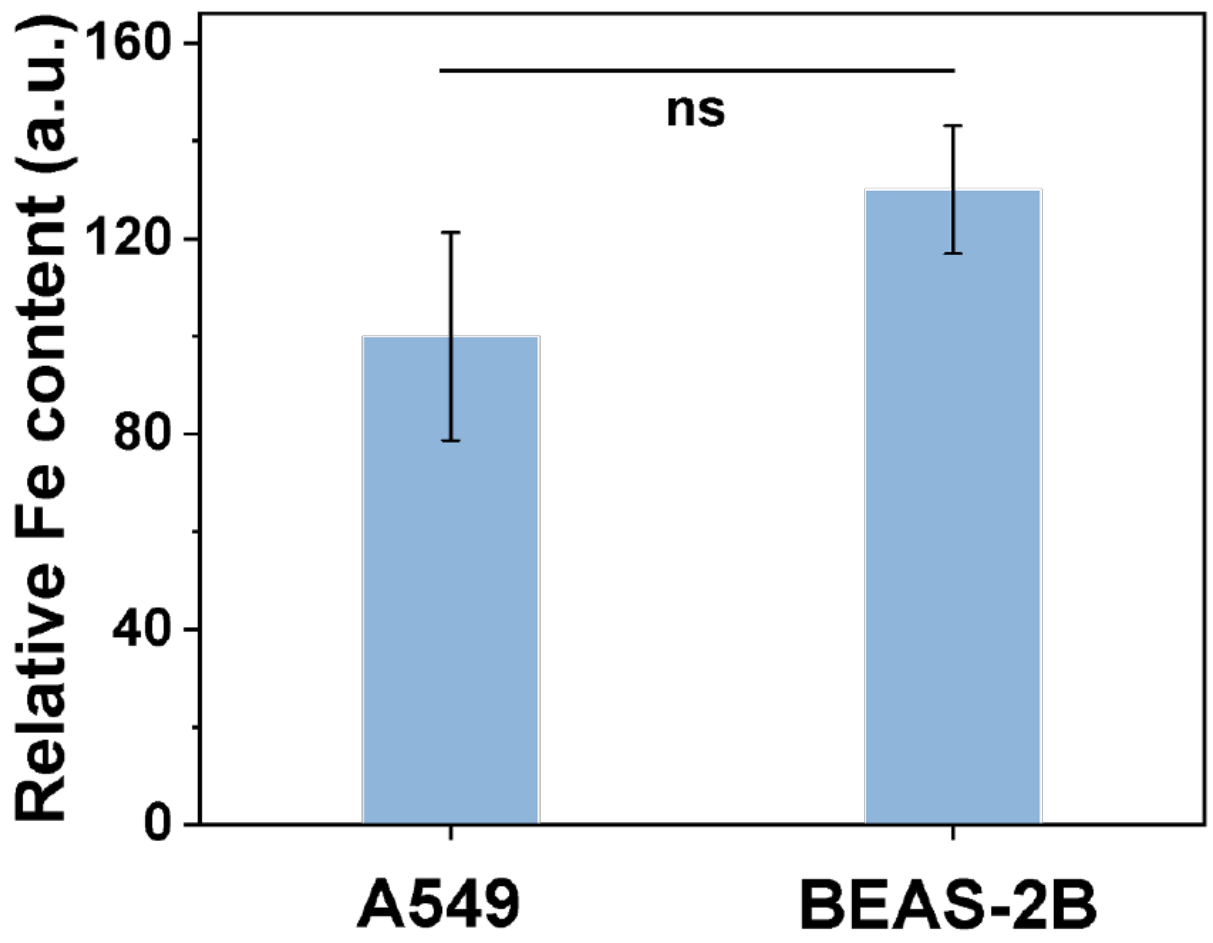

Figure S15. The relative Fe contents of A549 cells and BEAS-2B cells after incubation with FeSS@Au NPs. The data are presented as mean ± SD (n = 3). The data are analyzed by *t*-test.

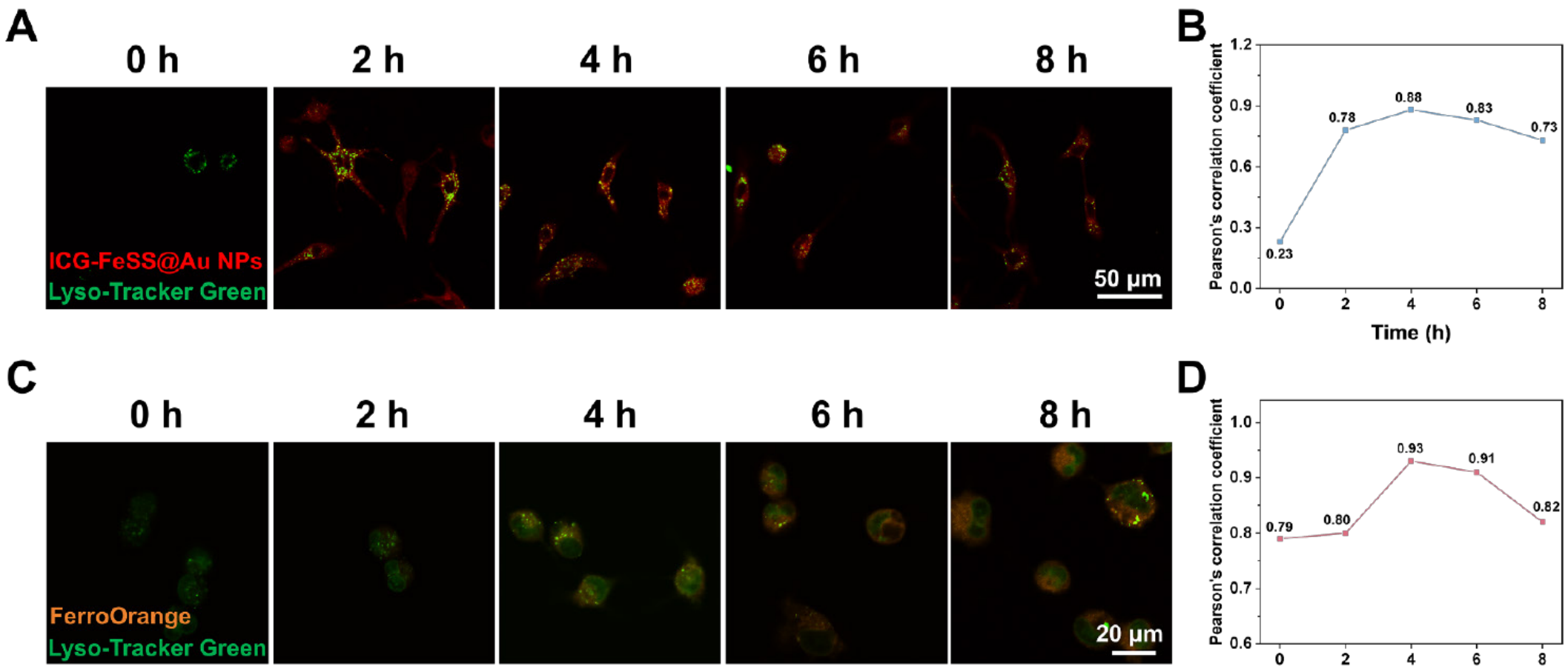

Figure S16. The CLSM images (A) and Pearson's correlation coefficient analysis (B) of A549 cells stained with Lyso-Tracker Green after treatment with ICG-FeSS@Au NPs. The CLSM images (C) and Pearson's correlation coefficient analysis (D) of A549 cells stained with Lyso-Tracker Green and FerroOrange after treatment with FeSS@Au NPs.

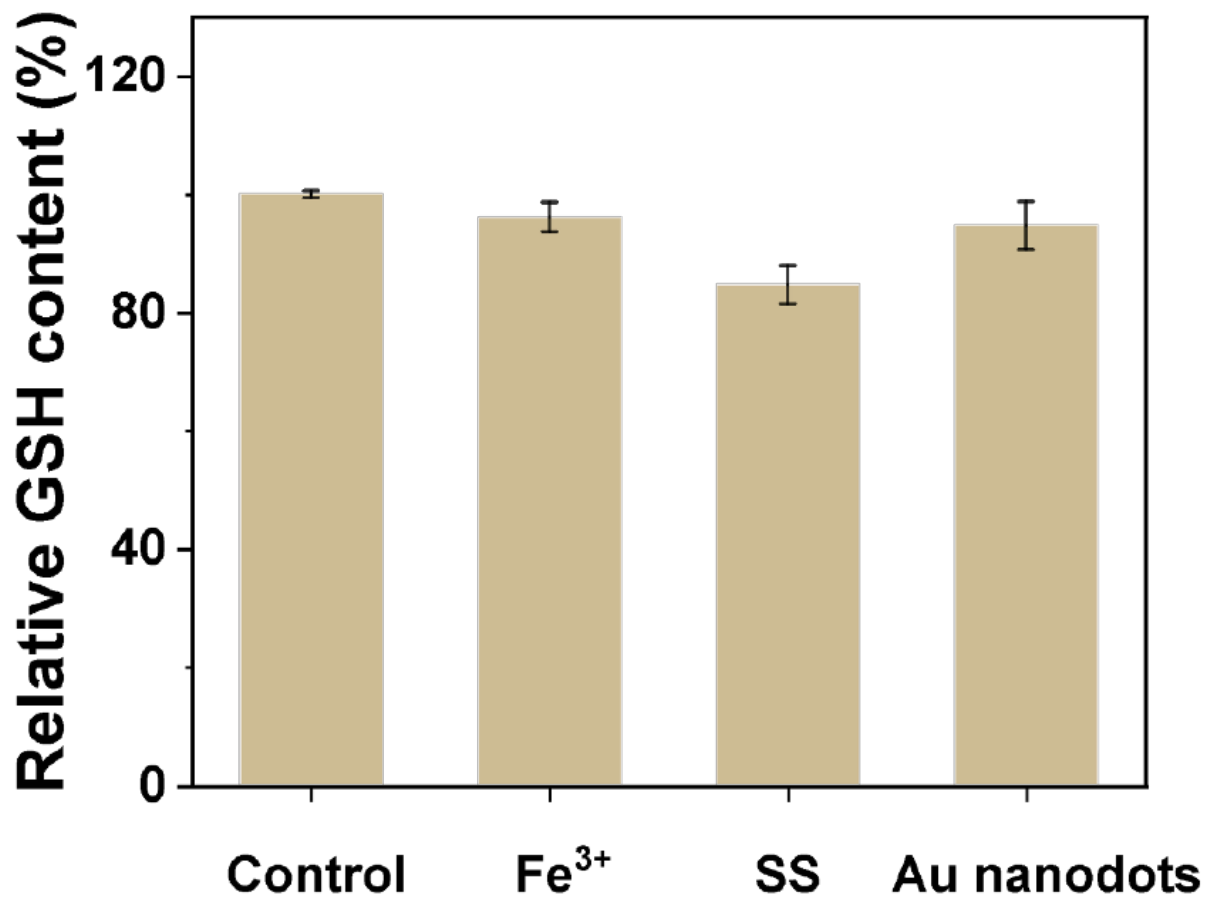

Figure S17. The GSH contents in A549 cells after incubation with Fe^{3+}, SS and Au nanodots. The data are presented as mean ± SD (n = 3).

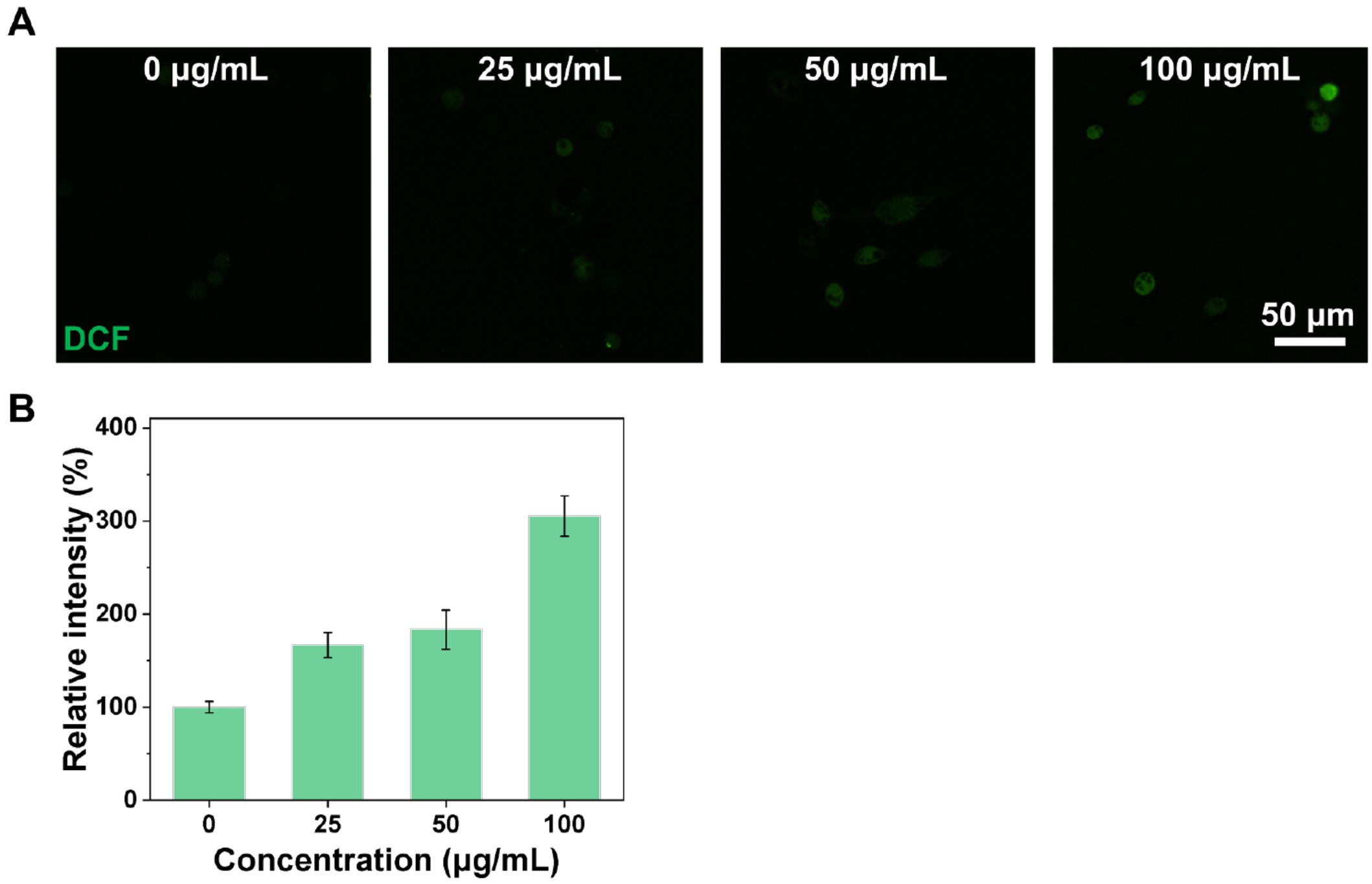

Figure S18. The CLSM images (A) and relative fluorescence intensity (B) of A549 cells stained with DCFH-DA after incubation with FeSS@Au NPs of varied concentrations.

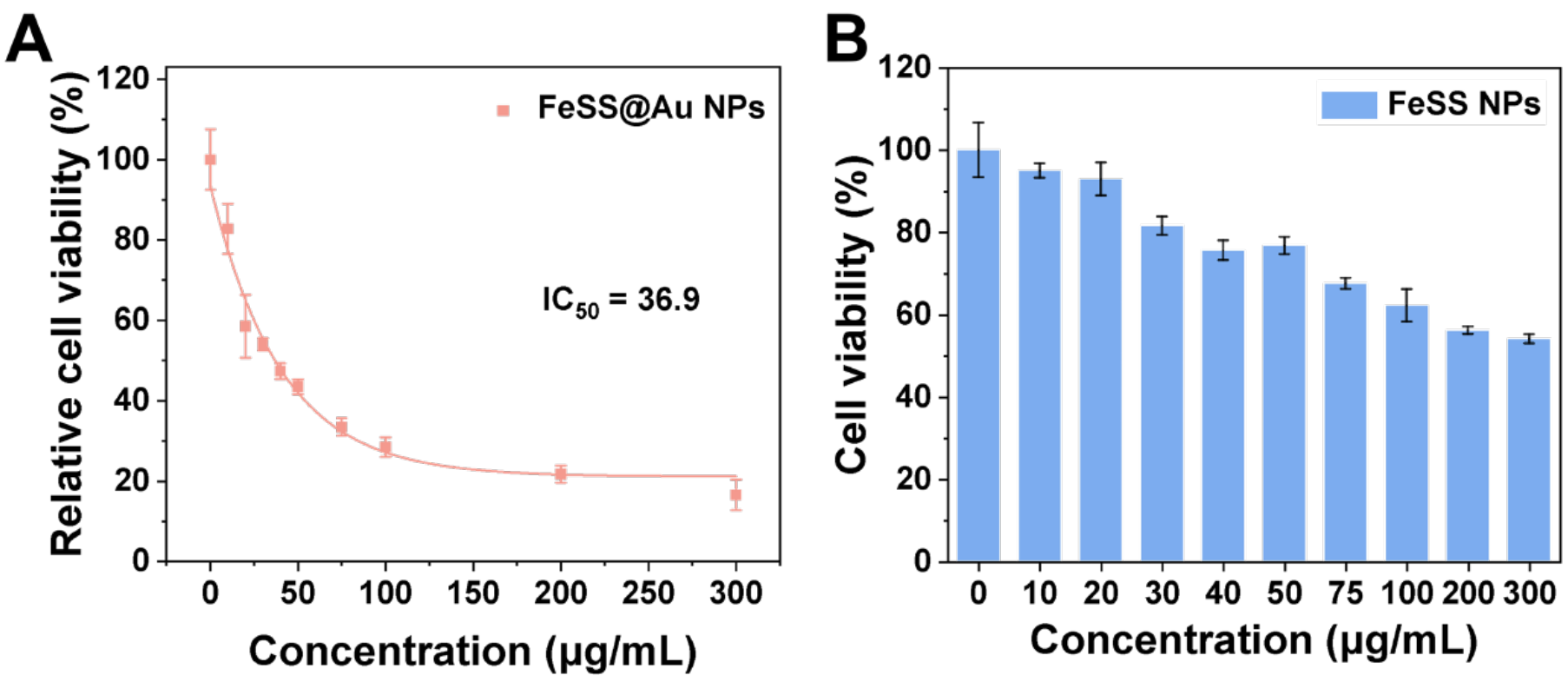

Figure S19. (A) The cell viability and IC50 of A549 cells after incubation with FeSS@Au NPs of varied concentrations. (B) The cell viability of A549 cells after incubation with FeSS NPs of varied concentrations. The data are presented as mean ± SD (n = 3).

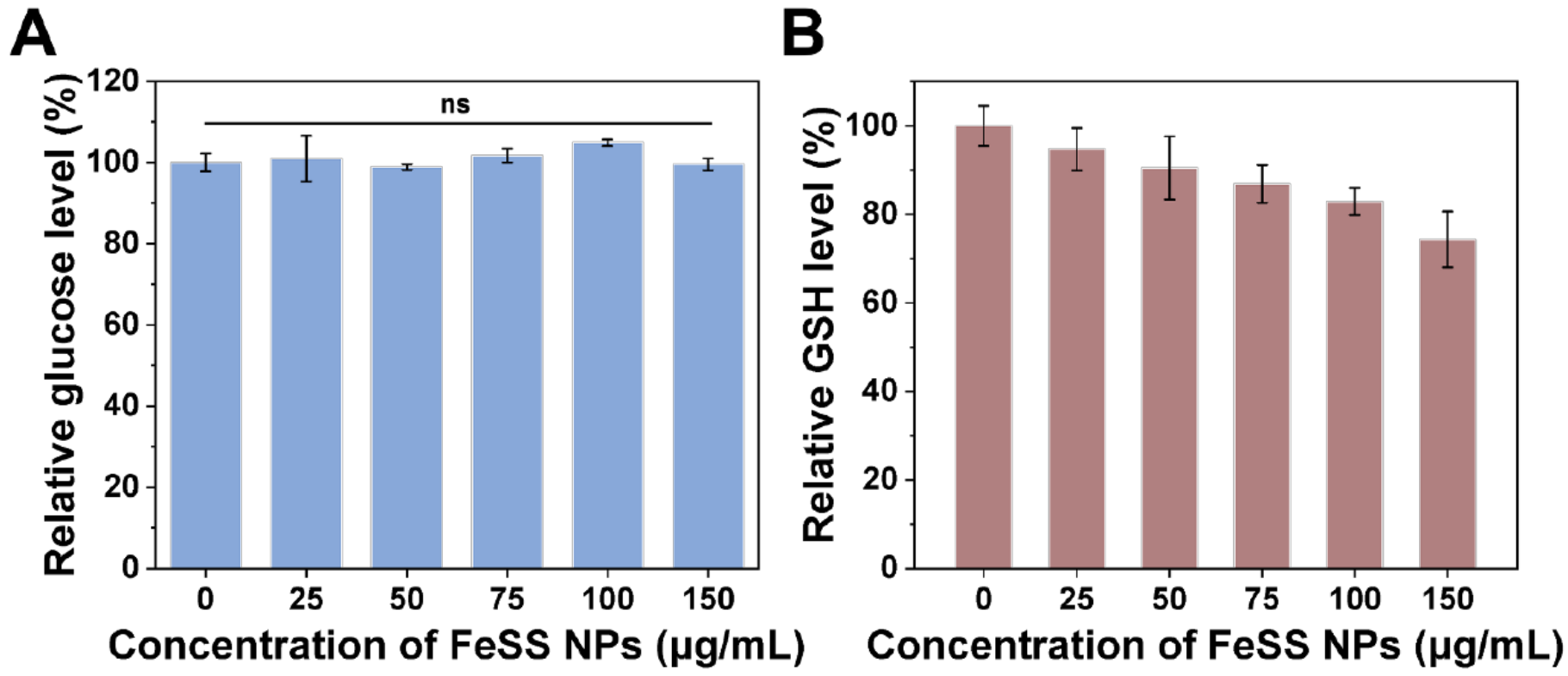

Figure S20. The relative glucose level (A) and GSH level (B) of A549 cells after incubation with FeSS NPs of varied concentrations. The data are presented as mean ± SD (n = 3). The data in Figure S20A are analyzed by one-way ANOVA test.

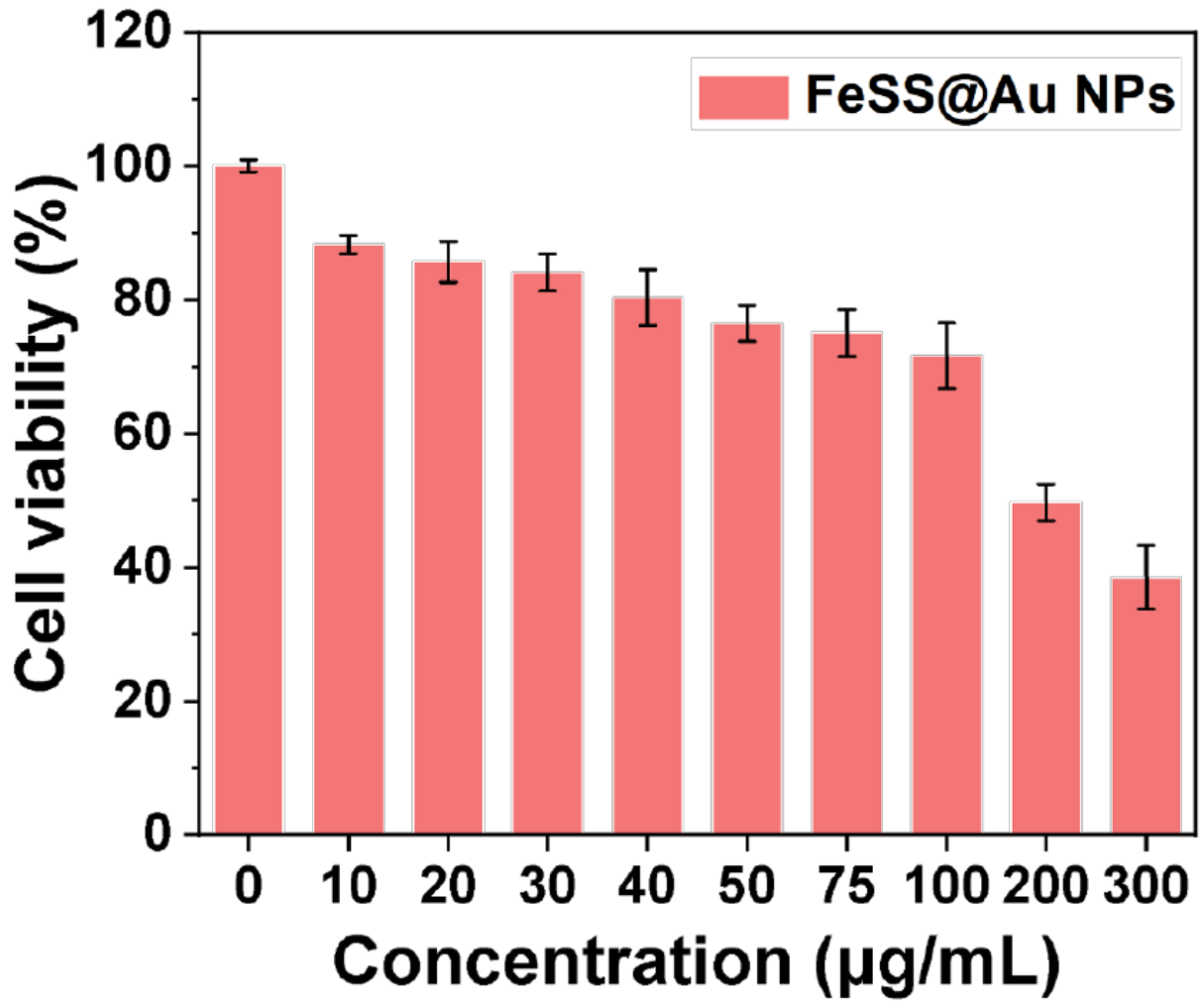

Figure S21. The cell viability of BEAS-2B cells after incubation with FeSS@Au NPs of varied concentrations. The data are presented as mean ± SD (n = 3).

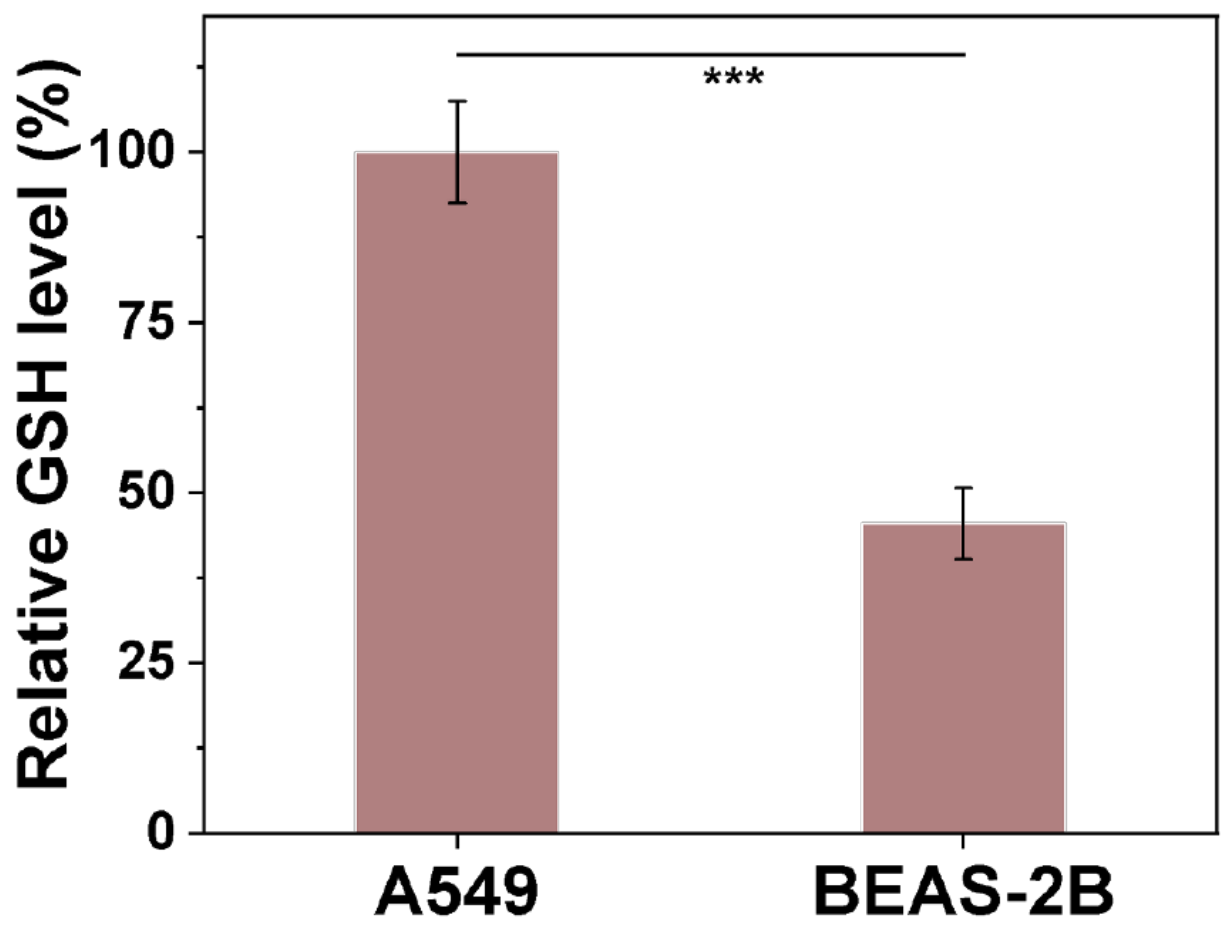

Figure S22. The relative GSH levels of A549 cells and BEAS-2B cells. The data are presented as mean ± SD (n = 3). The data are analyzed by *t*-test. ***$P < 0.001$.

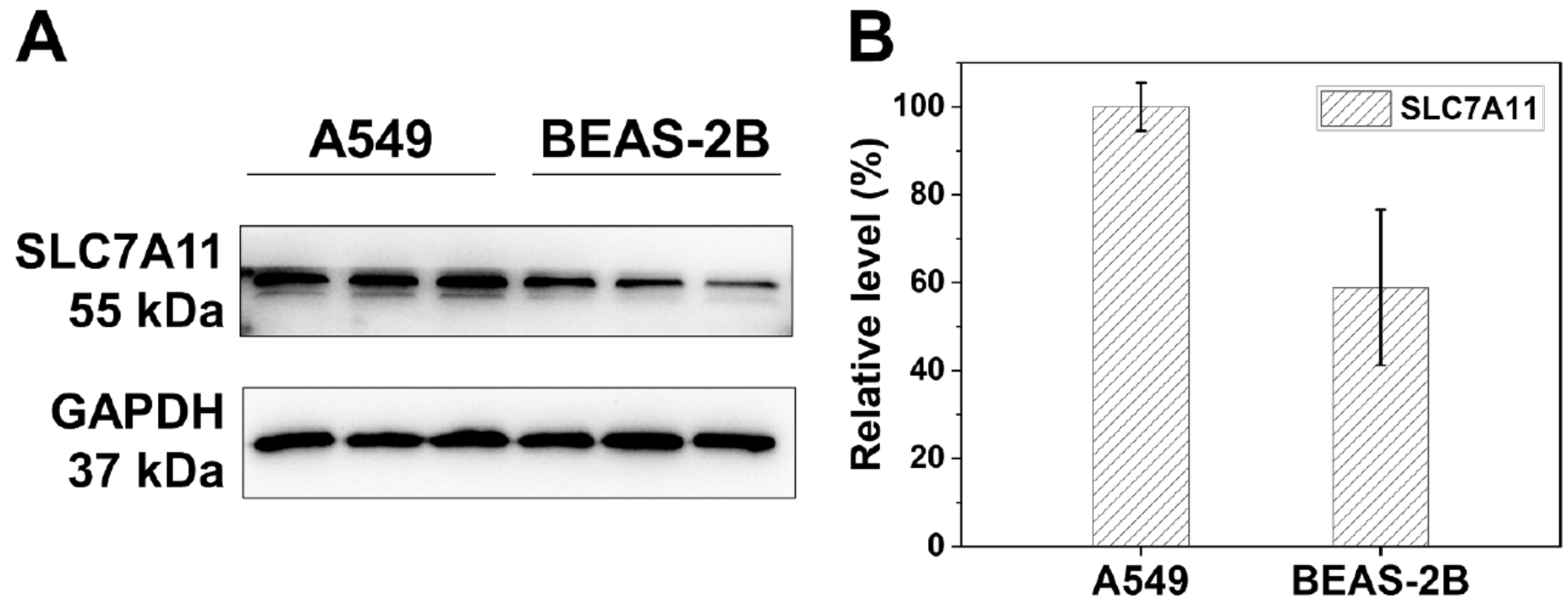

Figure S23. The WB result of SLC7A11 (A) and related quantitative analysis (B) of A549 cells and BEAS-2B cells. The data in Figure S23B are presented as mean ± SD (n = 3).

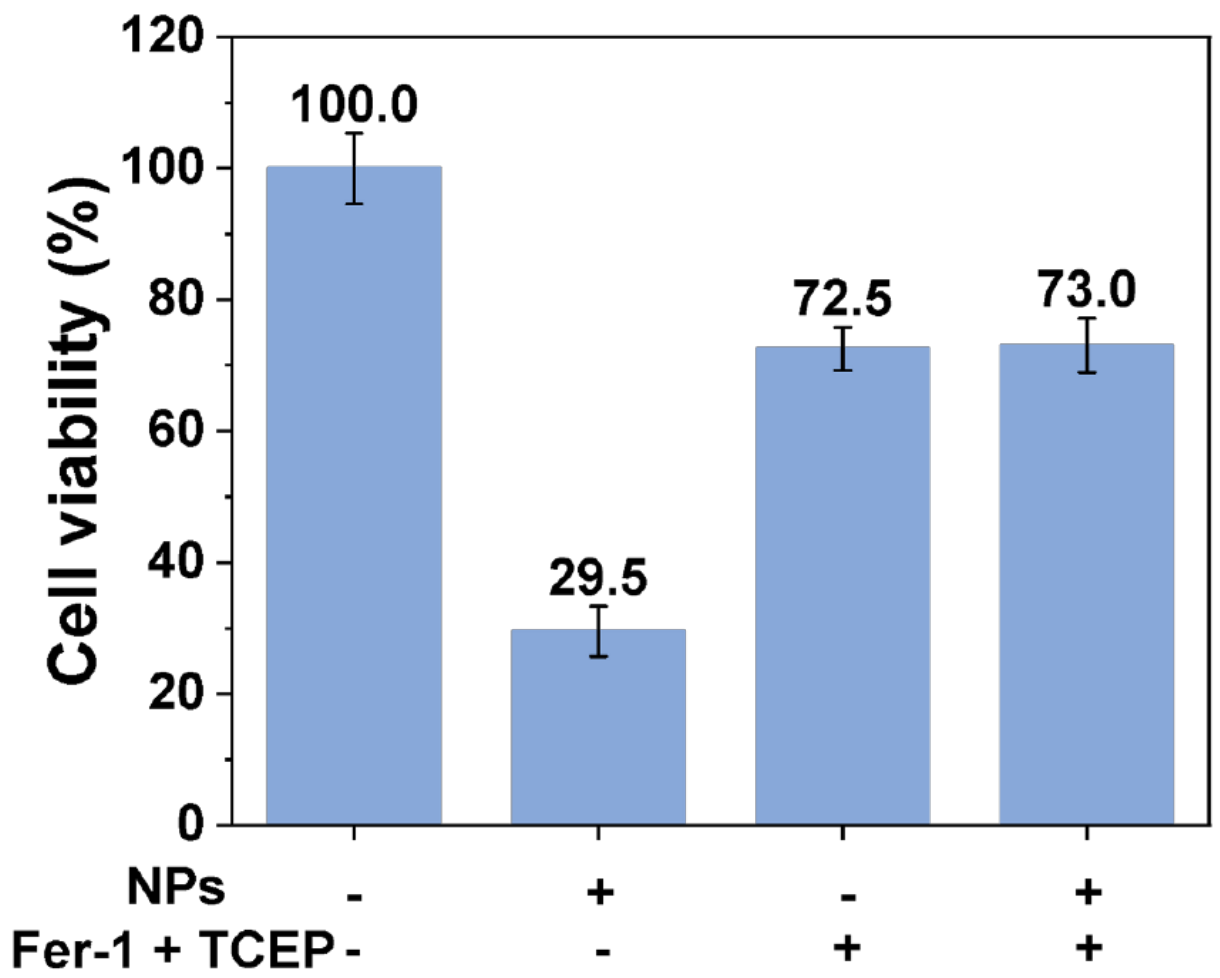

Figure S24. The cell viability of A549 cells after incubation with FeSS@Au NPs and additional inhibitors, including TCEP and Fer-1. The data are presented as mean ± SD (n = 3).

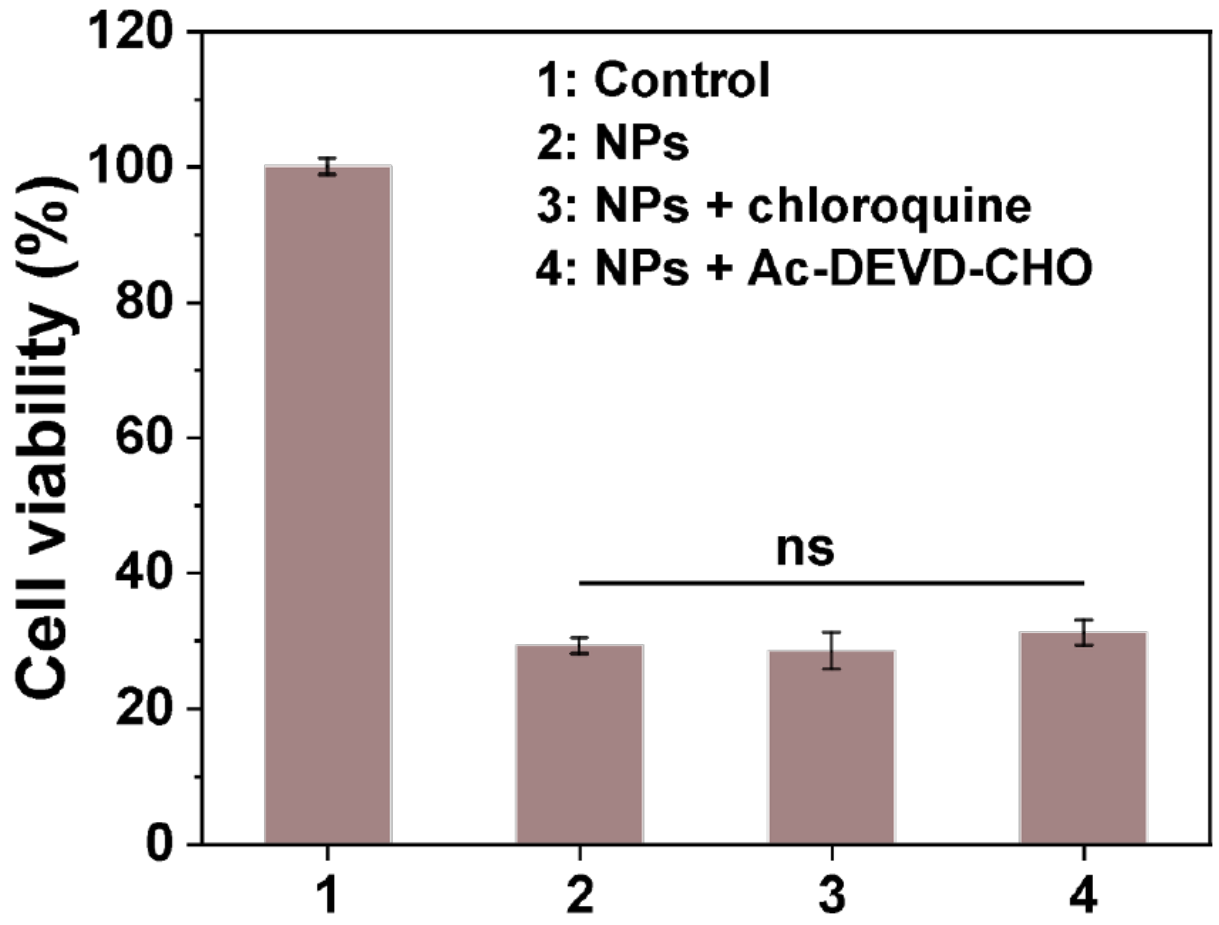

Figure S25. The cell viability of A549 cells after incubation with FeSS@Au NPs and additional inhibitors, including chloroquine and Ac-DEVD-CHO. The data are presented as mean ± SD (n = 3) with one-way ANOVA test.

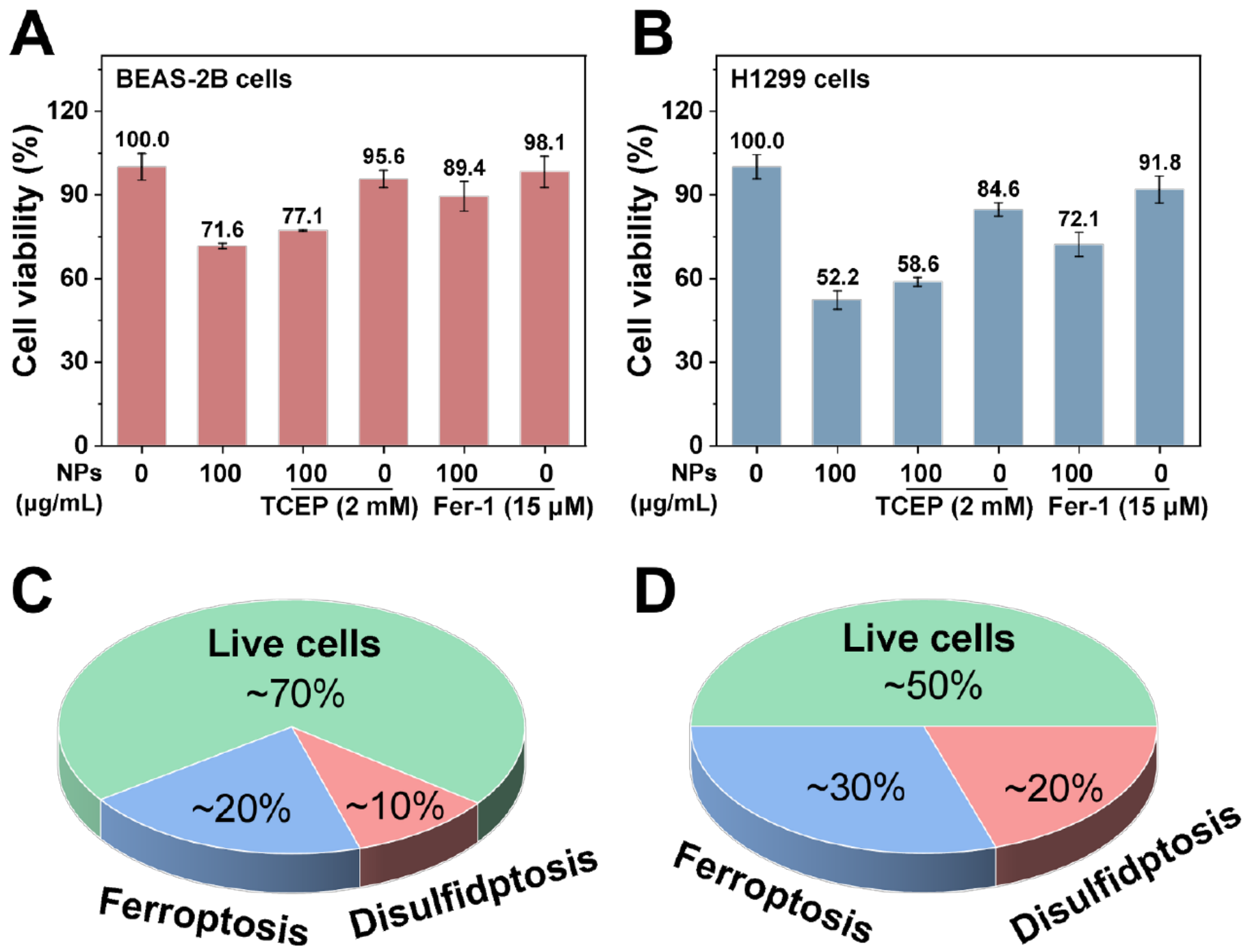

Figure S26. The cell viability of BEAS-2B (A) and H1299 (B) cells after incubation with FeSS@Au NPs and additional TCEP and Fer-1. The calculated ferroptosis and disulfidptosis ratios of BEAS-2B (C) and H1299 (D) cells after FeSS@Au NPs treatment. The data in Figure S26A-B are presented as mean ± SD (n = 3).

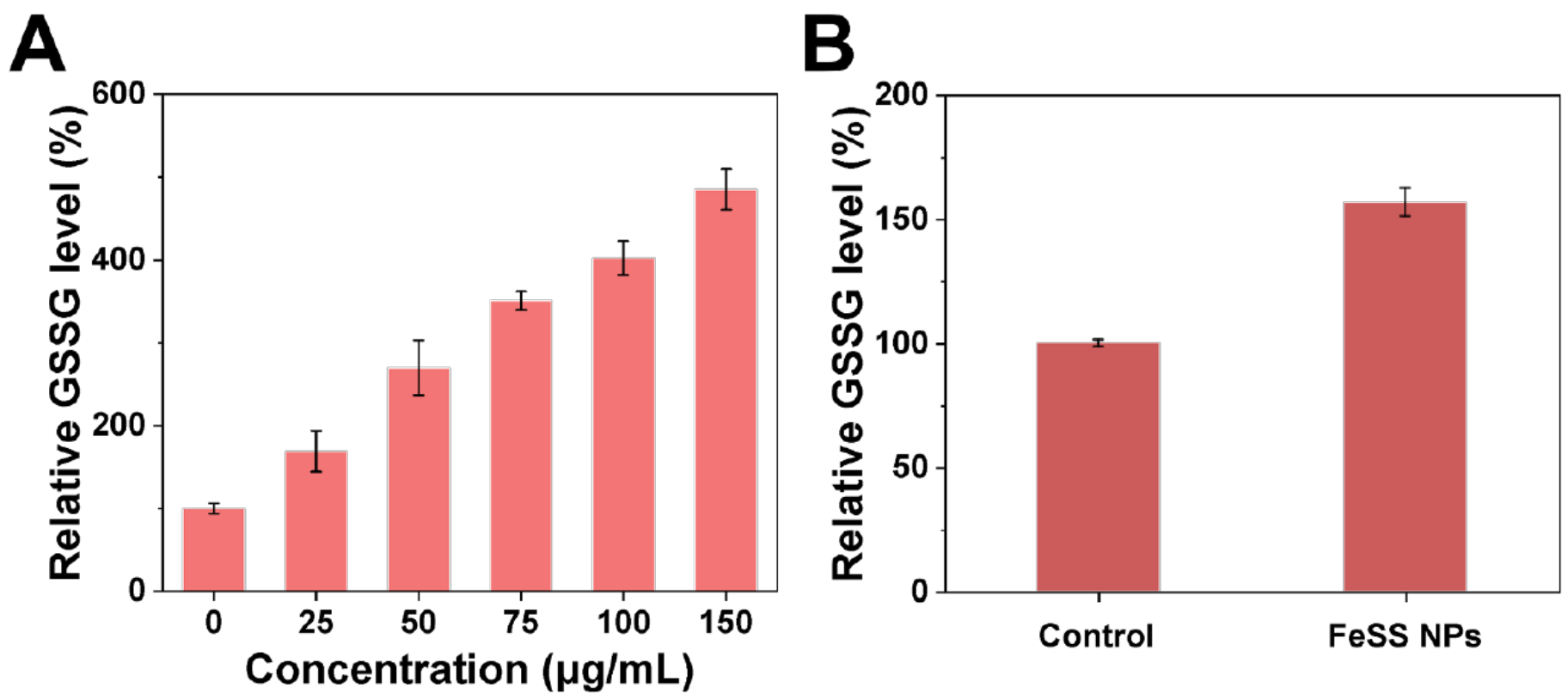

Figure S27. The relative GSSG level of A549 cells after incubation with FeSS@Au NPs of varied concentrations (A) and FeSS NPs of 0 and 100 μg/mL (B). The data are presented as mean ± SD (n = 3).

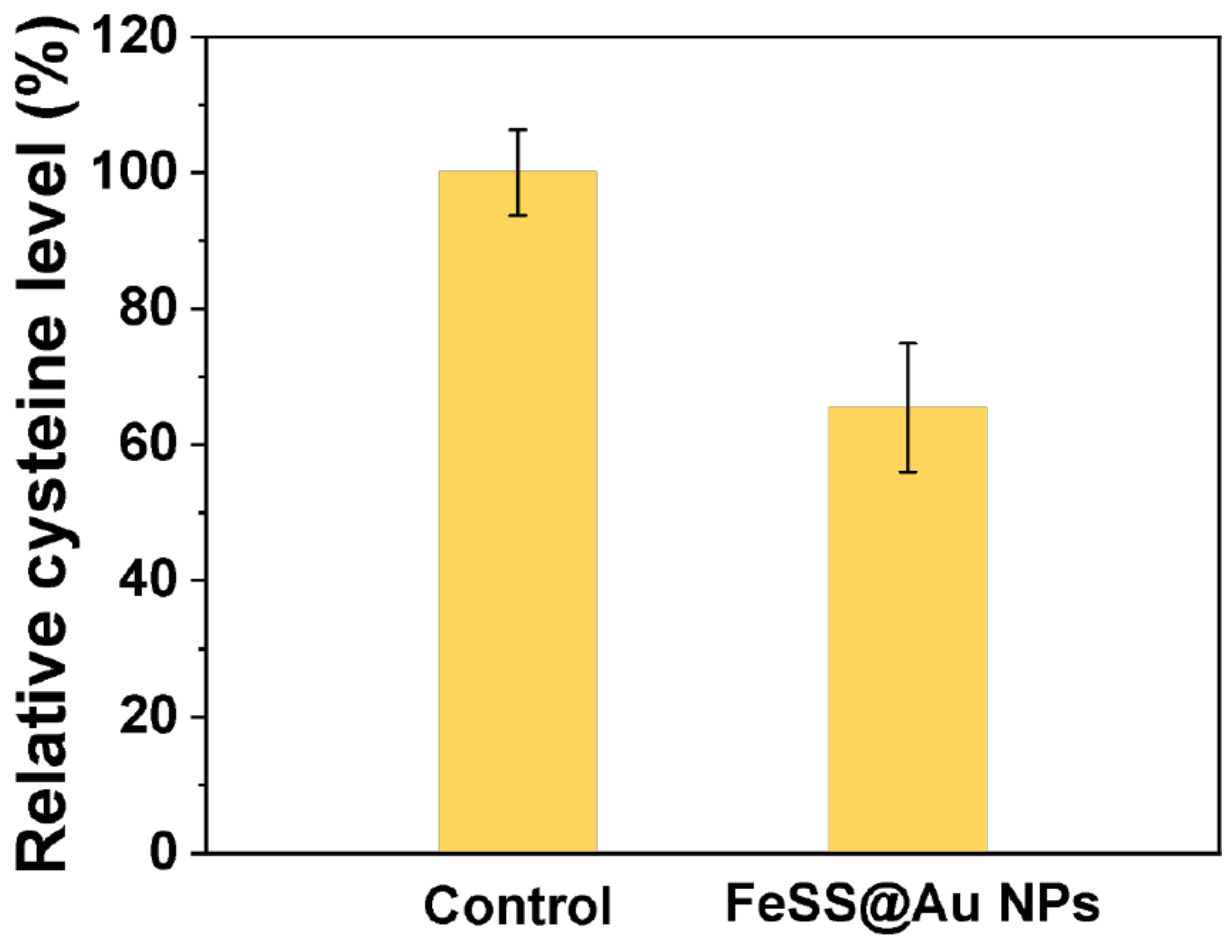

Figure S28. The relative cysteine levels of A549 cells after incubation with FeSS@Au NPs. The data are presented as mean ± SD (n = 3).

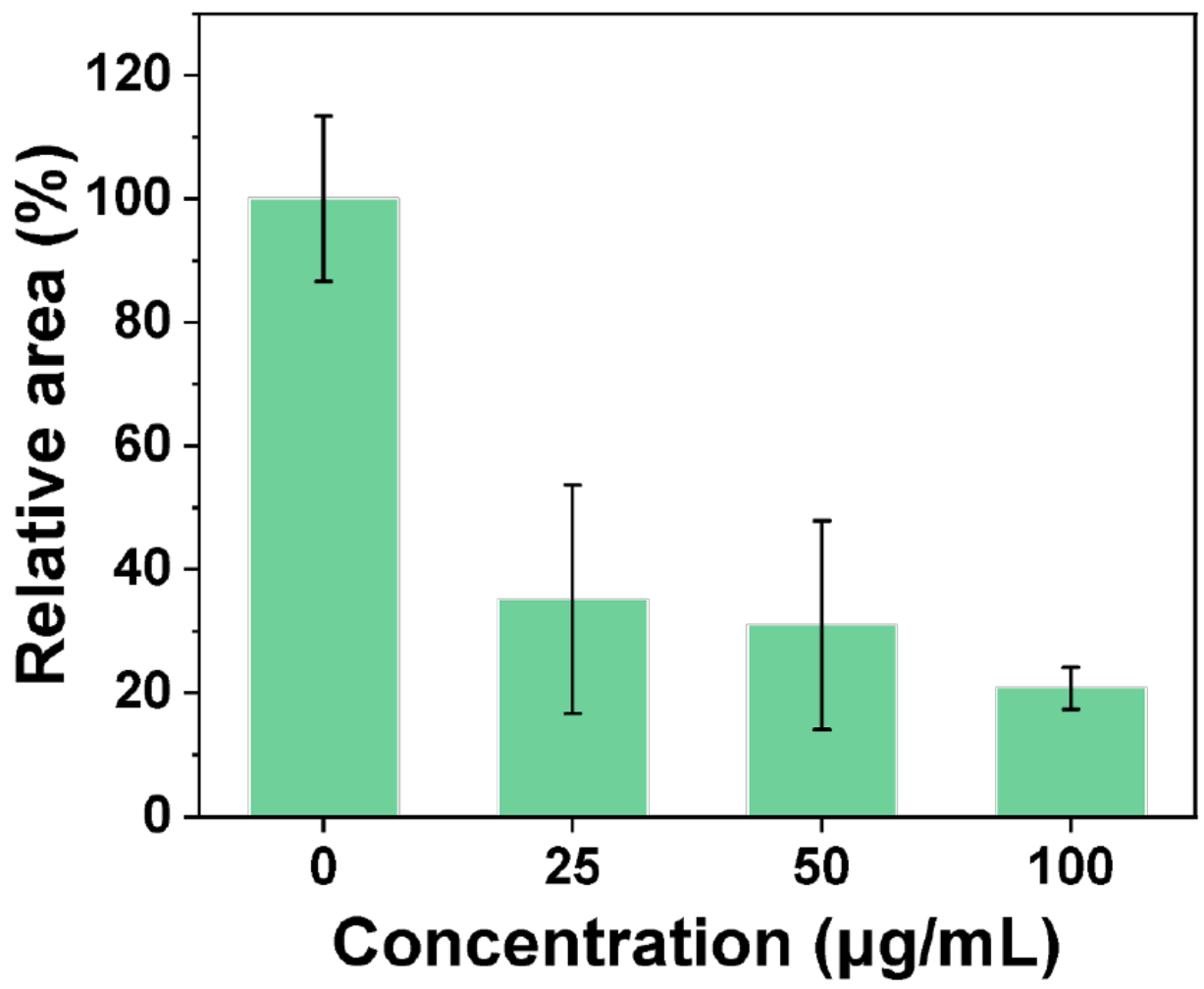

Figure S29. The relative fluorescence area of Actin-Tracker Green in A549 cells in Figure 4G.

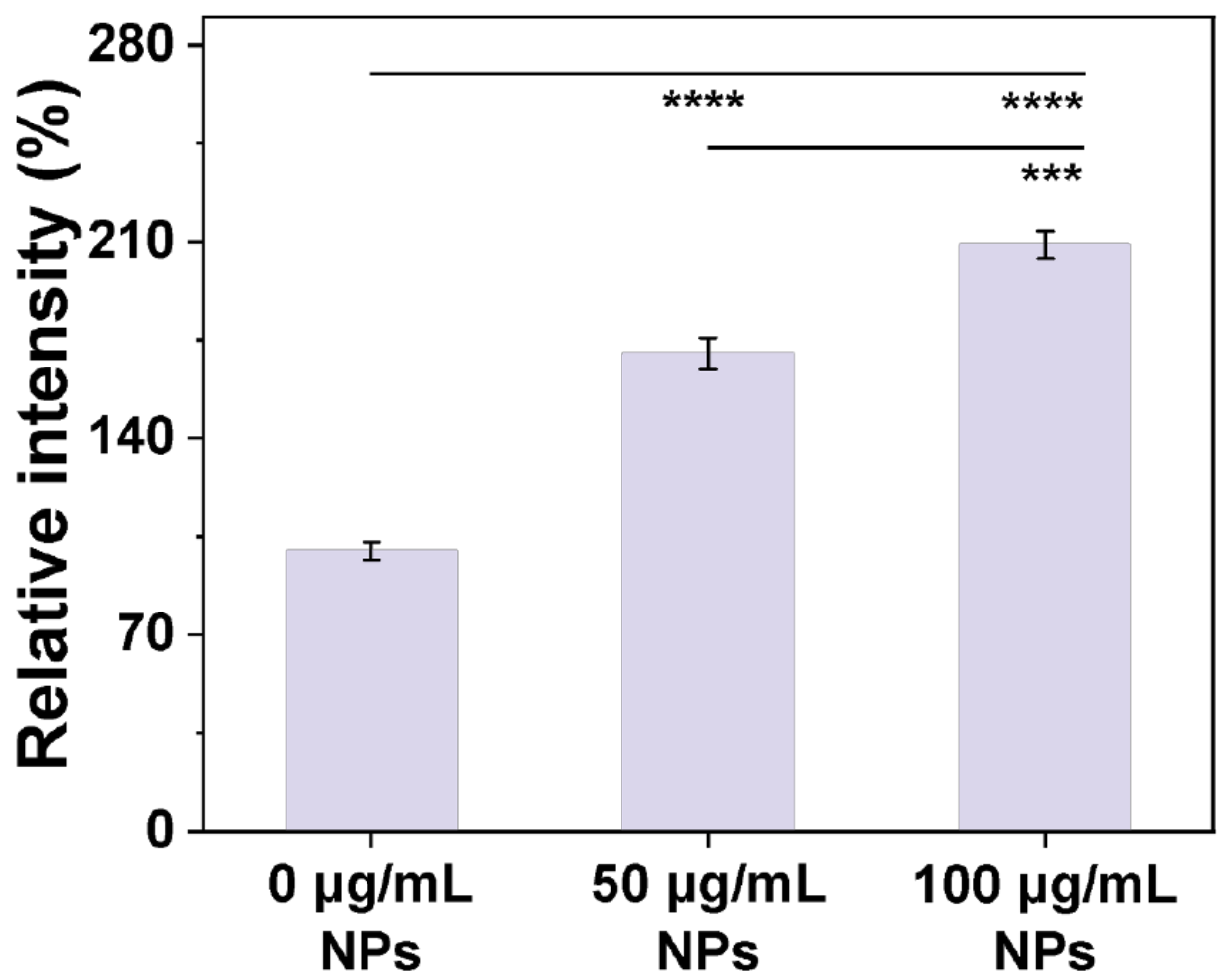

Figure S30. The relative fluorescence intensity of A549 cells stained with DCFH-DA after incubation with FeSS@Au NPs measured by flow cytometry. The data are presented as mean ± SD (n = 3) with one-way ANOVA test. ***$P < 0.001$, and ****$P < 0.0001$.

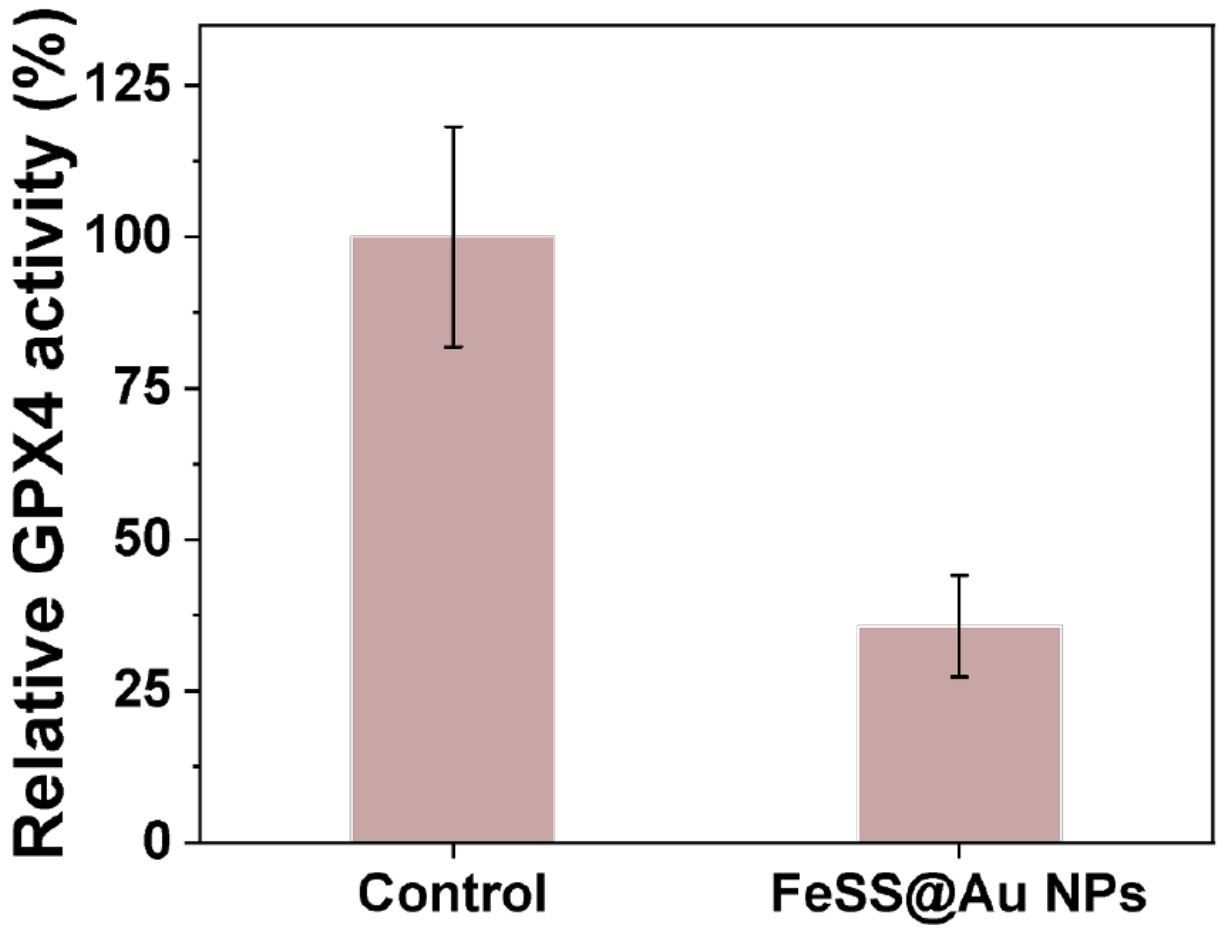

Figure S31. The relative GPX4 activity of A549 cells after incubation with FeSS@Au NPs. The data are presented as mean ± SD (n = 3).

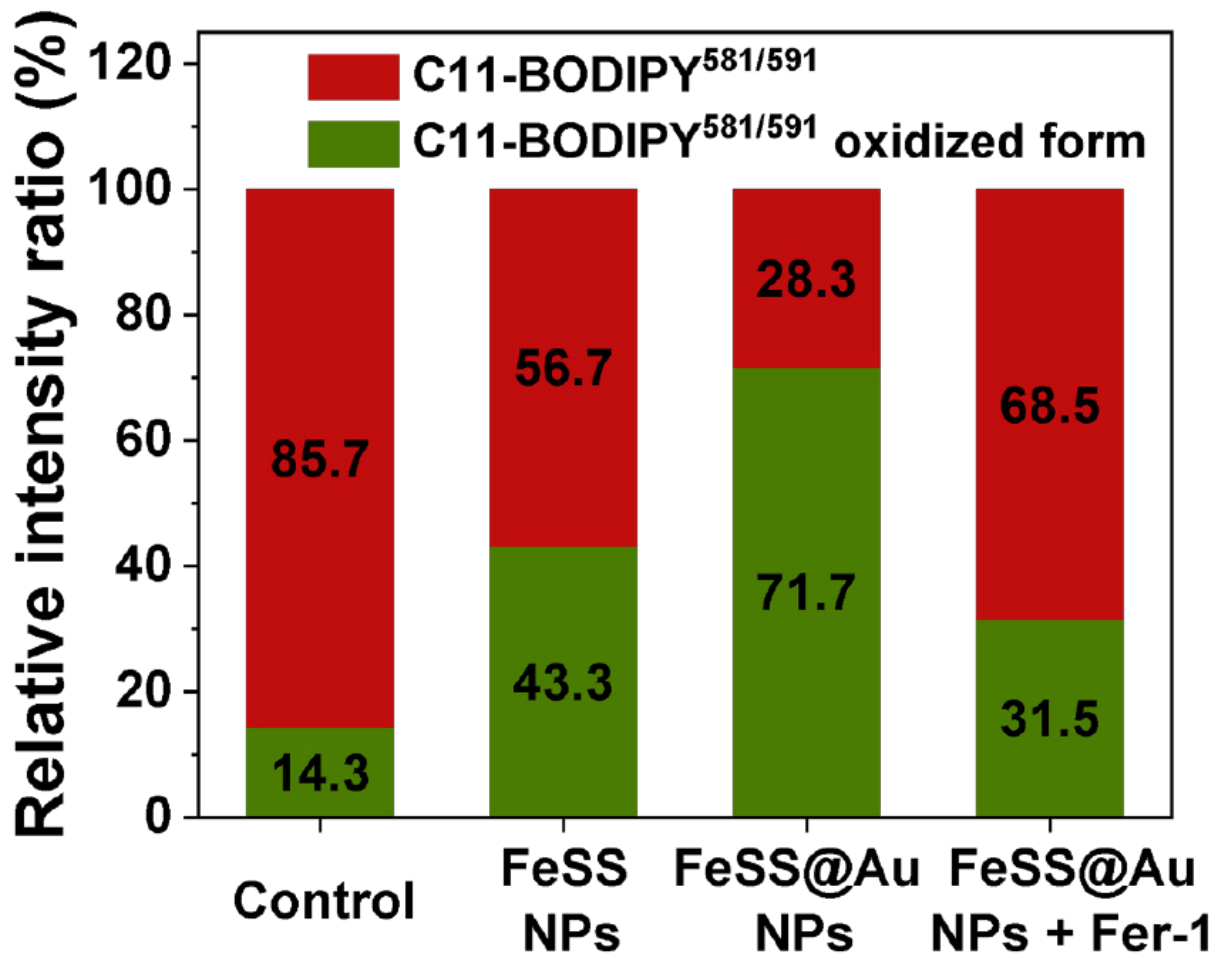

Figure S32. The relative fluorescence intensity of A549 cells in Figure 5E.

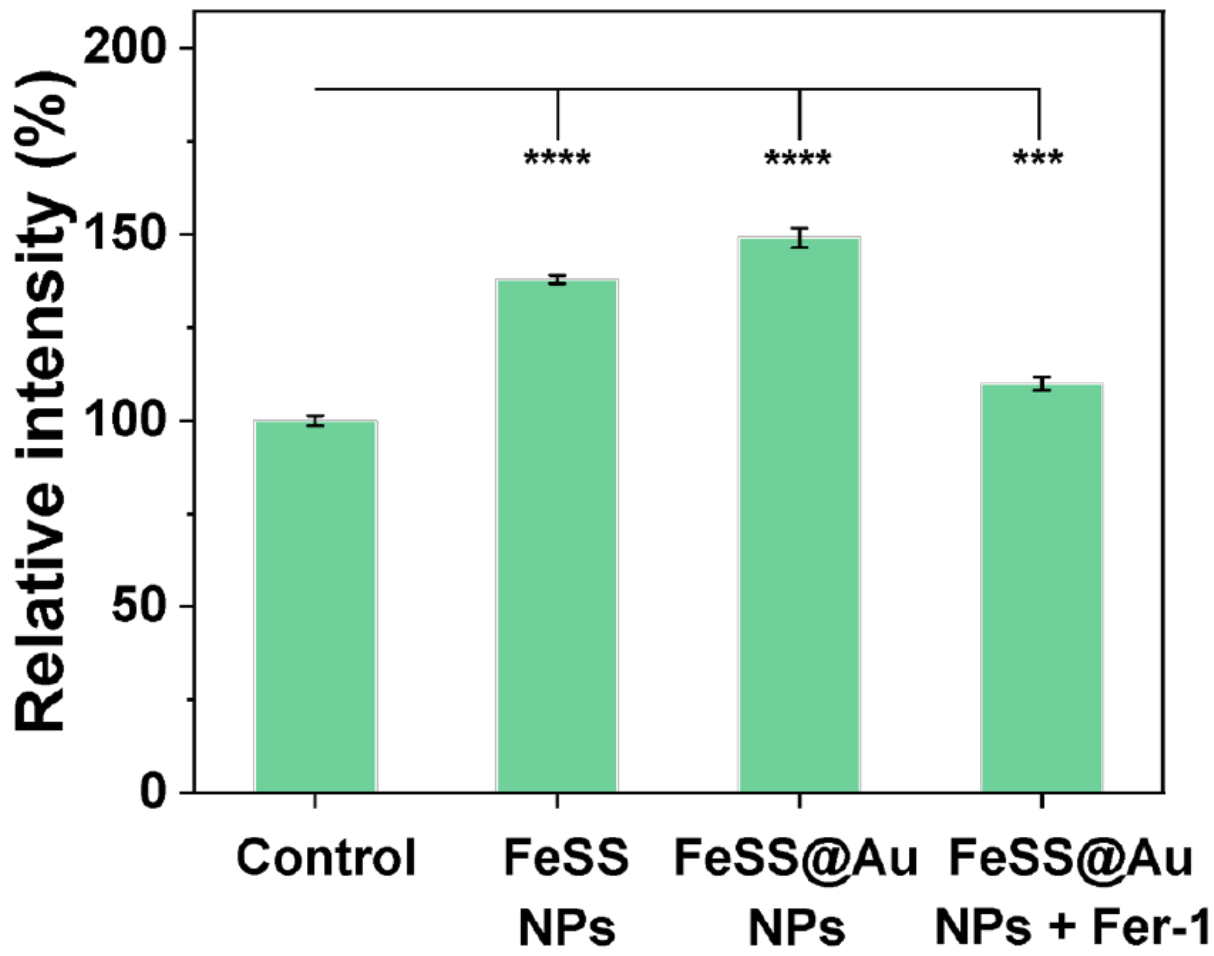

Figure S33. The relative fluorescence intensity of A549 cells stained with C11-BODIPY$^{581/591}$ after incubation with FeSS NPs, FeSS@Au NPs and additional Fer-1 measured by flow cytometry. The data are presented as mean ± SD (n = 3) with one-way ANOVA test. ****P* < 0.001, and *****P* < 0.0001.

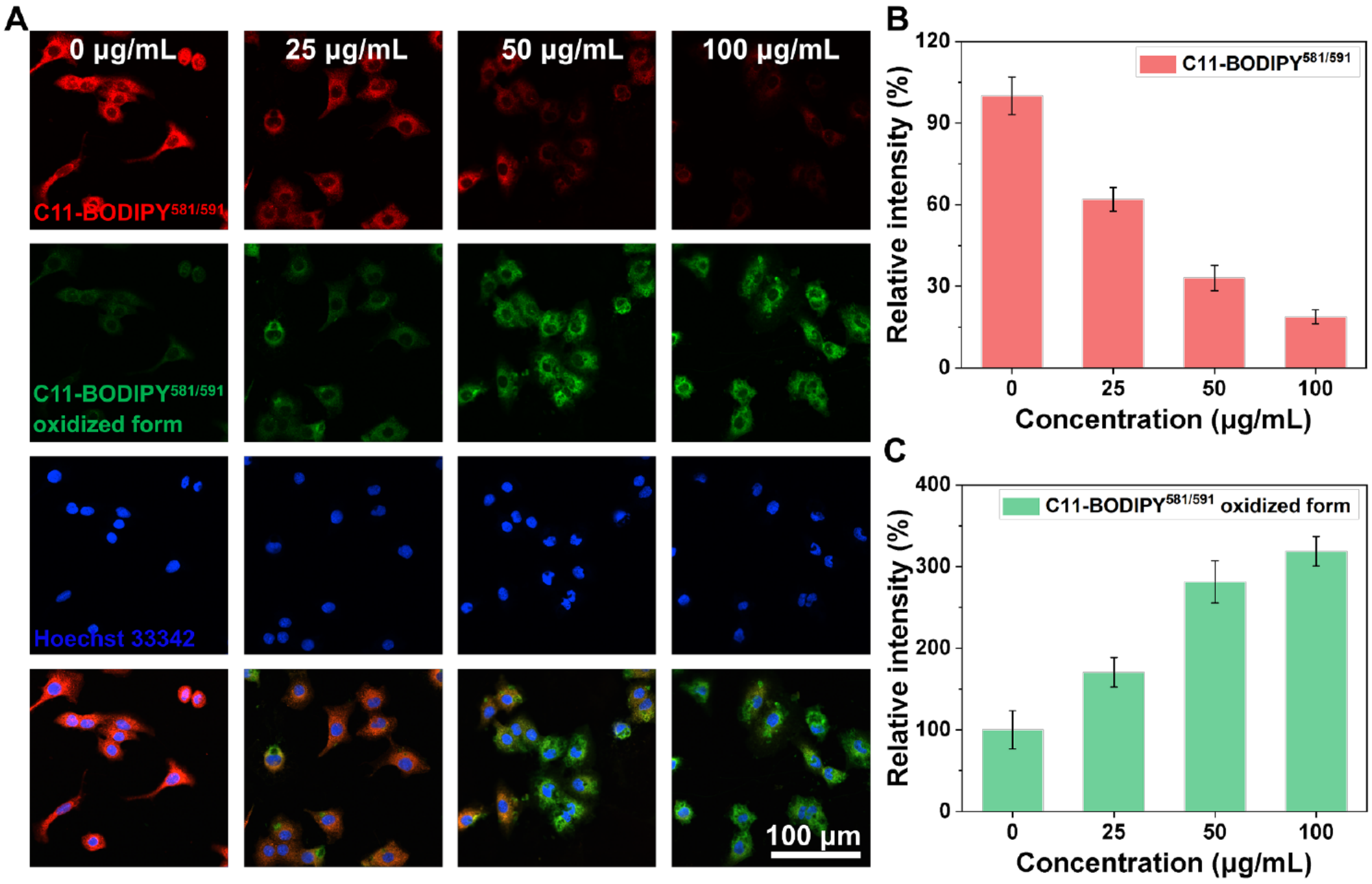

Figure S34. The CLSM images (A) and quantitative analysis of fluorescence intensity (B-C) of A549 cells stained with C11-BODIPY$^{581/591}$ after incubation with FeSS@Au NPs of varied concentrations.

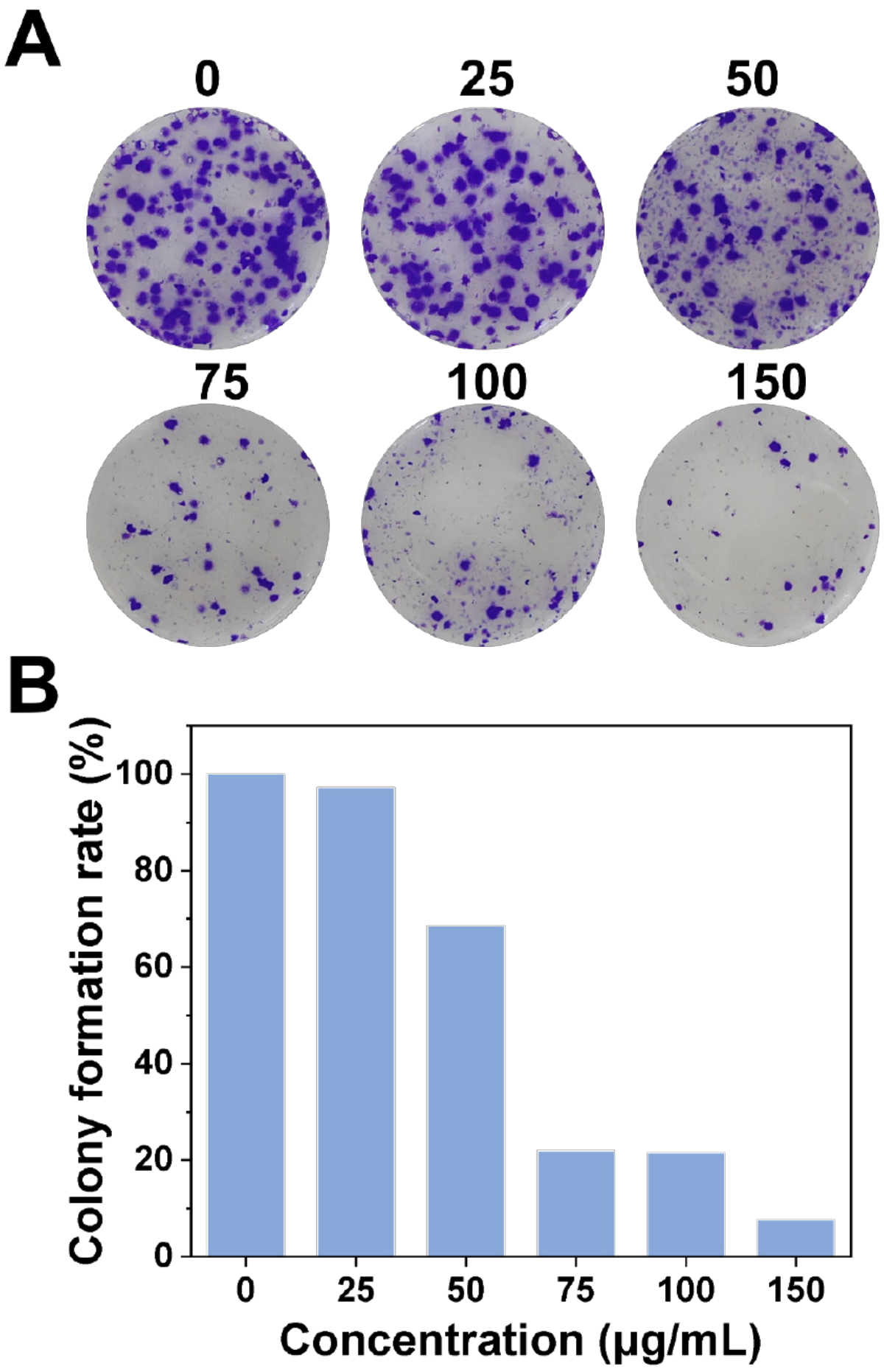

Figure S35. The colony formation pictures (A) and data analysis (B).

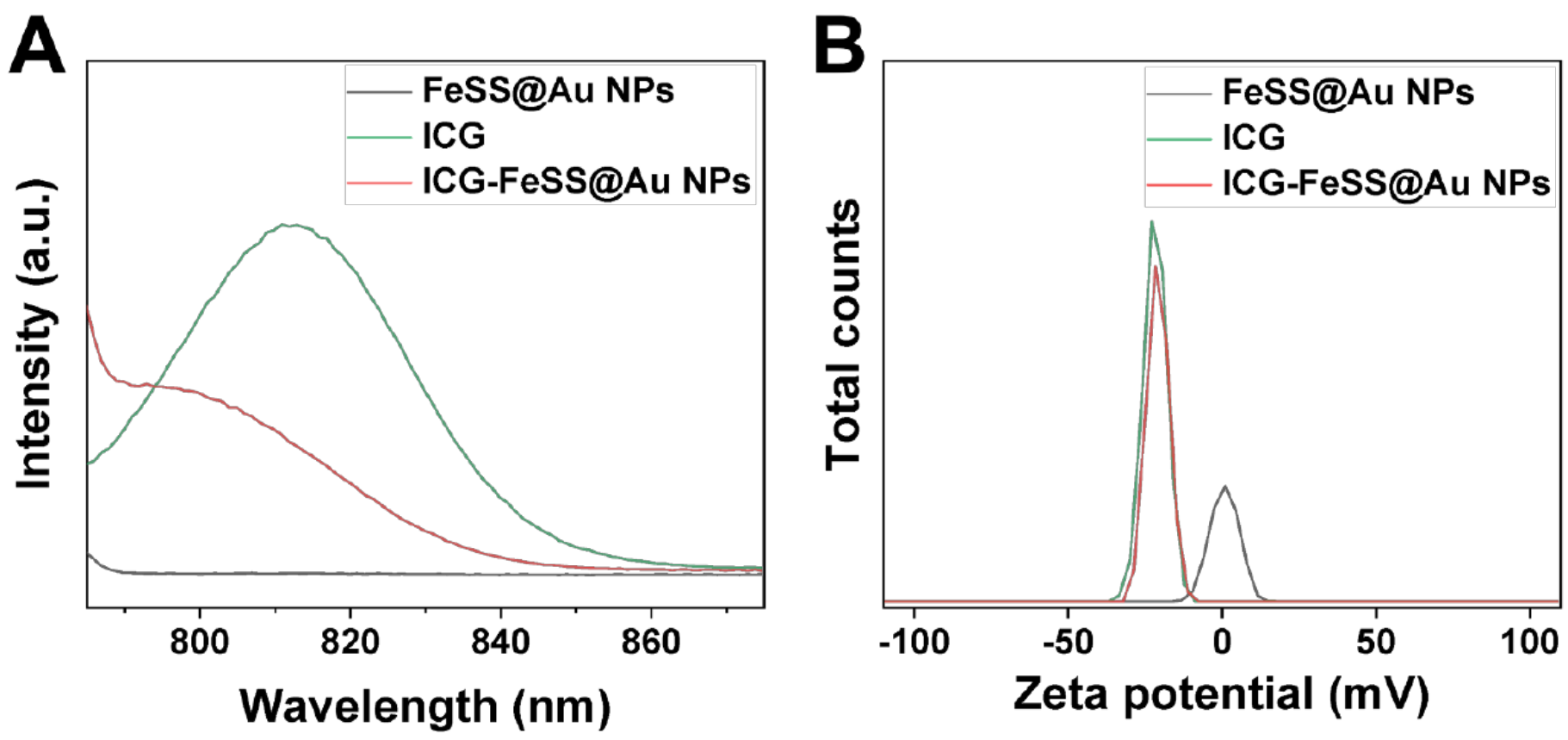

Figure S36. The fluorescence spectra (A) and zeta potentials (B) of FeSS@Au NPs, ICG and ICG-FeSS@Au NPs.

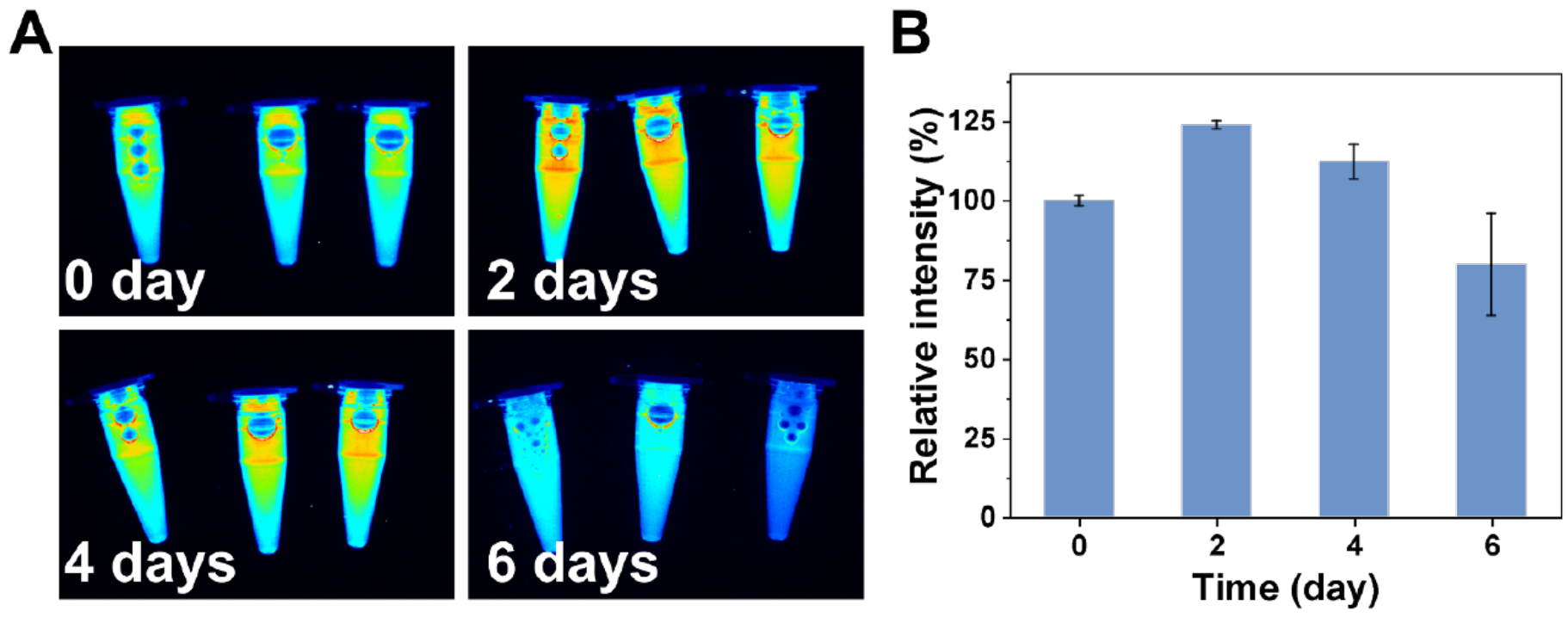

Figure S37. The fluorescence images (A) and relative fluorescence intensity (B) of ICG-FeSS@Au NPs after incubation with culture medium for 0, 2, 4 and 6 days. The data in Figure S37B are presented as mean ± SD (n = 3).

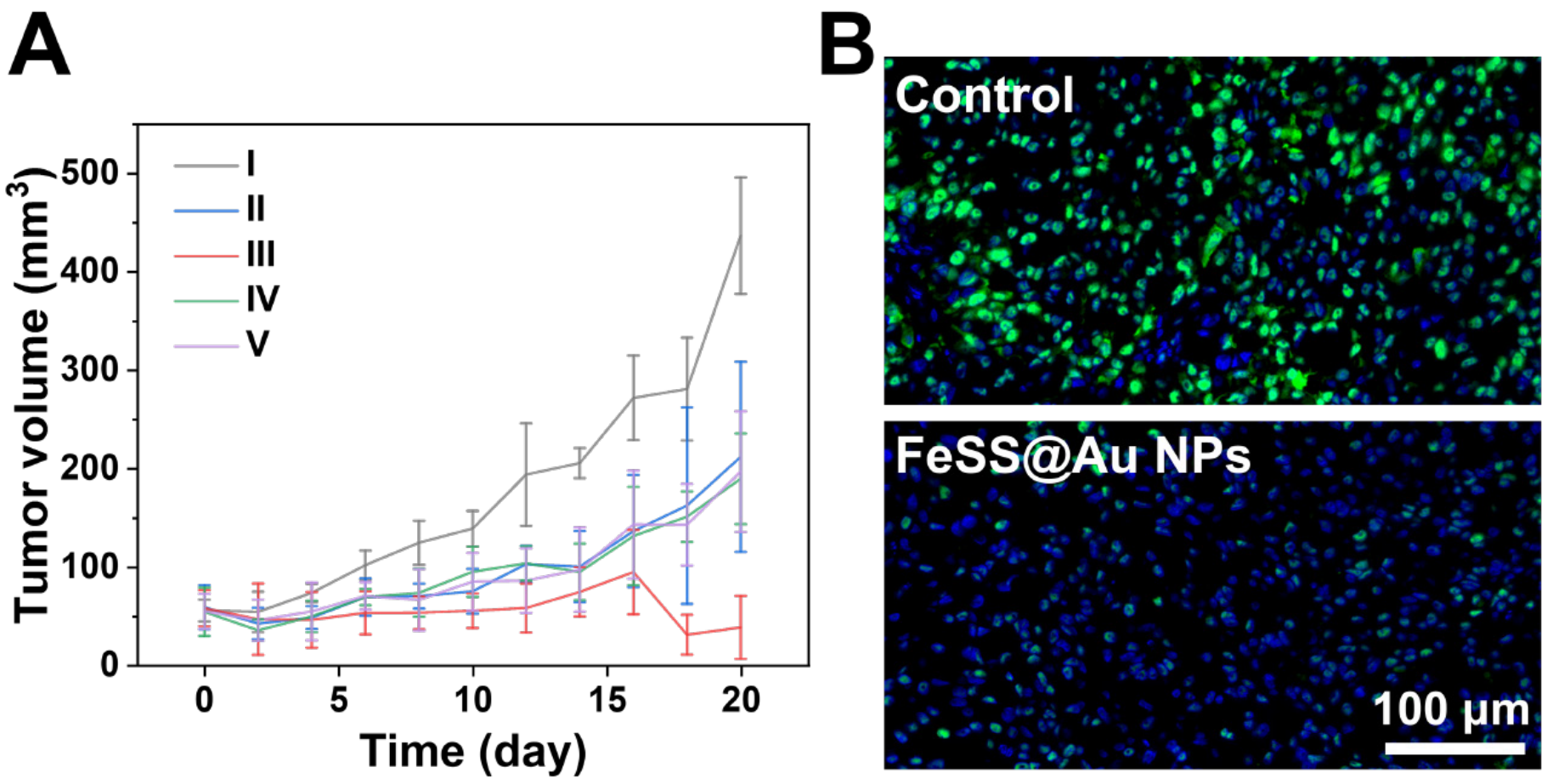

Figure S38. (A) The average tumor volume of group I-V in Figure 7C. (B) The Ki67 stained tumor slices of group I and III. The data in Figure S38A are presented as mean ± SD (n = 5).

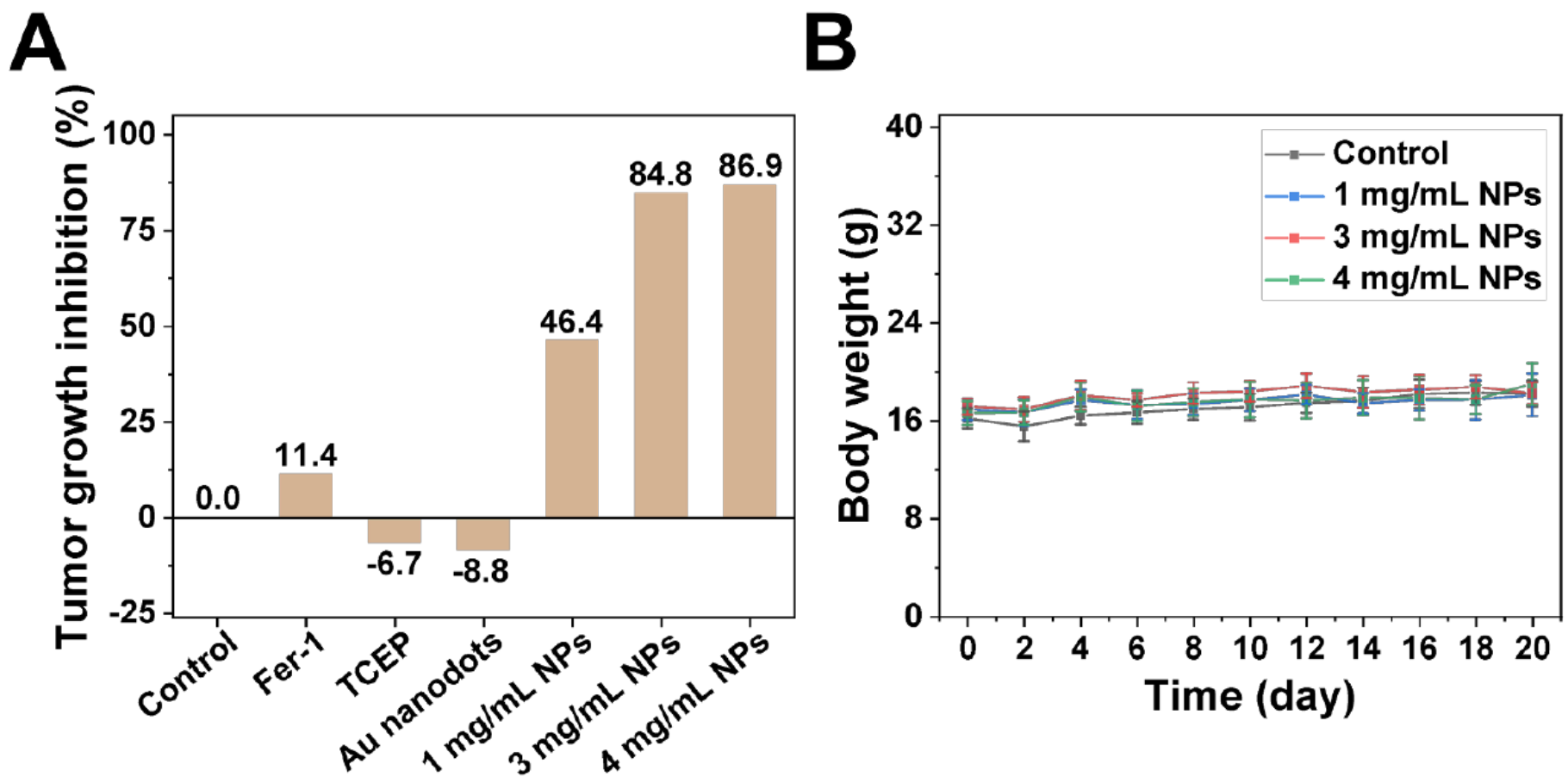

Figure S39. (A) Tumor growth inhibition rates of mice in different treatment groups. (B) The body weight of mice in groups treated with FeSS@Au NPs of 0~4 mg/mL. The data in Figure S39B are presented as mean ± SD (n = 5).

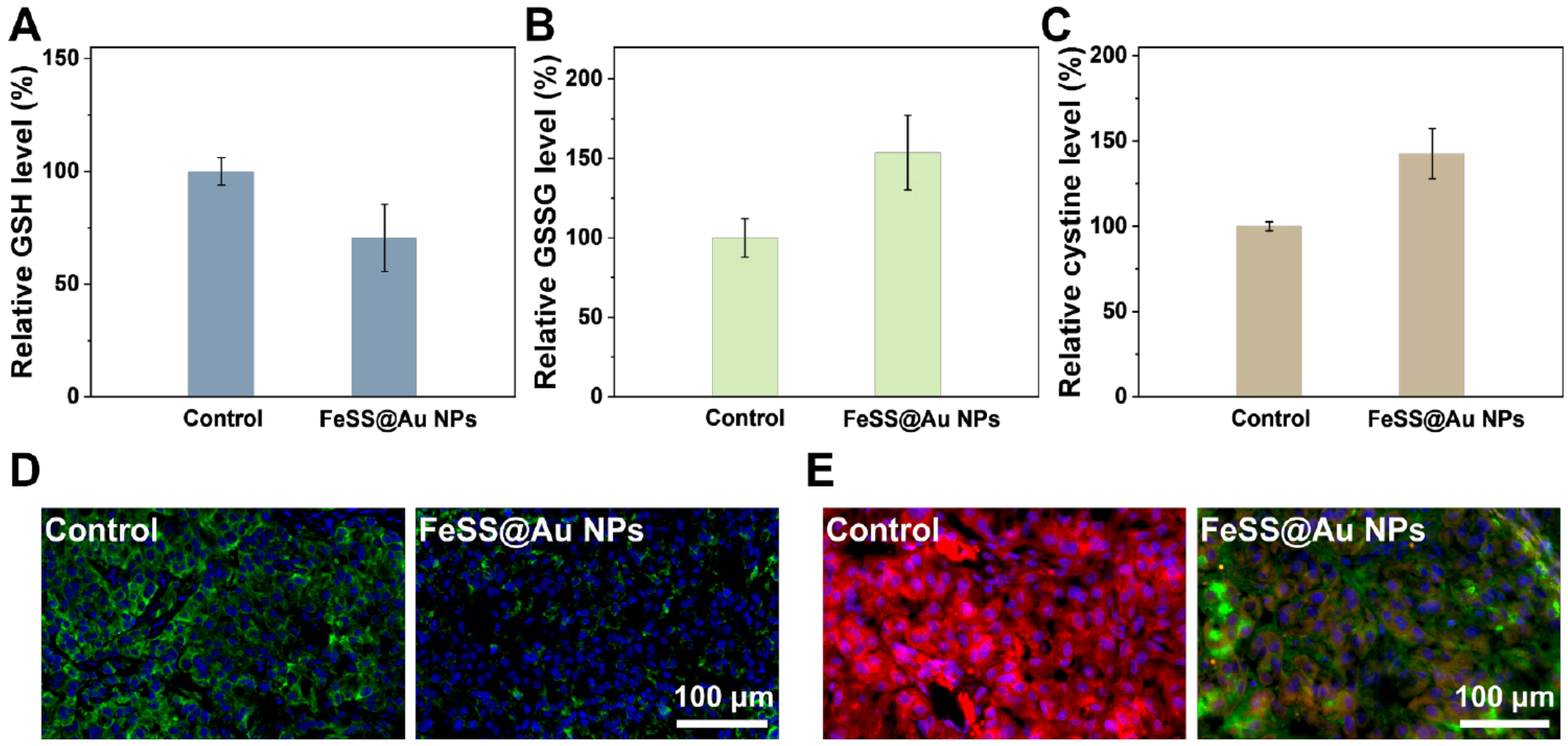

Figure S40. The GSH (A), GSSG (B) and cystine (C) levels in tumors of group I and III. The staining images of GPX4 (D) and C11-BODIPY$^{581/591}$ for LPO (E). The data in Figure S40A-C are presented as mean ± SD (n = 3).

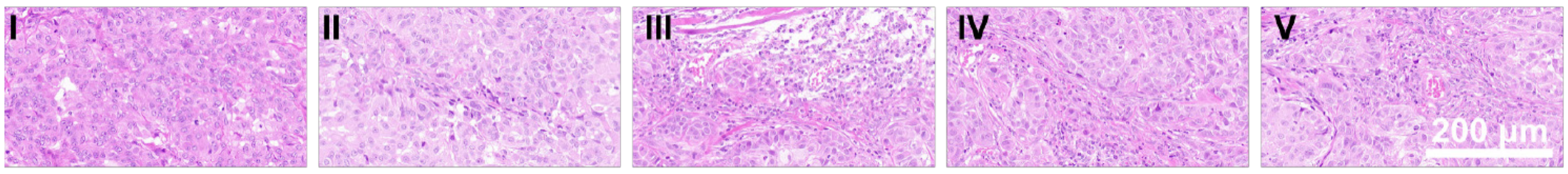

Figure S41. The H&E stained tumor slices of group I-V.

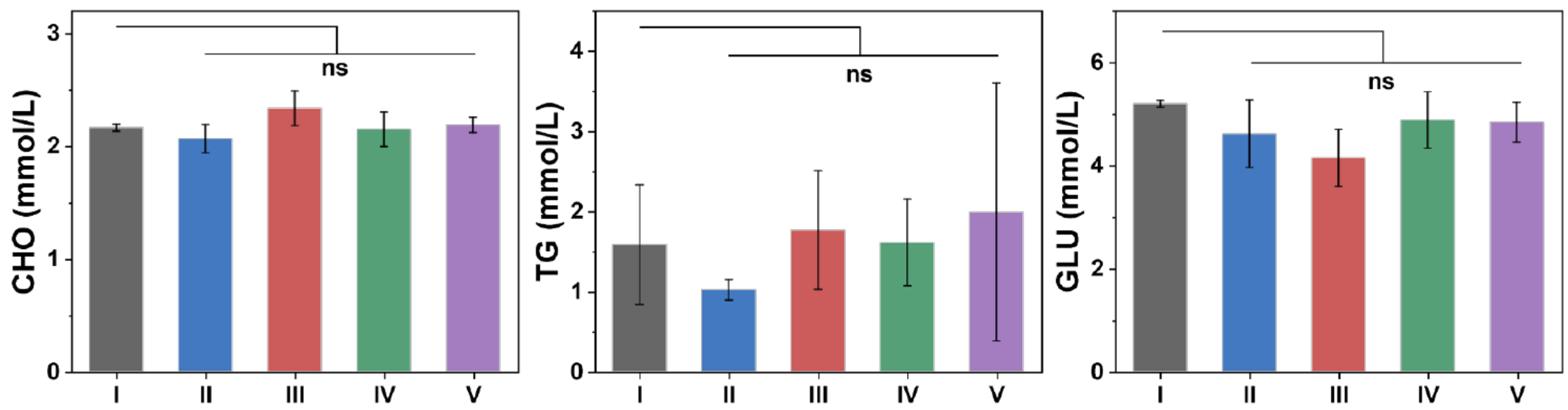

Figure S42. The results of blood lipid (CHO and TG) and blood glucose (GLU) of group I-V. The data are presented as mean ± SD (n = 3) with one-way ANOVA test.

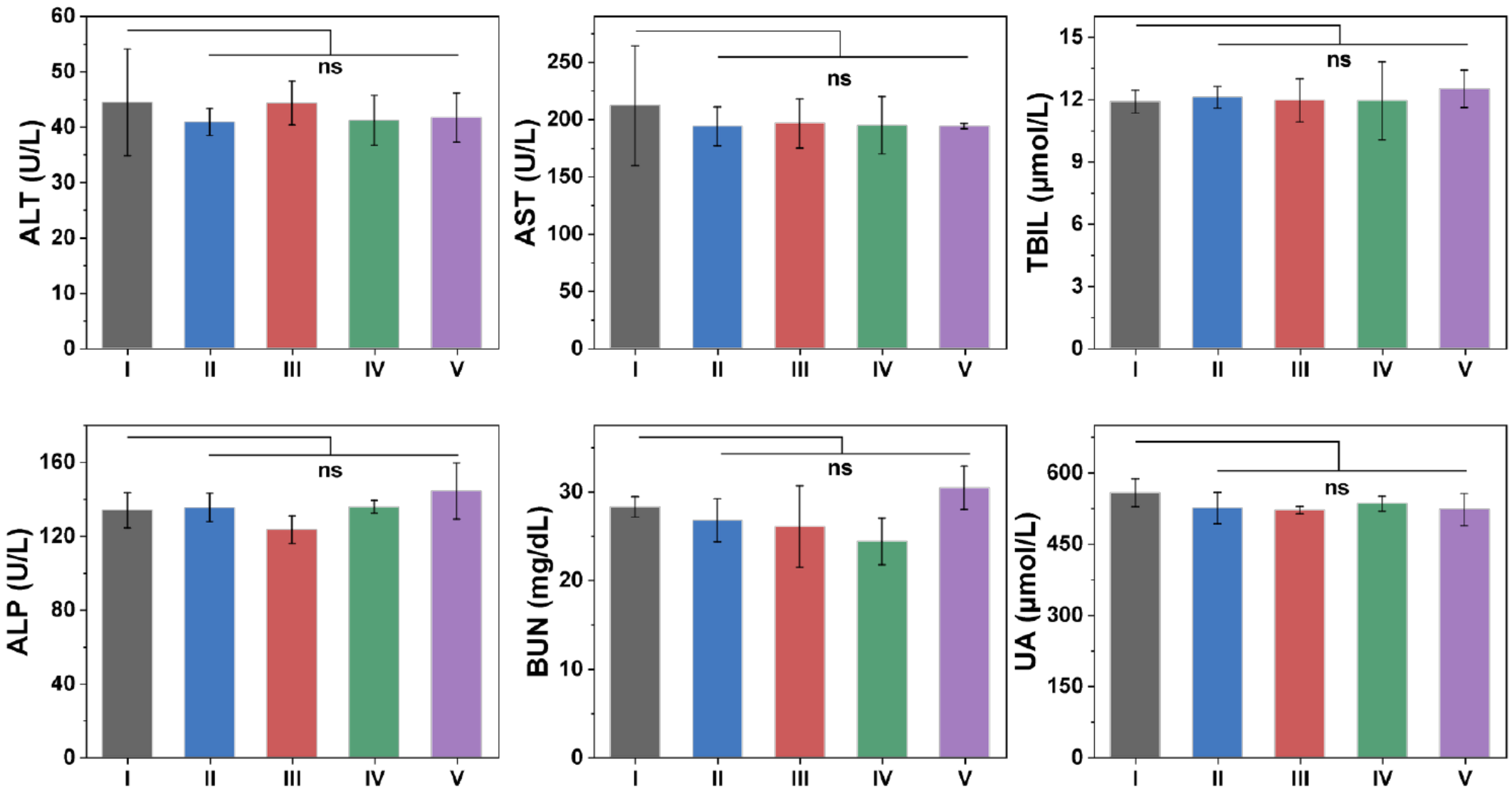

Figure S43. The results of liver function (ALT, AST, TBIL and ALP) and kidney function (BUN and UA) of group I-V. The data are presented as mean ± SD (n = 3) with one-way ANOVA test.

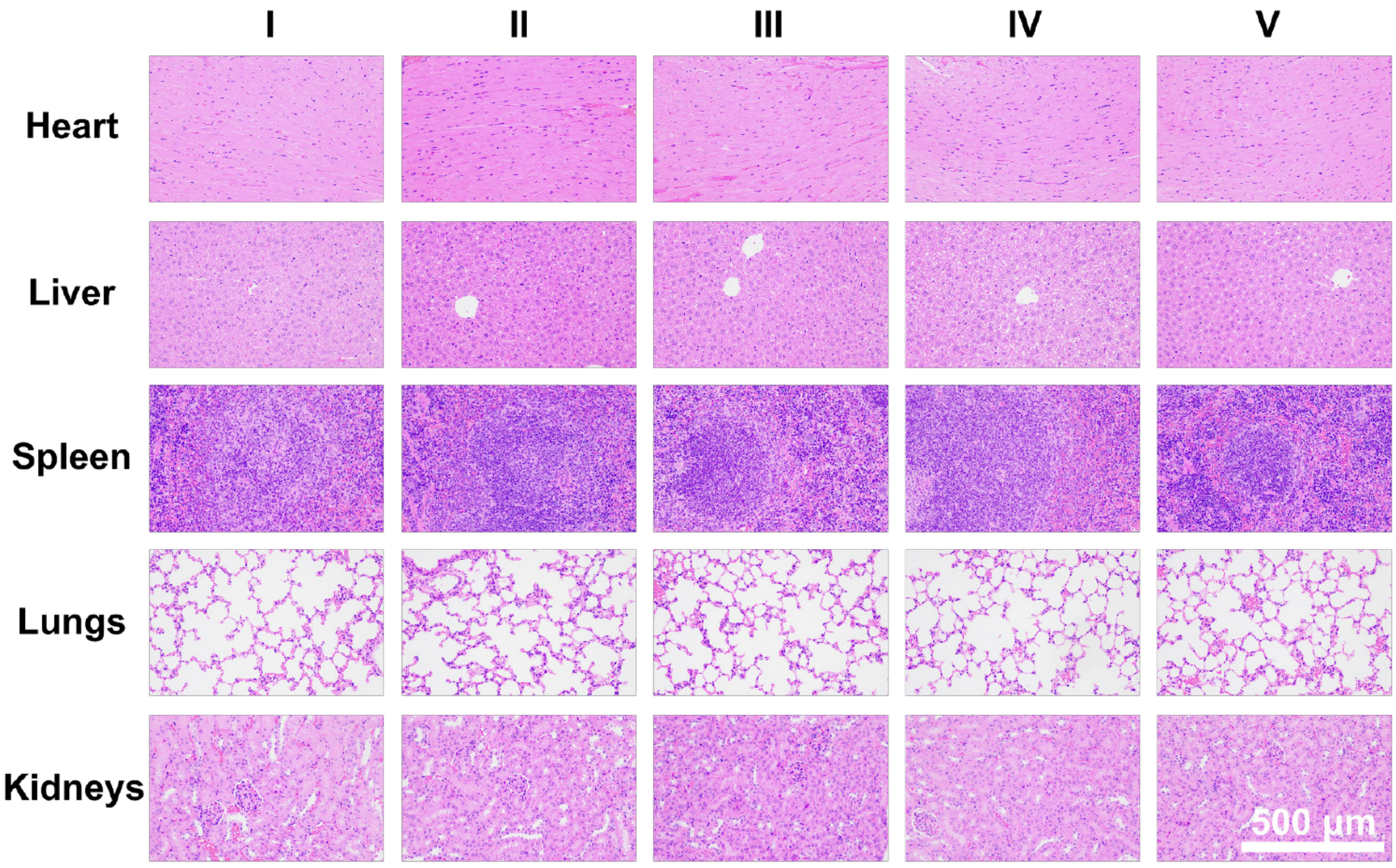

Figure S44. The H&E stained slices of major organs (heart, liver, spleen, lungs and kidneys) in group I-V.

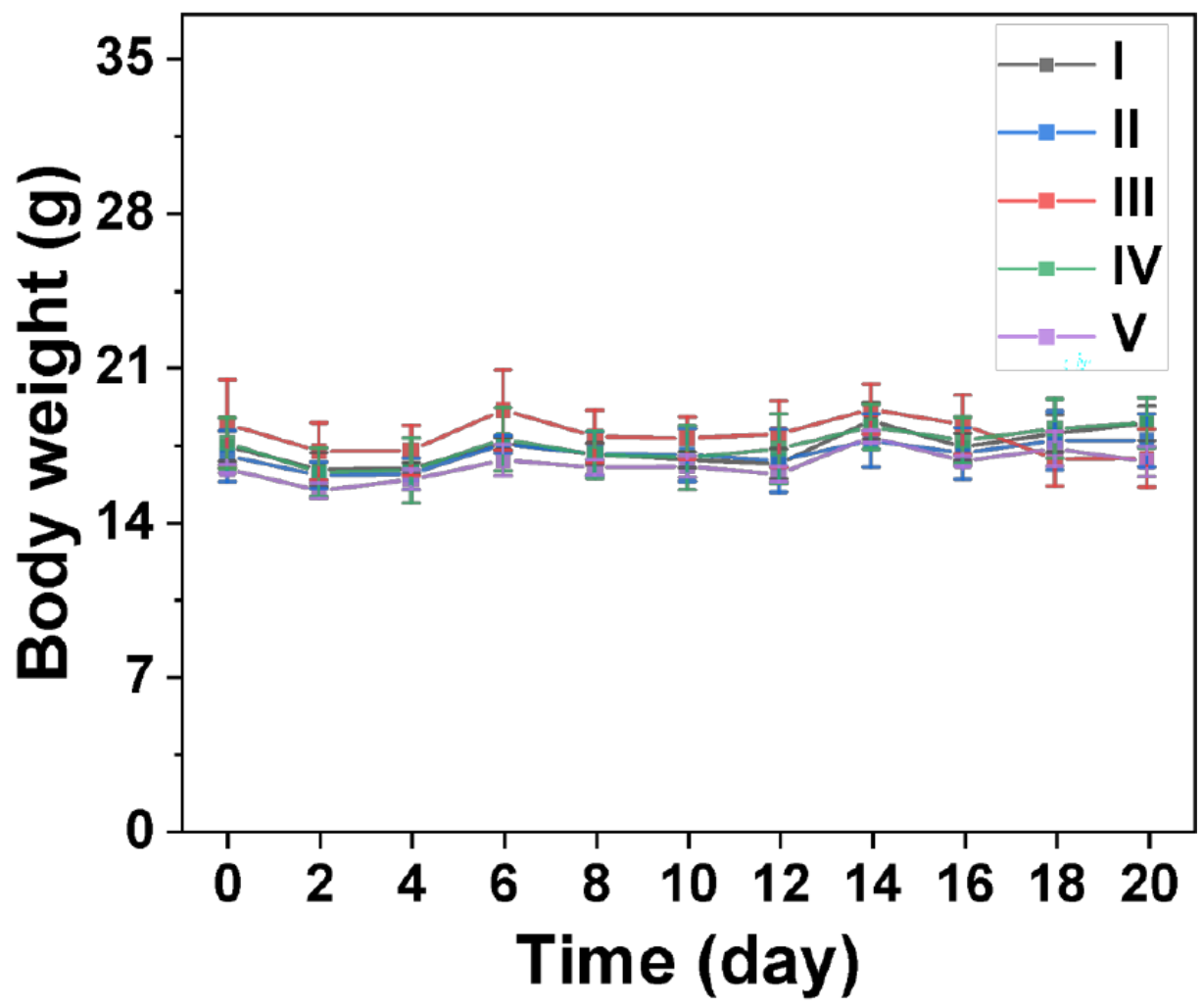

Figure S45. The body weight of mice in group I-V. The data are presented as mean ± SD (n = 5).

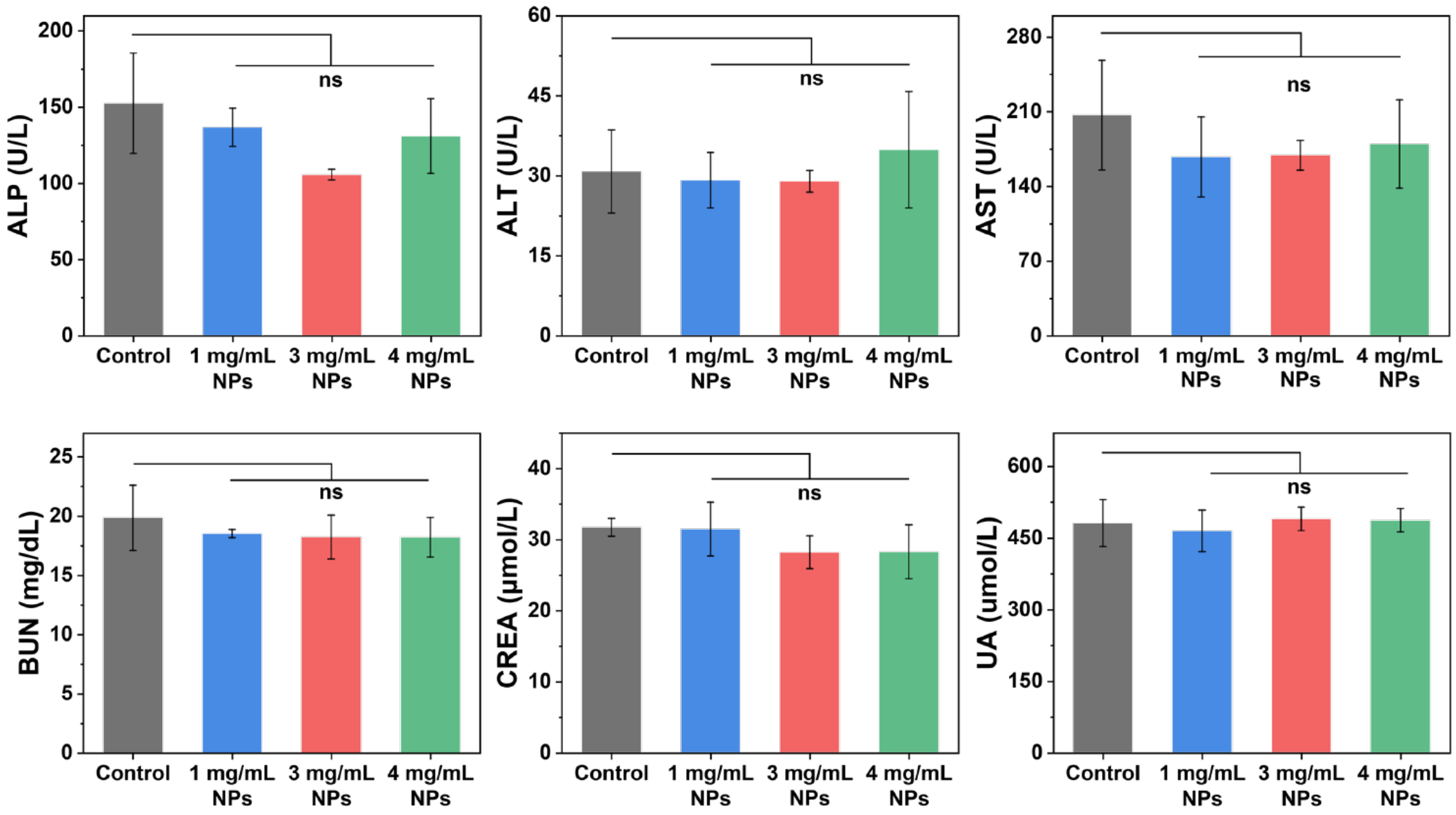

Figure S46. The results of liver function (ALP, ALT and AST) and kidney function (BUN, CREA and UA) in groups treated with FeSS@Au NPs of 0~4 mg/mL. The data are presented as mean ± SD (n = 3) with one-way ANOVA test.
